# Proteogenomic insights into the biology and treatment of pancreatic ductal adenocarcinoma

**DOI:** 10.1186/s13045-022-01384-3

**Published:** 2022-11-25

**Authors:** Yexin Tong, Mingjun Sun, Lingli Chen, Yunzhi Wang, Yan Li, Lingling Li, Xuan Zhang, Yumeng Cai, Jingbo Qie, Yanrui Pang, Ziyan Xu, Jiangyan Zhao, Xiaolei Zhang, Yang Liu, Sha Tian, Zhaoyu Qin, Jinwen Feng, Fan Zhang, Jiajun Zhu, Yifan Xu, Wenhui Lou, Yuan Ji, Jianyuan Zhao, Fuchu He, Yingyong Hou, Chen Ding

**Affiliations:** 1 Institute of Biomedical Sciences, State Key Laboratory of Genetic Engineering and Collaborative Innovation Center for Genetics and Development, School of Life Sciences, Human Phenome Institute, Department of Pathology, Department of General Surgery, Zhongshan Hospital, Fudan University, Shanghai, 200433 China; 2 State Key Laboratory of Proteomics, Beijing Proteome Research Center, National Center for Protein Sciences, Beijing, 102206 China; 3 Institute for Development and Regenerative Cardiovascular Medicine, MOE-Shanghai Key Laboratory of Children’s Environmental Health, Xinhua Hospital, Shanghai Jiao Tong University School of Medicine, Shanghai, 200092 China; 4 Department of Anatomy and Neuroscience Research Institute, School of Basic Medical Sciences, Zhengzhou University, Zhengzhou, 450001 China; 5 Research Unit of Proteomics Driven Cancer Precision Medicine, Chinese Academy of Medical Sciences, Beijing, 102206 China

**Keywords:** Pancreatic ductal adenocarcinoma, Proteomics, Phosphoproteomics, Genome, RNA-seq, Proteomic subtype, Immune subgroup, Diabetes, Metastasis

## Abstract

**Background:**

Pancreatic ductal adenocarcinoma (PDAC) is a devastating disease with poor prognosis. Proteogenomic characterization and integrative proteomic analysis provide a functional context to annotate genomic abnormalities with prognostic value.

**Methods:**

We performed an integrated multi-omics analysis, including whole-exome sequencing, RNA-seq, proteomic, and phosphoproteomic analysis of 217 PDAC tumors with paired non-tumor adjacent tissues. In vivo functional experiments were performed to further illustrate the biological events related to PDAC tumorigenesis and progression.

**Results:**

A comprehensive proteogenomic landscape revealed that *TP53* mutations upregulated the CDK4-mediated cell proliferation process and led to poor prognosis in younger patients. Integrative multi-omics analysis illustrated the proteomic and phosphoproteomic alteration led by genomic alterations such as *KRAS* mutations and *ADAM9* amplification of PDAC tumorigenesis. Proteogenomic analysis combined with in vivo experiments revealed that the higher amplification frequency of *ADAM9* (*8p11.22*) could drive PDAC metastasis, though downregulating adhesion junction and upregulating WNT signaling pathway. Proteome-based stratification of PDAC revealed three subtypes (S-I, S-II, and S-III) related to different clinical and molecular features. Immune clustering defined a metabolic tumor subset that harbored *FH* amplicons led to better prognosis. Functional experiments revealed the role of FH in altering tumor glycolysis and in impacting PDAC tumor microenvironments. Experiments utilizing both in vivo and in vitro assay proved that loss of HOGA1 promoted the tumor growth via activating LARP7-CDK1 pathway.

**Conclusions:**

This proteogenomic dataset provided a valuable resource for researchers and clinicians seeking for better understanding and treatment of PDAC.

**Supplementary Information:**

The online version contains supplementary material available at 10.1186/s13045-022-01384-3.

## Background

Pancreatic ductal adenocarcinoma cancer (PDAC) is a highly fatal disease that is the third leading cause of cancer-related deaths in the USA, and the sixth in China [1, 2]. PDAC has been predicted to become the second leading cause of cancer mortality in China by the year 2030 [3]. Despite the advancement in pancreatic cancer research, the mortality-to-incidence ratio has not experienced significant revision over the last few decades. The five-year survival rate remains just around 11% in the USA [1]. This grim prognosis is mainly caused by the lack of visible and distinctive symptoms and reliable biomarkers for early diagnosis as well as aggressive metastatic spread leading to poor response to treatments [4]. Consequently, there is an urgent need to better understand the molecular characteristics that render the development of novel diagnostic and therapeutic strategies for PDAC.

Epidemiology studies have indicated long-standing diabetes as a risk factor for PDAC. Stevens et al. [5] performed a meta-analysis which have demonstrated that the risk of PDAC was twice that in patients with type one diabetes. Another study conducted by V. A. Grote has indicated the HbA1c (an index for diabetes) could be used as a potential biomarker of early detection in pancreatic cancer [6]. Recent genomic studies revealed that PDAC patients with diabetes history might harbor some distinctive genomic alterations that affected their prognosis [7]. However, the mechanism of how long-standing diabetes affects patients’ prognosis in PDAC remains unclear, which results in lack of currently effective treatments for PDAC patients with the history of diabetes. Thus, a deeper characterization of genomic and proteomic associations of PDAC patients with diabetic history will lay a function for deciphering this mechanism and provide new knowledge for researchers.

A significant proportion of PDAC patients are diagnosed at an advanced stage, with established invasion and metastasis of important nearby structures, causing them to be ineligible for surgical interventions. Genomic studies conducted by McDonald et al. indicated the somatic variation in metastases including *TP53*, *SMAD4*, *ARID1A*, and *ATM* [8]. However, it is still functionally ambiguous how these metastasis-related variations impact the progression of PDAC. Hence, a metastatic marker based on multi-omics data may accurately reflect the metastatic behavior of PDAC, thus aiding the accurate prediction of patient prognosis.

The tumor microenvironment induced by interactions, especially the metabolic crosstalk between pancreatic epithelial/cancer cells and stromal cells is critical for pancreatic cancer progression and has been implicated in the failure of chemotherapy, radiation therapy and immunotherapy [9]. Recent studies have indicated several critical metabolic pathways that branch from glycolysis, including the pentose phosphate pathway (PPP), hexosamine biosynthesis pathway (HBP), serine biosynthesis, and tricarboxylic acid cycle (TCA cycle), significantly associates with PDAC tumorigenesis, and patients’ prognosis [10]. Despite the progression, the mechanism underlies genomic variations drove metabolic alteration and affected patients’ survival has not been illustrated.

Previous genomic studies, including The Cancer Genome Atlas (TCGA) program, have related genetic, gene expression, and DNA methylation signatures with patients’ prognosis in PDAC [11–14]. For example, exome and CNA analyses of PDAC have revealed a complex mutational landscape. The hotspot mutations of *KRAS*, *TP53, SMAD4*, and *CDKN2A* have been identified [13]. However, the molecular mechanism underlying gene alterations that drive cancer phenotypes in PDAC are largely unknown, which inhibits the identification of actionable therapeutical targets.

Over the last 10 years, remarkable progress has been made in PDAC proteomics studies. Iuga et al. recently found 99 proteins that overexpressed in tumors, of which prolargin (PRELP) might be a potential new prognostic biomarker [15]. Another quantitative proteomics experiment was performed using iTRAQ labeling and identified dihydropyrimidinase-like 3 (DPYSL3) which had the greatest diagnostic potential [16]. Cao et al. described a proteogenomic study of 140 PDAC patients and indicated how alterations such as *TP53*, *KRAS*, *CDKN2A*, and *SMAD4* affected the downstream biological events at proteomics level [17]. Despite these progresses, there is still a large percentage of PDAC patients without available targeted therapeutic options. Furthermore, the proteogenomic events that associated with clinical characteristics of the PDAC patients, such as long-standing diabetes or metastasis, are also remained unknown.

Here, we presented a proteogenomic analysis of 217 PDAC tumors with paired non-tumor adjacent tissues. Integrative analysis revealed proteomic and phosphoproteomic alterations led by *KRAS* mutations and other driving genomic alterations of PDAC. Correlation analysis between transcriptomics and proteomics revealed the concordance in regulating key pathways in tumor tissues and NATs. Proteogenomic analysis combined with functional experiments has proved that amplification of *ADAM9* (located on *8p11.22*) could promote metastasis, through downregulating adhesion junction and upregulating WNT signaling pathway. The proteomic classification demonstrated a subgroup with the poorest prognosis was featured with amplification of *GRB7* and upregulated ERBB signaling pathway and cell cycle. Immune clustering defined a metabolic tumor subset that harbored *FH* amplicons led to better prognosis. Functional experiments revealed the role of FH in altering tumor glucose metabolism and in impacting PDAC tumor microenvironments. Furthermore, we found that the downregulation of HOGA1 correlated with poor prognosis of PDAC. Mechanistically, HOGA1, collaborated with LARP7, played a key role in arresting PDAC proliferation via inhibiting cyclin kinases.

## Results

### Comprehensive proteogenomic characterization of PDAC cohort

**Fig. 1:**
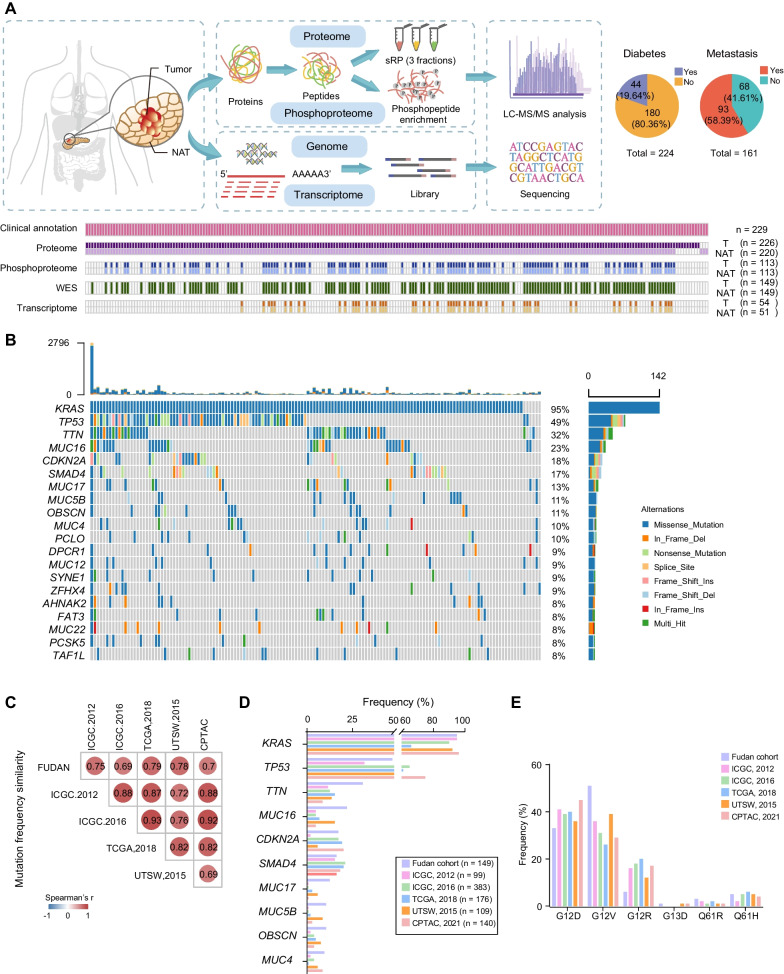
Multi-omics landscape of PDAC Samples. **A** Overview of the experimental design and the number of samples for the genomic, transcriptomic, proteomic, and phosphoproteomic analyses. **B** The genomic profiles of PDAC. The top 20 mutated genes and their occurrence in 149 PDAC patients and the mutation frequencies are shown. Mutation types and their frequencies are demonstrated by a bar plot in the right panel. **C** Spearman’s correlation plot indicating the mutation frequencies observed in the Fudan cohort compared to previously published cohorts. **D** Comparisons of mutation frequencies of top 10 mutated genes in the Fudan cohort and previously published cohorts. **E** Bar plots of the common *KRAS* driver mutations in Fudan cohort and previously published cohorts. ****p < 1.0E−4, ***p < 1.0E−3, **p < 1.0E−2, *p < 0.05, ns > 0.05

To characterize the proteogenomic landscape of pancreatic ductal adenocarcinoma (PDAC), whole-exome sequencing (WES), RNA-seq, proteomic, and phosphorylation proteomic data were collected from 229 treatment-naive patients (Additional file 23: Table S1A). HE-stained slides were examined and evaluated independently by two experienced pathologists and information regarding tumor histological subtype, degree of differentiation, TNM stage, and tumor purity were provided (Methods). All formalin-fixed paraffin-embedded (FFPE) samples used in this study had tumor purity ranged from 10 to 80% (median 50%) (Additional file 23: Table S1B). Neoplastic cellularity was evaluated independently by whole-exome sequencing using the ABSOLUTE [79] and ESTIMATE [78] algorithm (Methods) and ranged from 10 to 60% (median 36%), 1% to 70% (median 50%), respectively (Additional file 23: Table S1B). Computational purity showed significantly positive correlation with our histological evaluated tumor purity (Spearman’s = 0.98 for ABSOLUTE and 0.91 for ESTIMATE, *P* < 0.0001) (Additional file 19: Fig. S19A). A schematic of the experimental design is shown in Fig. 1A, and the clinical metadata are shown in Additional file 23: Table S1B. Clinical data, including the gender, age at diagnosis, tumor grade, and survival, etc., are summarized in Table 1. Comparing to recently published PDAC dataset conducted by CPTAC [17], our cohort showed distinctive demographic and clinical characteristics. Demographically, all the patients in our cohort were from Asian (Asian, n = 229, 100%), whereas 21 patients in CPTAC cohort were from Asian (Asian, n = 21, 15%); histologically, more early-stage patients (our cohort: stage IA/IB, n = 105, 46%, CPTAC cohort: stage IA/IB, n = 23, 16%; fisher exact test, *P* < 1.0E−4) were included in our cohort, which facilitated us to investigate the specific molecular features of early-stage PDAC for tumor diagnosis. Additionally, other risk factors and information associated with patients’ prognosis, such as metastasis status and diabetes, were also collected via follow-up in our study (Fig. 1A, Table 1 and Additional file 1: S1B). Proteomic profiling was performed on the 226 tumor and 220 non-tumor adjacent tissues (NATs). The WES analysis was conducted on the 149 paired samples, and the phosphoproteomic analysis was performed on the 113 paired samples, respectively. In addition, the mRNA sequencing was carried out on the 54 tumors and 51 paired NATs. Therefore, our study provided a comprehensive landscape of PDAC at the multi-omics level.

**Table 1:** Demographic characteristics of the patients in Fudan cohort and CPTAC cohort

Variable	Fudan cohort (n = 229)	CPTAC cohort (n = 140)
Participant area		
East Asia	229 (100%)	21 (15%)
Europe	0	60 (43%)
America	0	47 (34%)
Other	0	12 (9%)
Sex		
Male	133 (58%)	74 (53%)
Female	96 (42%)	66 (47%)
Age		
≤ 60	89 (39%)	40 (29%)
> 60	140 (61%)	100 (71%)
TNM stage ****		
IA	28 (12%)	7 (5%)
IB	77 (34%)	16 (11%)
IIA	16 (7%)	8 (6%)
IIB	89 (39%)	52 (37%)
III	19 (8%)	42 (30%)
IV	0	9 (7%)
Not sure	0	6 (4%)
Location		
Pancreaticoduodenum	145 (63%)	105 (75%)
Pancreatic body and tail	84 (37%)	33 (24%)
Not sure	0	2 (1%)
History of diabetes		
Yes	44 (19%)	39 (28%)
No	183 (80%)	101 (72%)
Not sure	2 (1%)	0
Metastasis (follow-up)		
Yes	93 (40%)	NA
No	68 (30%)	NA
Not sure	68 (30%)	NA

****p < 1.0E−4, Fisher exact test

WES profiling identified 19,530 genetic variation events (14,656 missense, 869 nonsense, 1976 in-frame, and 2029 frame-shift mutations). Significantly mutated genes (SMGs) were determined using OncodriveCLUST (Methods) [18], and mutations, such as *KRAS* (95%), *TP53* (49%), *TTN* (32%), *MUC16* (23%), *CDKN2A* (18%), and *SMAD4* (17%), were identified as the top-ranked mutations, in our cohort (Fig. 1B). Correlation analysis using mutational frequencies from other pancreatic carcinoma (PC) cohorts, including TCGA cohort [11], UTSW cohort [12], ICGC cohort [13, 14], and CPTAC cohort [17], resulted in an average of Spearman’s correlation coefficient was 0.8 among different cohorts, in which no significant difference was observed (Fig. 1C, Additional file 24: Table S2C-H). In addition, the mutational frequencies of *KRAS*, *TP53*, *CDKN2A*, and *SMAD4* were 95%, 49%, 18%, and 17% in our cohort, respectively, which showed similarity across the different cohorts (Fig. 1D). For *KRAS* mutations, except for the most common mutations of *KRAS* G12 (90.3%), we also identified the other *KRAS* codon mutations *KRAS* (G13D), *KRAS* (Q61H) (Fig. 1E).

The frequencies of mutated trinucleotide sequence motifs were analyzed using nonnegative matrix factorization (NMF) [19, 20] (Methods). Moreover, cosine similarity analysis against mutational signatures in human cancer was performed to reveal the potential contribution of endogenous and exogenous mutagens in PDAC, and five mutational profiles were identified. As a result, the mutational signatures best matching to those in our patients was SBS1, SBS 6 and SBS30 (Additional file 1: Fig. S1E). The same enrichment analysis was performed in the CPTAC cohort [17]. As a result, the mutational signatures best matching to those in CPTAC patients was SBS 1 and 6, associated with patients age at diagnosis (SBS1), and tumor mutation burden (SBS6) (Additional file 1: Fig. S1E and F).

As for proteomic analysis, 16,584 proteins were identified, with 9006 proteins per sample on average (Additional file 1: Fig. S1H-J) (Methods). Whole cell extract of HEK293T cells was used as quality control (QC) for mass spectrometers. This extract showed the robustness and consistency of the mass spectrometer, which was evidenced by a high Spearman’s correlation coefficient (r > 0.88) between the proteomes of QC samples (Additional file 1: Fig. S1G). Principal component analysis of proteomic data was conducted based on years of sample collection and experimental batches and found no obvious distinction, indicating that there was no batch effect (Additional file 1: Fig. S1L). Further, 15,443 and 15,092 proteins were identified in the tumors and NATs, respectively (Additional file 1: Fig. S1B). A total of 18,883 and 17,098 transcripts were identified in the tumors and NATs, giving an opportunity to probe the relationship between the transcriptome and proteome (Additional file 1: Fig. S1A).

Phosphoproteomic analysis identified 33,426 phosphosites including 24,470 (73.2%) on serine, 7914 (23.7%) on threonine, and 1042 (3.1%) on tyrosine, from 6153 phosphoproteins in 113 tumors; 33,696 phosphosites, including 24,721 (73.4%) on serine, 7965 (23.6%) on threonine, and 1010 (3.0%) on tyrosine, from 6243 phosphoproteins in 113 NATs (Additional file 1: Fig. S1C-D, Additional file 7: Fig. S7A). Comparing the phosphorylation sites S/T/Y distribution between HCC cohort [21], GC cohort [22], and CRC cohort [23], the ratio of S/T/Y in our cohort is similar to those cohorts (HCC cohort: 77.8%, 16.9%, and 5.3%, GC cohort:74.0%, 20.9%, and 5.1%, CRC cohort: 76.2%, 19.9%, and 3.9%), indicating that the ratio of S/T/Y in gastrointestinal tumors (including pancreatic cancer, liver cancer, gastric cancer, colon cancer, etc.) is comparable (Additional file 7: Fig. S7A-B). Importantly, the phosphorylation of some key molecules in tumor tissue was significantly enriched, providing an opportunity to explore key oncogenic phosphorylation modifications. For example, RB1 (retinoblastoma-associated protein) at S807, which could promote cell cycle progression in cancer cells [24], YAP1 at S109, which has been reported associated with tumor metastasis [25], was significantly overexpressed in tumor tissues. We further presented the MS2 spectrums of these phosphosites to verify the accuracy of our detection. Additionally, we utilized IHC (immunohistochemistry) with phosphorylation antibodies targeted to YAP1_pS109 and RB1_pS807 and confirmed their enrichment in tumor tissues (Additional file 2: Fig. S2A-C).

Thus, our study has so far established a comprehensive landscape of PDAC at the genomic, transcriptomic, proteomic, and phosphoproteomic levels (Additional file 1: Fig. S1K).

### The impacts of somatic copy number alterations in PDAC cohort

**Fig. 2:**
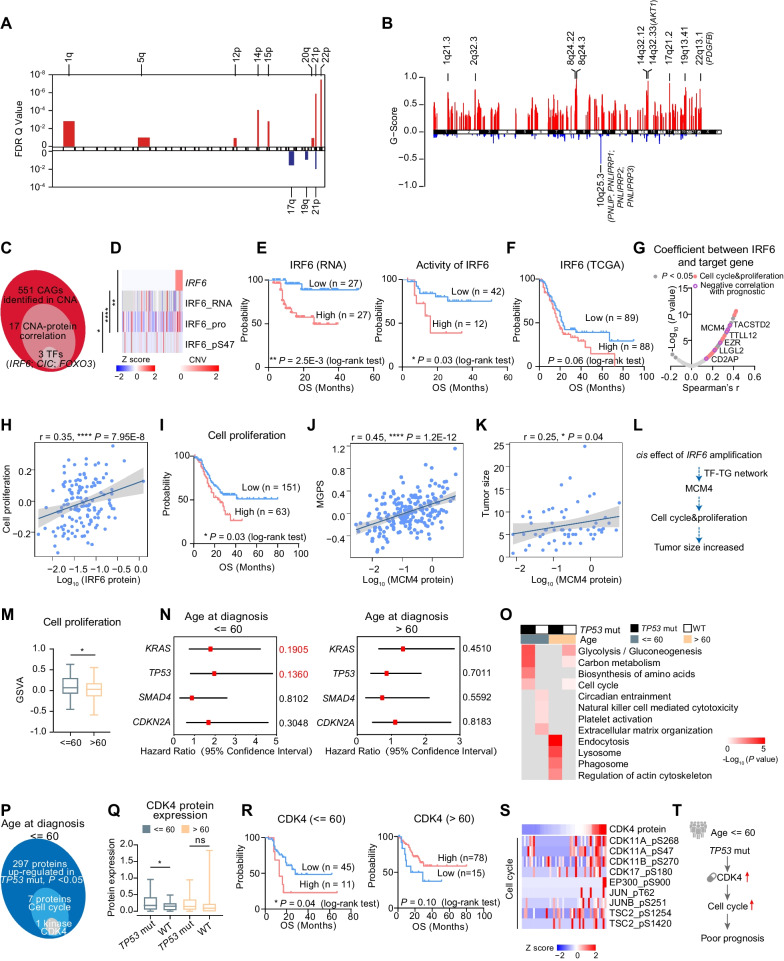
The Impacts of Somatic Copy Number Alterations in PDAC Cohort. **A** Significant arm-level focal peaks detected using GISTIC. **B** Focal-level SCNA events. Focal peaks with significant copy-number gains (red) and losses (blue) (GISTIC2 FDR < 0.05) are shown. The focal peaks are highlighted in approximate positions across the genome. **C** Venn diagram depicting the process of screening for TFs with *cis*-effect between CNA and protein. **D** The heatmap showing the *cis*-effect of IRF6 among CNA, RNA, protein, and phosphosite (Spearman’s correlation). The *P* value of correlation is noted on the left. **E** Kaplan–Meier curves for overall survival based on mRNA abundance of IRF6 (left), and TF activity of IRF6 (right) in Fudan cohort (log-rank test). **F** Kaplan–Meier curves for overall survival based on mRNA abundance of IRF6 in TCGA-PAAD dataset (log-rank test). **G** Spearman-rank correlation of the abundance of IRF6 and its target genes (Spearman’s correlation). The significant correlations are colored in dark gray. Cell cycle and cell proliferation-associated proteins are labeled in pink, and prognostic related proteins are labeled with purple circles. **H** The scatter plot describing the Spearman’s correlation between IRF6 protein expression and GSVA score of cell proliferation. **I** Kaplan–Meier curves for overall survival based on GSVA scores of cell proliferation (log-rank test). **J**, **K** The scatter plot describing the Spearman’s correlation between MCM4 protein expression and MGPS score (J) or tumor size (K). **L** The systematic diagram summarizing the impact of the *cis*-effect of *GRB7* amplification on increasing tumor size. **M** The boxplot revealing the comparison of GSVA score of cell proliferation between the younger patients (≤ 60) and the older patients (> 60) (Student’s t test). **N** The forest plot indicating the hazard ratio of *KRAS*, *TP53*, *SMAD4*, and *CDKN2A*, in younger patients (left) and older patients (right). **O** The pathway heatmap indicating the enriched pathways in the four groups (*TP53* mut, younger patients; WT, younger patients; *TP53* mut, older patients; WT, older patients). Each column represents a type of sample. The color of each cell shows -log_10_ transformed *P* value. **P** Venn diagram depicting the process of screening for highly expressed kinase in younger patients with *TP53* mutations. **Q** The boxplot revealing the comparison of CDK4 protein expression between *TP53* mutations and WT both in the younger patients (≤ 60) and the older patients (> 60) (Wilcoxon test). **R** Kaplan–Meier curves for overall survival based on CDK4 abundance in the younger patients (left) or the older patients (right) (log-rank test). **S** The heatmap showing cell cycle-associated phosphosites which are positively correlated with CDK4. **T** The systematic diagram summarizing the impact of *TP53* mutations on promoting cell cycle. ****p < 1.0E−4, ***p < 1.0E−3, **p < 1.0E−2, *p < 0.05, ns > 0.05

We applied GISTIC2 [26] to analyze the somatic DNA copy-number profiles of 149 PDAC samples. The most frequent gains were identified in chromosomes 14p (q = 8.4E−5), 21p (q = 1.4E−6) and 22p (q = 3.5E−8), and the most frequent losses were observed in chromosome 21p (q = 1.1E−2) and 17q (q = 2.8E−2) (Fig. 2A, Methods). In addition, we identified amplifications in driver oncogenes such as *AKT1* (14q32.33) and *PDGFB* (22q13.1) and deletions of the pancreas-specific genes such as *PNLIP and PNLIPRP1* (10q25.3) (Fig. 2B, Additional file 24: Table S2A-B).

The impacts of copy-number alterations (CNAs) on mRNA, protein, and phosphoprotein abundances in both *cis* or *trans* mode were characterized. In total, 1286 and 1277 significant positive correlations (*cis/trans*-effects) were observed for proteins and phosphoproteins, respectively (Spearman’s correlation, *P* < 0.05). The Kyoto Encyclopedia of Genes and Genomes (KEGG) pathway analysis indicated consistency among pathways subjected to enrichment by CNA-affected proteins, and phosphoproteins in the same sample type. For instance, CNA-affected proteins and phosphoproteins were enriched in ECM–receptor interaction, RNA transport, etc. (Fisher’s exact test, *P* < 0.05) (Additional file 3: Fig. S3A).

To further nominate functionally important genes within CNA regions, we focused on the 551 cancer-associated genes (CAGs) [27]. A total of 17 significant positive correlations were observed for proteins (Spearman’s correlation, *P* < 0.05), including 3 transcription factors (TFs: IRF6, CIC, and FOXO3) (Fig. 2C). IRF6, the top-ranked gene, was significantly associated with its cognate RNA, protein, and phosphorylation site (Fig. 2D). Further survival analysis indicated both the mRNA expression and the inferred TF activity of IRF6 were significantly associated with unfavorable overall survival. This observation was further supported by TCGA data (Fig. 2E, F). IRF6 is a transcription factor that has been reported to participate in various cellular process through transcription regulation [28]. Ke Zhang et al. have reported that IRF6 might be a prognostic marker in pancreatic cancer [29]. To gain great insight into the mechanism of how IRF6 led to poor prognosis, we applied correlation analysis on the target genes (TGs) of IRF6. As a result, the TGs significantly associated with the protein expression IRF6 were mainly enriched in cell cycle and cell proliferation. The expression of TGs including MCM4, TTL12, EZR, and the GSVA scores of cell cycle was positively correlated with the protein expression of IRF6 (Fig. 2G–H). Moreover, survival analysis indicated in concordant with the expression of IRF6, the enhanced cell proliferation also led to poor prognosis (Fig. 2I). These results suggested IRF6 could promote the cell proliferation through transcriptional regulation. Since enhanced cell proliferation could be represented by the elevated MGPS (multi-gene proliferation score) and enhanced tumor size, we evaluated the association between IRF6’s TGs and both MGPS and tumor size (Methods). As a result, the most significant positive correlations were observed between MCM4 (TG of IRF6) with both MGPS (correlation = 0.47, *P* = 6.7E−14) and tumor size (correlation = 0.25, *P* = 0.04), emphasized the important role of MCM4 in IRF6-mediated cell proliferation (Fig. 2J, K). In sum, our data revealed a potential mechanism that the *cis*-effect of *IRF6* promoted the progression of PDAC by influencing cell cycle process (Fig. 2L).

To explore other risk factors which might associate with tumor cell proliferation in PDAC, we further investigated the effect of diverse clinical characteristics (age, gender, etc.) on cell proliferation. PDAC is considered a disease of the elderly, with patients mostly over the age of 65 [30]. Individuals diagnosed with PDAC under the age of 60 are considered to be young-onset and potentially at high risk for a genetic predisposition. Intriguingly, we observed that the younger patients (age at diagnosis ≤ 60) in our cohort showed elevated GSVA scores of cell proliferation (Fig. 2M). To elucidate the possible causes and impacts of this phenomenon, we first compared the SCNAs, RNA, and protein expression of IRF6 between older and younger patients (older: > 60, younger: ≤ 60) and observed no significant difference (Additional file 3: Fig. S3B). Survival analysis indicated the elevated expression of IRF6 associated with poor prognosis in both older and younger patients (Additional file 3: Fig. S3C). We then compared the prognostic relevance of SMGs between the two groups and observed the mutations of *TP53* only impacted the overall survival in patients younger than 60 (Fig. 2N). Comparative analysis between *TP53*-mutant and *TP53*-wild-type patients, in the two age groups, further illustrated that in younger patient group, the cell cycle process was elevated in *TP53*-mutant patients, whereas, in older patient group, the cell cycle process was dominant in *TP53*-wild-type patients (Fig. 2O). To further illustrate the mechanism, underline how *TP53* mutations impact the cell cycle process, we performed comparative analysis and identified 7 cell cycle-related proteins (RB1, PRKDC, CDK4, MCM4, SMC1A, SKP1, and ANAPC2) which showed elevated expression in younger *TP53-*mutant patients (Fig. 2P, Q, Additional file 3: Fig. S3D). Further survival analysis revealed that the CDK4 was significantly associated with poor prognosis only in younger *TP53-*mutant patients (log-rank t test, *P* < 0.05) (Fig. 2R). To illustrate the impact of CDK4 on kinase–substrate phosphorylation, we screened out phosphosubstrates regulated by referred kinases from public database [31] and calculated the correlation between the abundance of these phosphosubstrates and the protein expression of CDK4. As a result, the abundance of phosphosites enriched in cell cycle such as CDK11A_pS268, CDK17_pS900, and EP300_pS900 were significantly associated with the expression of CDK4 (Fig. 2S, Additional file 3: Fig. S3E), suggesting that the CDK4 regulated the cell cycle process through phosphorylation signaling pathway. Together, this result revealed the role of *TP53* mutations in promoting tumor cell proliferation, through CDK4-mediated signaling pathway in younger patients, and suggested promising therapeutical options of younger *TP53*-mutant patients by CDK4 inhibitors (Fig. 2T).

### Phosphorylation of transcription factor E2F1 promoting the tumor cell proliferation feature of patients with KRAS^G12D^ mutations

*KRAS* is the well-known oncogene with the highest mutation rates in PDAC. Unfortunately, there are still no effective strategies targeting *KRAS* mutations. Due to the intrinsic characteristics of KRAS protein, targeting KRAS has been quite challenging [32]. Therefore, many efforts have focused on targeting it downstream signaling effectors.

To investigate signal transduction pathways downstream of activated KRAS in search of alternative therapeutic targets, we evaluated phosphosite expression changes in tumors with different *KRAS* hotspot mutations. As a result, comparing to tumors with *KRAS*^*G12R*^, *KRAS*^*G12V*^, *KRAS*^*Q61H*^, and *KRAS*^*Q61R*^ mutations, tumors with *KRAS*^*G12D*^ mutations harbored elevated phosphorylation of RAF1 at Ser 259, MAP2K2 at Tyr 222, MAPK1 at Ser188, and E2F1 at Ser 364, which indicated the elevation of RAS-RAF-MEK-ERK-E2F1 phosphorylation cascade in tumors with *KRAS*^*G12D*^ mutations, and E2F1 at Ser 364 had the most significant elevation among these phosphorylation sites (Additional file 4: Fig. S4A, B).

Functionally, E2F1, as a cell cycle-specific transcription factor (TF), has been reported to be highly expressed in a variety of tumors, and mainly participated in regulating tumor cell proliferation [33, 34]. To evaluate the prognostic value of elevated phosphorylation of E2F1, we conducted survival analysis and found that the elevated phosphorylation of E2F1 at Ser 364 was negatively associated with patients’ overall survival (Additional file 4: Fig. S4C), which emphasized its therapeutical potential in the future.

To further investigate the mechanism that how the elevated phosphorylation of E2F1 led to poor prognosis, we inferred the TF activity of E2F1 based on GSVA algorism (Methods) and found that along with the phosphorylation of E2F1 at Ser 364, its TF activity was also negatively associated with patients’ overall survival (Additional file 4: Fig. S4C). Intriguingly, the protein expression of E2F1 showed no difference among tumors with different *KRAS* hotspot mutations (Additional file 4: Fig. S4D). In supporting of the observation, we also conducted IHC staining utilizing both E2F1_pS364 antibody and E2F1 antibody and found only the phosphorylation of E2F1 showed significant elevation in tumors with *KRAS*^*G12D*^ mutations (Additional file 4: Fig. S4E). Consistently, the inferred TF activity of E2F1 showed high correlation with the abundance of phosphorylation of E2F1 at S364 but no correlation with the E2F1 protein expression (Additional file 4: Fig. S4F). These findings indicated the TF activity of E2F1 was contributed by phospho-E2F1 rather than the abundance of E2F1 protein.

We then hypothesized that the phosphorylation of E2F1 might impact prognosis in patients with *KRAS*^*G12D*^, through transcription regulation. Along with this hypothesis, we surveyed the expression of E2F1’s TGs and identified that the mRNA expression of TGs including AHCTF1, CDC27, ATM, TAOK3, RB1, and CDK14, which enriched in cell cycle process, was most significantly associated with the TF activity of E2F1 (Additional file 4: Fig. S4F). Interestingly, further correlation analysis revealed the significantly positive correlation between those TGs’ mRNA expression and their cognate protein expression, which revealed that the transcriptional regulatory pattern led by E2F1 was inherited at protein level (Additional file 4: Fig. S4G). We then performed survival analysis and observed the protein expression of TGs including AHCTF1, CDC27, MAP4K5, etc., which were also negatively associated with patients’ overall survival (Additional file 4: Fig. S4H).

In sum, our finding revealed the phosphorylation of E2F1 at S364, but not protein expression, elevated its TF activity which enhanced the cell proliferation process, and led to poor prognosis in tumors with *KRAS*^*G12D*^ mutations (Additional file 4: Fig. S4I).

### Integrated multi-omics features in tumor tissues compared with NATs of the PDAC

**Fig. 3:**
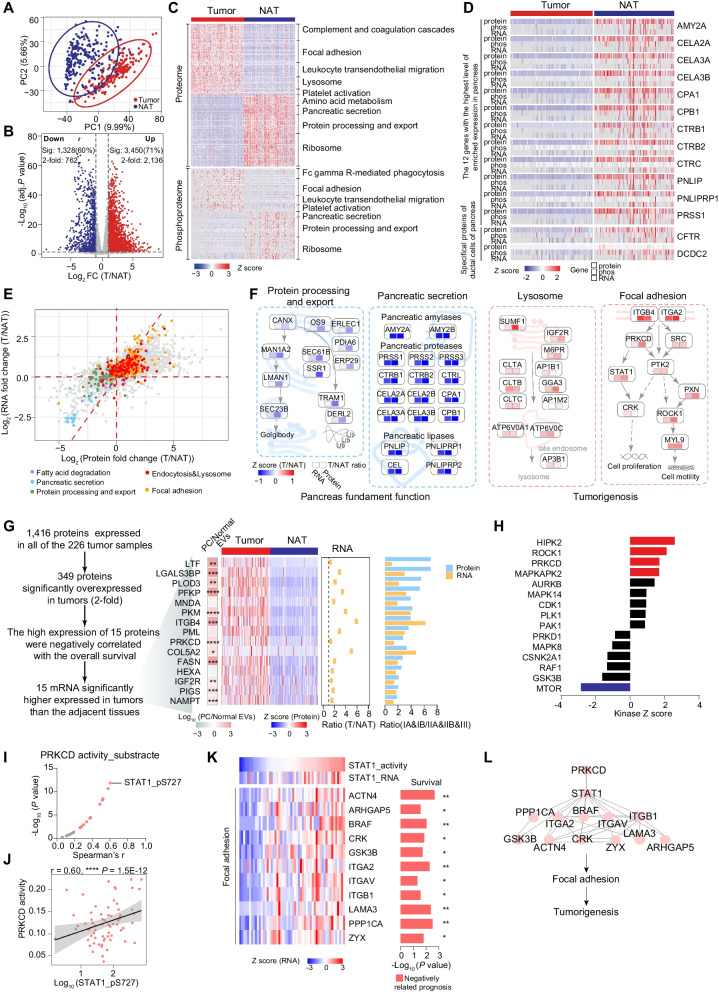
Integrated multi-omics features in tumor tissues Compared with NATs of the PDAC. **A** PCA of 7055 proteins in 226 tumors and 220 normal adjacent tissues (NATs). Red, tumors; blue, NATs. **B** A volcano plot showing the results of a two-tailed Student’s t test comparing tumors and NATs at proteomic level. The number of significantly increased and significantly decreased proteins in tumors is shown above the volcano plot. **C** Differentially expressed proteins and phosphoproteins in tumors and NATs. The enriched biological pathways are annotated on the right. **D** The expression of pancreatic signature proteins in tumors and NATs at multi-omics level (mRNA, protein, and phosphoprotein). **E** The scatter plot indicating the changes of protein and mRNA between tumors and NATs. Proteins are labeled based on the pathways they are enriched in (proteins participated in fatty acid degradation, pancreatic secretion, and protein processing and export are labeled in purple, light-blue, and green, respectively; protein participated in endocytosis and lysosome are labeled in red, and proteins participated in focal adhesion are labeled in orange). The systematic diagram summarizing proteins and signaling cascades that are significantly altered in NAT (protein processing and export, pancreatic secretion), and in tumor (lysosome, focal adhesion). Values are color coded based on the fold change between tumors and NATs at both transcriptomic and proteomic level. **F** The screening process of potential biomarkers in PDAC showing on the left. The expression heatmap describes the protein expression of potential biomarkers (middle). Color of each cell shows Z-scored average abundance of the protein across the tumor and NAT samples. The ratio of RNA expression between tumors and NATs is shown on the right, and the heatmap on the left indicates the fold change of proteins average expression between pancreatic cancer exosomes versus normal exosomes. Bar plot shows the ratio of protein and RNA expression of these biomarkers between early-stage patients (TNM IA and IB) and others (right). Proteins are labeled in blue, and RNAs are labeled in yellow. **G** KSEA analyses of kinase activities in tumors and NATs. **H** The volcano plot depicting the correlation between phosphosubstrates and the kinase activity of PRKCD; the significant correlations are colored in pink. **I** Spearman rank correlation of the abundance of phosphosite STAT1_pS727 and the kinase activity of PRKCD (Spearman’s correlation). **J** Heatmap of the relative abundance of focal-adhesion-related proteins (bottom panel) and STAT1 mRNA (top panel) that are significantly associated with TF activity of STAT1. The *P* value of survival is shown on the right. **K** The systematic diagram summarizing proteins and signaling cascades that are regulated by the kinase PRKCD. ****p < 1.0E−4, ***p < 1.0E−3, **p < 1.0E−2, *p < 0.05, ns > 0.05

Multi-dimensional omics data provided an excellent chance to explore the relationships between the transcriptome, proteome, and phosphoproteome of PDAC. After appropriate sample quality control (QC) and normalization procedures (Methods), we performed integrated multi-omics analysis to systematically represent the characteristics of PDAC. Principal component analysis (PCA) of transcriptome, proteome, and phosphoproteome data was conducted to demonstrate a clear distinction between tumors and NATs, which further highlighted the diverse expression patterns existing between pancreatic tumor tissues and normal adjacent tissues that emphasized our stratification analysis (Fig. 3A, Additional file 5: Fig. S5A-B).

To identify protein network associated with PDAC, we applied Student’s t test and identified 2136 (30%) and 762 (11%) proteins that were significantly overrepresented (FC > 2, Student’s t test, Benjamini–Hochberg adjusted p < 0.05) in the tumors, and NATs, respectively (Fig. 3B, Additional file 25: Table S3A). Further enrichment analysis of KEGG ontologies indicated that complement and coagulation cascades, focal adhesion, and platelet activation were significantly enriched in tumor tissues, whereas pathways related to previously reported pancreas physiology functions [35], including that amino acid metabolism, pancreatic secretion, protein processing, and export were enriched in NATs (Fig. 3C). The consistency of the dominant pathways enriched in tumors or in NATs was also observed at the transcriptome and phosphoproteome levels (Additional file 5: Fig. S5C-G, Additional file 25: Table S3B). This distinctive proteomic features of tumor and NATs were further supported by CPATC data [17]. To be more specific, 1918 proteins (56%) were identified to be upregulated and 963 proteins (72%) to be downregulated in both our cohort and CPTAC cohort, respectively (Additional file 6: Fig. S6A). Proteins enriched in ECM–receptor interaction and focal adhesion were significantly elevated in tumor tissues, while proteins enriched in pancreatic secretion, protein digestion, and absorption were significantly elevated in NATs (*P* < 0.05) in both CPTAC and Fudan cohort (Additional file 5: Fig. S5F and Additional file 6: Fig. S6B).

To understand the regulatory relationship among multi-omics, we calculated gene-wise (inter-sample) and sample-wise (intra-sample) correlation. Between transcriptome and proteome, NATs displayed a median gene-wise correlation value of 0.2, while tumors displayed a higher median value of 0.32 (Additional file 5: Fig. S5H-I). For PDAC tumors and NATs, 24% and 14% of mRNA-protein pairs had significant positive Spearman’s correlations, respectively (Additional file 5: Fig. S5H-I, Benjamini–Hochberg adjusted p < 0.05), with DNA repair, G2M checkpoint and lysosome, etc., positively correlated in tumors. In NATs, the metabolic process, such as oxidative phosphorylation, glycolysis, and pancreatic secretion, displayed a positive correlation pattern, representing the coherence of mRNAs and proteins in regulating the key pathways in tumor and NATs. In addition, although proteins participated in focal adhesion, lysosome, pancreatic secretion, and protein processing and export showed significant alteration between tumors and tumor-adjacent tissues both at proteome level and transcriptome level. The data showed more significant alteration between tumors and NATs at proteome level, highlighting the existence of a differentially regulated axis between transcriptome and proteome for the maintenance of carcinogenesis (Fig. 3E, Additional file 25: Table S3C). The significantly altered pathways and mapped key proteins are summarized in Fig. 3F.

Further correlation analysis between proteome and phosphoproteome showed that NATs displayed a median gene-wise correlation value of 0.12, while tumors displayed a higher median value of 0.26 (Additional file 5: Fig. S5J-K). For tumors and NATs, 14.7% and 32.3% proteins-phosphoprotein pairs had significant positive Spearman’s correlations, respectively (Additional file 5: Fig. S5J-K, Benjamini–Hochberg adjusted p < 0.05), with lipid metabolism, glycolysis, and pancreatic secretion, showed positive correlation in NATs. In tumors, the process that have been reported in PDAC tumorigenesis and metastasis such as cell cycle process, P53 pathway and WNT pathway displayed a positive correlation pattern, further revealed the concordance among different omics layer in regulating core process in tumors and NATs.

We further focused on the expression of pancreatic markers between tumors and NATs based on the human protein atlas database (https://www.proteinatlas.org/). Importantly, 68.5% (183/267) of detected pancreas-specific proteins, which mainly enriched in pancreatic-specific metabolic pathways such as pancreatic secretion, protein digestion and absorption, and fat digestion and absorption, were significantly attenuated in the tumors (Fig. S6C). Survival analysis indicated that 43.7% (80/183) showed positive association with overall survival. In addition, the proteins which had the highest level of enriched expression in pancreas (including CELA2A, CPA1, PRSS1 etc.) and specific proteins of ductal cells of pancreas (including CFTR and DCDC2) showed decreased expression at multi-omics level (Fig. 3D). Concordantly, the pancreas-specific proteins including CELA2A, PRSS1, and PNLIPRP1 were also detected to be enriched in NATs, in the CPTAC cohort [17], confirming the loss of pancreas functions could reflect the malignant of the PDAC (Additional file 6: Fig. S6D).

Our dataset provided a good opportunity to identify potential prognostic biomarkers and drug target of PDAC. We hypothesized that the most highly and commonly expressed proteins with prognostic power in tumor could be circulated and detected in body fluid or exosome samples, and could be used as potential biomarkers. Under this assumption, we performed differential gene expression analysis among 1416 proteins expressed in all the 226 tumor samples and identified 349 significantly altered proteins (FC > 2, *P* < 0.05), in which 15 proteins were negatively correlated with the overall survival. These 15 molecules were also significantly upregulated in tumors at transcriptome level. To further validate the prognostic value of the 15 proteomic biomarkers, we included proteomic and clinical data from CPTAC PDAC study, compared the protein expression level of these 15 proteins between tumors and NATs, and evaluated their relevance with patients’ overall survival. As a result, in concordant with our result, all 15 proteins showed elevated expression in tumors in CPATC cohort, and 14 of them were negatively associated with patients’ overall survival (Fig. 3G, Additional file 6: Fig. S6E). We then screened these 15 proteins’ expression in an Exosome database of pancreas cancer [36], to further confirm the clinical applicable of these potential biomarkers. As we expected, 12 out of 15 proteins were detected in the Exome database, and all of which showed elevated expression in PDAC patients (Student’s t test, *P* < 0.05), including some FDA approved drug targets such as NAMPT and PRKCD (Fig. 3G).

Kinases participate in many cellular processes through signaling transduction [37]. To dissect the dynamic changes of both kinases and phosphoproteins in PDAC, we performed kinase–substrate enrichment analysis (KSEA) (Methods), and four kinases (HIPK2, ROCK1, PRKCD, MAPKAPK2) were identified as tumor-specific activated. PRKCD, which we mentioned earlier as a potential biomarker, was also identified (Fig. 3H, Additional file 25: Table S3D). PRKCD has been reported to participate in various biological processes including ECM, and focal adhesion [38]. To further illustrate the impact of PRKCD on phosphoproteome, we calculated the correlation between the abundance of phosphosites and the protein expression of PRKCD (Methods). As a result, the phosphorylation of STAT1 at S727 was the top-ranked phosphosite positively correlated with kinase activity of PRKCD (Fig. 3I, J). Functionally, STAT1 is a transcription factor which regulates multiple pathways, and could be activated through phosphorylation [39]. To gain great insights into the downstream impacts of STAT1, we then performed correlation analysis between the inferred TF activity of STAT1 and the target genes’ expression. As a result, TGs of STAT1 including the core regulators of focal adhesion, such as BRAF, CRK, GSK3B, were significantly correlated with STAT1. Moreover, survival analysis revealed the negatively association between theses TGs’ expression with patients’ prognosis (Fig. 3K). In sum, this insight revealed the kinase PRKCD regulated ECM and focal adhesion in PDAC through phosphorylation of STAT1, and subsequent transcription regulation process driven by STAT1 (Fig. 3L). These observations revealed the important role of PRKCD in PDAC malignant progression and suggested that inhibition of PRKCD could be a potential therapeutical option of PDAC.

### Analysis of phosphotyrosine signaling identifies oncogenic phosphorylation features of PDAC

Compared to phosphoserine (S) and phosphothreonine (T), phosphotyrosine (Y) are more upstream and thus would have yielded a better depth for possible targets and have uncovered the activated pathways with a more clinical impact. We focused on 1604 phosphotyrosine and performed differential expression analysis between tumors and NATs. A total of 44 phosphotyrosine sites were significantly higher expressed in tumors than NATs, which were enriched in platelet activation, and focal adhesion. Additionally, 32 of 44 phosphosites modified at tyrosine which were significantly overexpressed in tumors, their cognate proteins (20 proteins) were also observed to be significantly elevated in tumor tissues (Benjamini–Hochberg adjusted p < 0.05). Importantly, more significant alteration between tumors and NATs were observed at phosphoproteome level, highlighting the existence of a differentially regulated axis between proteome and phosphoproteome for the maintenance of carcinogenesis (Additional file 7: Fig. S7C).

Furthermore, survival analysis indicated that 8 out of 20 proteins (PLEC-PLEC_pY4611; MAPK3-MAPK3_pY204, etc.) which showed both elevated expression at protein level and phosphotyrosine level, were significantly negatively correlated with prognosis. Several novel phosphotyrosine sites of reported PDAC-associated genes like PLEC at tyrosine 4611 and ENO1 at tyrosine 44 were identified, implied the therapeutical potential of those phosphotyrosine sites [40, 41] (Additional file 7: Fig. S7D). Intriguingly, the phosphorylation of PLEC at tyrosine 4611 was also identified as the most significantly overexpressed phosphotyrosine site in metastatic patients, suggesting the potential association between phosphorylation of PLEC at Y4611 and PDAC metastasis. Intriguingly, to illustrate the phosphorylation cascade associated with PLEC_pY4611, we investigated the correlation between the protein expression of kinases and the phosphorylation of PLEC_pY4611. As a result, the expression of kinase PRKCZ showed significantly positive correlation with the phosphorylation of PLEC_pY4611, implying the regulatory role of PRKCZ on phosphorylation of PLEC at Tyr 4611. This finding was further convinced by the kinase prediction analysis utilizing NetworKIN [42], which also indicated that PRKCZ might phosphorylating PLEC at Tyr 4611 (predication score > 0.9). We further examined the protein expression of PLEC and there was observed no significant difference in protein expression of PLEC between metastasis and non-metastasis patients, indicating PLEC might play an important role in PDAC metastasis in phosphoproteome level instead of proteome level (Additional file 7: Fig. S7E).

### The effects of diabetes on the proteogenomic characteristics of PDAC

**Fig. 4:**
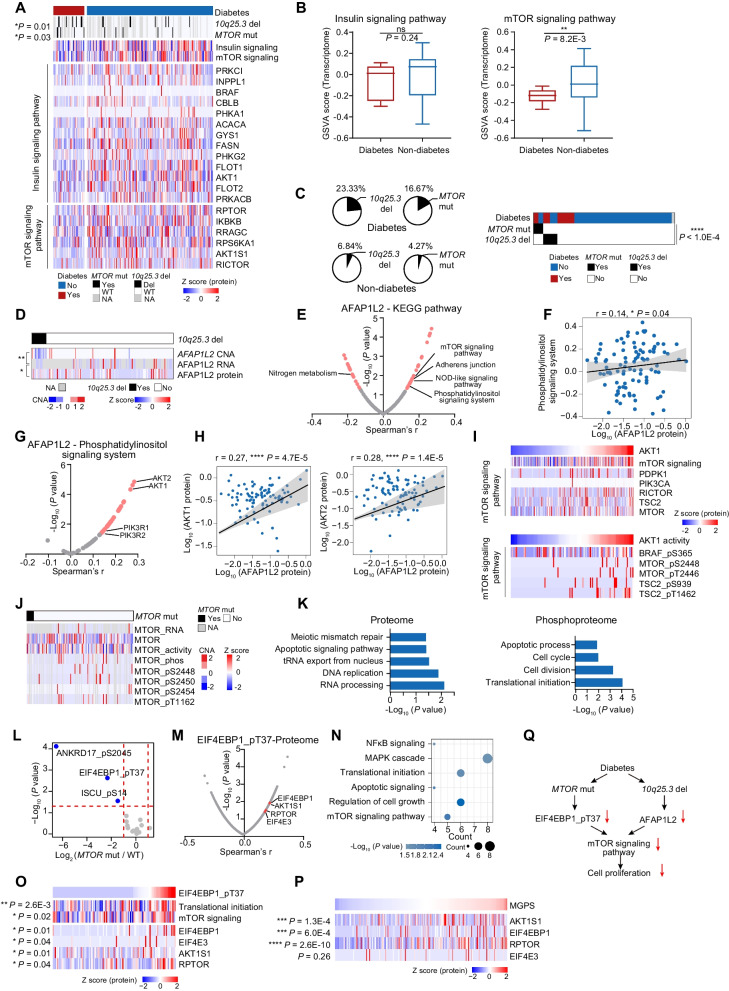
The effects of diabetes on the proteogenomic characteristics of PDAC. **A** Heatmap illustrating genomic alternations, biological pathways and protein abundance involved in these pathways of patients with and without diabetes. **B** Boxplots indicating GSVA score of insulin signaling pathway (left) and mTOR signaling pathway (right) between patients with and without diabetes (Wilcoxon test). **C** The pie charts indicating the percentage of patients with *10q25.3* deletion or *MTOR* mutations in patients with and without diabetes (left). Heatmap illustrates the association among *MTOR* mutations, *10q25.3* deletion and diabetes (Fisher’s exact test). **D** The heatmap indicating the *cis*-effect of *AFAP1L2* at chromosome locus *10q25.3* (Spearman’s correlation). **E** The scatter plot depicting the correlation between AFAP1L2 expression and KEGG pathways (GSVA score) (Spearman’s correlation). **F** Spearman-rank correlation of the abundance of AFAP1L2 and phosphatidylinositol signaling system (GSVA score). **G** The scatter plot depicting the correlation between abundance of AFAP1L2 and proteins involved in phosphatidylinositol signaling system (Spearman’s correlation). **H** Spearman-rank correlation of the abundance of AFAP1L2 and AKT1/2. **I** Heatmap indicating the impacts of AKT1 abundance on proteins involved in mTOR signaling pathway (upper). The lower heatmap indicates the impacts of activity of AKT1 on abundance of the phosphosites involved in mTOR signaling pathway. **J** Heatmap indicating the impacts of *MTOR* mutations on MTOR RNA, MTOR protein expression, kinase activity and phosphosites. **K** Bar plots indicating biological pathways downregulated in patients with *MTOR* mutations on proteomic level (left) and phosphoproteomic level (right). **L** Phosphosubstrates of MTOR differentially expressed between *MTOR*-mutant patients and *MTOR* wild-type patients. Significantly downregulated phosphosubstrates are highlighted in blue, and remaining sites are in gray. **M** The scatter plot depicting the correlation between abundance of EIF4EBP1_pT37 and all identified proteins (Spearman’s correlation). Proteins involved in mTOR signaling pathway are highlighted in red. **N** Bubble plots indicating biological pathways that EIF4EBP1_pT37 significantly positively correlated proteins enriched in. **O** The pathway (middle) and expression (down) heatmap depicting the pathways and pathway related proteins positively correlated with the EIF4EBP1_pT37 expression (Spearman’s correlation). **P** Heatmap indicating four mTOR-signaling-pathway-related proteins positively correlated with MGPS score (Spearman’s correlation). **Q** The systematic diagram summarizing the impacts of *MTOR* mutations and *10q25.3* deletion on mTOR signaling pathway and cell proliferation. ****p < 1.0E−4, ***p < 1.0E−3, **p < 1.0E−2, *p < 0.05, ns > 0.05

Long-standing diabetes is one of the major high-risk factors for PDAC [43], yet how diabetes contributes to PDAC progression and impacts patients’ prognosis is not well understood. In our cohort, patients with diabetes history accounted for 19.6% (n = 44) of 224 PDAC patients (**Methods**). We also collected patients’ glucose concentration and observed patients with and without diabetes history showed similar glucose concentration level (Additional file 8: Fig. S8A). Additionally, 25 patients have medical treatments for diabetes, while survival analysis revealed no significant difference between diabetic patients with and without medical treatment (Additional file 8: Fig. S8B). To investigate the specific molecular characteristics of the PDAC patients with diabetes, pathway enrichment analyses were performed among patients with and without history of diabetes. As a result, patients with diabetic history dominant in insulin signaling pathway and mTOR signaling pathway (Fig. 4A-B, Additional file 26: Table S4B-C).

The proteogenomic analysis identified that *MTOR* mutations were positively associated with the PDAC patients with diabetes (Fisher’s exact test, *P* = 0.03) (Fig. 4A). At the arm level, we found the deletion of *10q25.3*, identified in the malignant brain tumor [44], was the novel arm event in PDAC and positively associated with diabetes (Fisher’s exact test, *P* = 0.01) (Fig. 4A). Furthermore, in concordant with our findings, the frequencies of *MTOR* mutations and *10q25.3* deletions, were significantly higher in PDAC patients with diabetic history in both TCGA cohort [14] and WSU cohort [45] (Additional file 8: Fig. S8C-D). In addition, mutual exclusivity was observed (*P* < 1.0E−4, Fisher’s exact test) between *MTOR* mutations and *10q25.3* deletion (Fig. 4C, Additional file 26: Table S4A). Mutual exclusivity may arise when two aberrations are functionally equivalent [46]. To disclose the impacts, we then examined the changes in *MTOR* mutations and *10q25.3* deletion groups compared with the WT group.

For all protein coding genes located in *10q25.3*, *AFAP1L2*, was the only one which showed significant positive correlations (*cis*-effect) among CNA, mRNA, and protein (Fig. 4D, Additional file 8: Fig. S8E). Recent published papers have indicated AFAP1L2 was an adaptor protein involved in the PI3K-AKT pathway [47], downregulation of endogenous AFAP1L2 could inhibit AKT and destroy cell cycles [48]. Our data indicated the pathway enrichment score (GSVA score) of phosphatidylinositol signaling system (correlation = 0.14, *P* = 0.04), and mTOR signaling pathway (correlation = 0.16, *P* = 0.01) were elevated in the patients with higher AFAP1L2 protein expression (Fig. 4E, F). Further correlation analysis indicated the key molecules involved in PI3K-AKT signaling pathway, such as AKT1, AKT2, PIK3R1, and PIK3R2, showed significant positive correlations with AFAP1L2, and in which AKT1 also showed elevated expression in patients without diabetes history (Fig. 4G, H, Additional file 8: Fig. S8F). Functionally, AKT1 can serve as a hub protein to in PI3K-AKT-mTOR signaling axis [49]. Consistently, our data revealed the concordance between the enhanced AKT1 protein expression and the elevated mTOR signaling pathway, supporting by the higher pathway GSVA scores and increased protein expression of PDK1, PIK3CA, MTOR, etc. (Fig. 4I). We further inferred the kinase activity of AKT1 (Methods), and observed the abundance of phosphosites implicated in AKT-mTOR signaling pathway such as BRAF_pS365, MTOR_pS2448, TSC2_pS939, were all positively correlated with the AKT1’s kinase activity (Fig. 4I), confirming the strong association between elevated expression of AKT1 and enhanced mTOR signaling pathway.

As we mentioned earlier, *MTOR* mutations were mutually exclusive with *10q25.3* deletion, we then focused on *MTOR* mutations to explore the effect of *MTOR* mutations. Proteogenomic analysis indicated besides decreased the expression of its cognate RNA, protein and phosphosites, the *MTOR* mutations also decreased pathways such as cell proliferation, cell divisions at both proteome level and phosphoproteome level (Fig. 4J, K, Additional file 26: Table S4G). To further illustrate the impact of *MTOR* mutations on phosphoproteome, we screened out the abundance of known phosphosubstrates of MTOR, and identified the ANKRD17_pS2045, EIF4EBP1_pT37, ISCU_pS14 were significantly decreased in *MTOR*-mutant samples (Fig. 4L, Additional file 8: Fig. S8G). MTOR can drive cell proliferation and enhance RNA translational initiation though phosphorylating signaling pathway, and eukaryotic translation initiation factor 4E-binding proteins (4E-BPs) are critical mediators of mTORC1 function and can control mTORC1-mediated cell proliferation [50]. In consistent with the previous studies, our data indicated the phosphorylation of EIF4EBP1_pT37 was positively correlated with the upregulation of translational imitation, cell growth regulation and mTOR signaling pathway, supporting by the positive correlation between the abundance of EIF4EBP1_pT37 and the protein expression of EIF4E3, RPTOR, and AKT1S1 (Fig. 4M–O, Additional file 8: Fig. S8H-J). The enhanced RNA translation efficiency was reported to be associated with the elevated tumor cell proliferation [51]. Accordingly, the protein expression of EIF4E3, RPTOR, and AKT1S1 were all significantly associated with the MGPS, suggesting the elevated RNA translation efficiency led by EIF4EBP1_pT37 could promote tumor cell proliferation (Fig. 4P). In summary, these results suggest that *MTOR* mutations and *10q25.3* deletion inhibit cell proliferation, possibly through a same mechanism of modulating mTOR signaling pathway (Fig. 4Q), which provides an explanation for the mutual exclusivity between these frequent genomic aberrations in PDAC patients with the history of diabetes.

### 8p11.22 amplification associated with PDAC metastasis

**Fig. 5.**
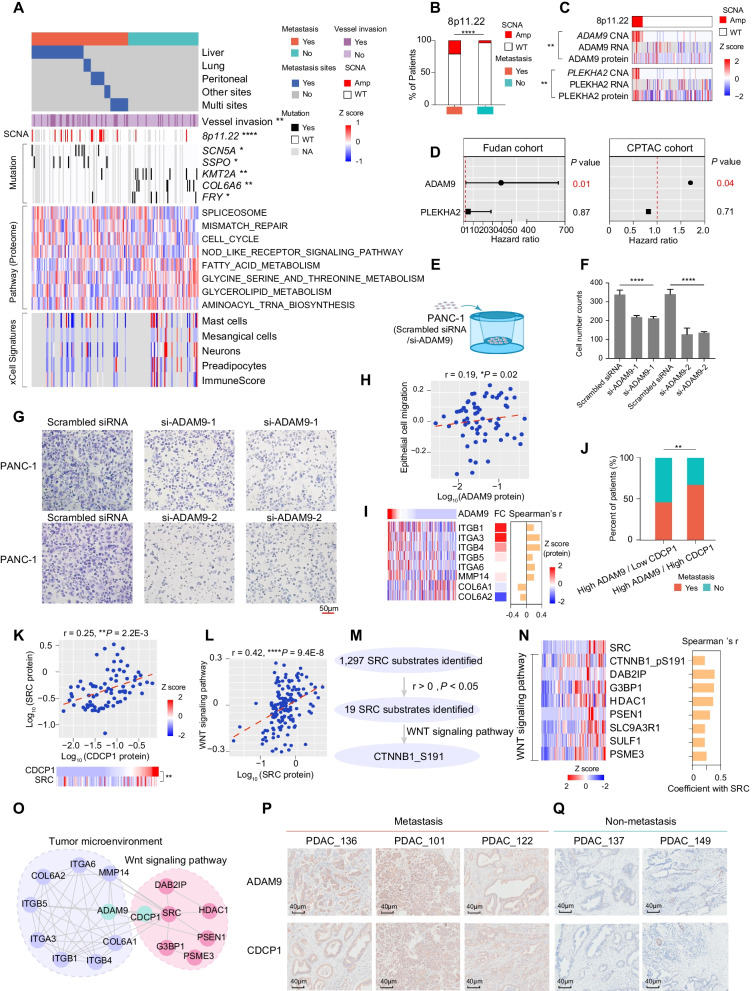
*8p11.22* amplification is associated with PDAC metastasis. **A** Heatmap illustrating the different metastatic sites, genomic alternations, biological pathways and xCell signatures of PDAC metastatic patients and non-metastatic patients (Fisher’s exact test). **B** *8p11.22* amplification in PDAC metastatic patients and non-metastatic patients (Fisher’s exact test). **C** The heatmap indicating the *cis*-effects of *ADAM9* and *PLEKHA2* at chromosome locus *8p11.22* (Spearman’s correlation). **D** The forest plot indicating the hazard ratio of ADAM9 and PLEKHA2 in Fudan cohort and CPTAC cohort. **E** The schematic work flow of transwell migration assay to evaluate the role of ADAM9 in promoting tumor cell metastasis. **F** The bar plots indicated the migrated cell counts of PANC-1 cells under different treatments. **G** Representative images of migrated PANC-1 cells. Scale bar, 50 μm. **H** Spearman-rank correlation of the abundance of ADAM9 and epithelial cell migration (GSVA score) (Spearman’s correlation). **I** Heatmap of the relative abundance of epithelial-cell-migration-related proteins that are significantly associated with ADAM9 expression. The fold change of proteins average expression between tumors and NATs and the correlation coefficient with ADAM9 are shown on the right. **J** The percent of metastatic patients and non-metastatic patients in high ADAM9/low CDCP1 and high ADAM9/high CDCP1 groups (Fisher’s exact test). **K** Spearman-rank correlation of the abundance of CDCP1 and SRC (upper). The heatmap on the bottom depicts the CDCP1 and SRC protein expression (bottom) (Spearman’s correlation). **L** Spearman-rank correlation of the abundance of SRC and WNT signaling pathway (GSVA score). **M** Flow chart for identification of SRC substrates that are significantly positively correlated with SRC expression. **N** Heatmap of the relative abundance of WNT-signaling-related proteins that are significantly associated with SRC expression (left). Spearman’s correlation is showed on the right. **O** The protein–protein interaction networks describing the interactions between ADAM9 and proteins involved in tumor microenvironment and between CDCP1 and proteins enriched in WNT signaling pathway. **P** Representative immunohistochemical staining pattern for ADAM9 and CDCP1 proteins on three tumor samples of metastatic patients. **Q** Representative immunohistochemical staining pattern for ADAM9 and CDCP1 proteins on two tumor samples of non-metastatic patients. ****p < 1.0E−4, ***p < 1.0E−3, **p < 1.0E−2, *p < 0.05, ns > 0.05

Metastasis is the leading cause of death of PDAC patients [52], a complex multi-step process involving local invasion, intravasation, extravasation, adapting to survival in new microenvironment, and finally colonize and outgrowth in distant body site [53]. However, our understanding in molecular mechanisms of PDAC has far not outpaced that in molecular traits of metastatic spread. To help development of effective treatment and improve prognosis, it is crucial to understand the molecular mechanisms of the distant metastasis of PDAC. In our cohort, 71.2% of PDAC patients (n = 161) had the metastasis/non-metastasis records, including 93 metastatic patients, and 68 non-metastatic patients. The information of PDAC remote metastasis came from 80 months of follow-up and samples used in our study were primary tumor without remote metastatic samples. We investigated the association of the genetic alterations with metastasis, and observed that the amplification of *8p11.22* (Fisher’s exact test, *P* < 1.0E−4) and mutations of *SCN5A*, *SSPO*, *KMT2A*, *COL6A6*, *FRY* showed obvious distinction in metastasis and non-metastasis patients. GSVA score showed the upregulation of spliceosome, mismatch repair, cell cycle and NOD like receptor signaling pathway and the downregulation of pathways relevant to fatty acid and glycine metabolism in the metastasis patients (Fig. 5A, Additional file 27: Table S5A-C).

Importantly, the amplification frequency of locus *8p11.22* was significantly higher in PDAC metastatic patients (Metastasis vs non-metastasis: 14 vs 2, Fisher’s exact test, *P* < 1.0E−4) (Fig. 5B). We then evaluated the *cis-*effect of genes located on this locus, and identified *ADAM9* and *PLEKHA2* showed significantly *cis*-effects on their cognate proteins (Fig. 5C, Additional file 27: Table S5D). Survival analysis further indicated the expression of ADAM9 was significantly associated with patients’ overall survival (HR = 38.9, *P* = 0.01, CI: 2.3–648.9), which was verified by the CPTAC cohort [17] (Fig. 5D). These results suggested that ADAM9 might promote PDAC metastasis. To confirm our finding, we constructed *ADAM9* knocking down PANC-1 cell lines (PANC-1-*ADAM9-*KD) (Additional file 9: Fig. S9A, Methods), and conducted transwell migration assay. As a result, transwell migration assay confirmed our findings and showed decreased cell migration ability after *ADAM9* knocked down (Fig. 5E–G).

ADAM9 is an active member of the family of transmembrane ADAMs, regulating cell behavior by binding cell-surface receptors such as integrin and membrane-type matrix metalloproteases [54]. Aiming to illustrate the mechanism underline higher ADAM9 expression in driving PDAC metastasis, we conducted correlation analysis, and found the epithelial cell migration was positively correlated with the protein expression of ADAM9 (Fig. 5H). Coordinately, epithelial cell migration-related proteins (ITGA3, ITGB1, MMP14 etc.) also showed the same expression tendency with ADAM9, and elevated expression in metastatic samples. On the contrary, collagens such as COL6A1 and COL6A2 were negatively correlated with the protein expression of ADAM9, and were decreased in metastatic samples. These results suggested the possible mechanism of ADAM9 promoted PDAC metastasis through tumor-stromal interactions (Fig. 5I). We then hypothesized that ADAM9 might enhance PDAC metastasis by upregulating epithelial cell migration and degrading collagens.

To confirm this assumption, we transfected PANC-1 cell lines with *ADAM9* siRNA and utilized scrambled siRNA as control, then conducted comparative proteomic analysis (Additional file 10: Fig. S10A). As a result, proteins that showed significantly decreased expression in *ADAM9* knocked down PANC-1 cell lines were enriched in cell migration, WNT signaling pathway, and extracellular matrix disassembly (Additional file 10: Fig. S10B). Accordingly, proteins participated in WNT signaling pathway and cell migration and angiogenesis were significantly decreased in PANC-1 cells transfected with *ADAM9* siRNAs. Whereas proteins like collagen family members were significantly elevated in PANC-1 cells transfected with *ADAM9* siRNAs (Additional file 10: Fig. S10C, Additional file 27: Table S5G). These conclusions verified our hypothesis that ADAM9 could promote tumor cell metastasis through elevated cell migration and degrading collagens.

Published papers indicated the ADAM9 can cooperate with other transmembrane proteins to promote migration. CDCP1 as a potential downstream transmembrane protein of ADAM9, was identified with the most significantly positive correlation with ADAM9 among all ADAM9-interacting proteins (Additional file 9: Fig. S9B, Additional file 27: Table S5E). Intriguingly, the percentage of metastatic patients were higher in patients with both elevated expression of ADAM9 and CDCP1 than patients with ADAM9 high expression and CDCP1 low expression (Fig. 5J). Functionally, as a glycosylated transmembrane protein, CDCP1 served as receptor, mediated multiple pathways such as WNT signaling pathway [55], cell migration [56]. To further illustrate the downstream pathways regulated by CDCP1, we conducted correlation analysis, and found the kinase–substrate pair SRC-CTNNB1_pS191. It was implicated that WNT signaling pathway was positively correlated with the protein expression of CDCP1, suggesting the possible causal link between CDCP1 and activated WNT signaling pathway in metastatic patients (Fig. 5K–M, Additional file 9: Fig. S9C). We explored the expression of the main components in WNT signaling pathway, observed proteins including JUP, GID8, CUL3, etc. showed similar expression tendency with SRC, which further confirmed CDCP1 mediated upregulation of WNT signaling pathway in promoting metastasis (Fig. 5N, O, Additional file 27: Table S5F). Immunohistochemical staining of ADAM9 and CDCP1 in the tumors of metastatic and non-metastatic patients confirmed the presence of strong signals in the tumors of metastatic patients, but not in non-metastatic patients (Fig. 5P, Q). More importantly, the significant positive correlation between ADAM9 with CDCP1, and CDCP1 with SRC were also observed in CPTAC cohort (Additional file 9: Fig. S9D), emphasized the functional link among them. Accordingly, the protein expression of ADAM9 and the GSVA score of epithelial cell migration was also observed in CPTAC cohort, which further confirmed the regulatory role of ADAM9 in promoting cell migration (Additional file 9: Fig. S9E-G).

To further confirm the causal link between ADAM9, CDCP1 and PDAC metastasis, we also performed IP-MS to identify ADAM9-interacting proteins utilizing anti-ADAM9 antibody in both PANC-1 cells with and without *ADAM9* knocked down (PANC-1-*ADAM9*-KD) (Additional file 10: Fig. S10D). Compared with PANC-1 treated with scrambled siRNA, 39 proteins that specifically interacted with ADAM9 were identified in PANC-1-*ADAM9*-KD group (Additional file 27: Table S5H). GO enrichment revealed the main biological pathways that most significantly enriched by ADAM9 interacted proteins were WNT signaling pathway, TGFβ signaling pathway and degradation of ECM (Additional file 10: Fig. S10E). Importantly, among these proteins, CDCP1 showed strongest interaction with ADAM9 (Additional file 10: Fig. S10F), which further confirmed the causal link among ADAM9, CDCP1 and PDAC metastasis.

Generally, *8p11.22* amplification promoted metastasis by upregulating ADAM9 expression and activated WNT signaling pathway. *8p11.22* amplification might be a biomarker for PDAC to predict metastasis.

To gain greater insight into the relationship between molecular features and different metastatic sites of PDAC, we further accessed the differential multi-omics features of the primary tumors, which metastasize to the different sites after the operation. According to the clinical record in our cohort, we grouped the metastasis patients into liver-metastasis (n = 50), lung-metastasis (n = 7), peritoneal-metastasis (n = 13), other-metastasis (n = 6), and multi-sites-metastasis (n = 17). We compared ADAM9 protein expression between patients with different metastatic sites and found no significant differences (Additional file 9: Fig. S9J), implied that the expression of ADAM9 might have no significant impact on the metastatic sites. Next, we performed weighted gene correlation network analysis (WGCNA) and found obvious association between the proteomic modules and the metastasis sites. The correlations between MEbrown and liver, MEmagenta and lung, MEpurple and peritoneal, MEred and other sites, and MEcyan or MEtan and multi-sites are shown in Additional file 9: Fig. S9H (Additional file 27: Table S5I-J). These modules with the metastatic site-specific enrichment showed that liver was featured by oxidative phosphorylation (including ATP5D, NDUFB3, NDUFV3, and CYC1) and pancreatic secretion (including CPB1, CEL, CELA3A, and PRSS1), lung was featured by immune relative pathway such as leukocyte transendothelial migration (including CST3, DSP, CTNNA1, and STAT3), peritoneal was characterized by ECM relative pathway such as focal adhesion (including PKP3, MYL6, CAV1, and PPP1R12C). The histone family proteins were overrepresented in the multi-site-metastasis group, such as HIST1H1B, HIST1H1C, and HIST1H1D (Additional file 9: Fig. S9I). Altogether, site-specific features at a multi-omics level provided a clue of the potential therapy strategy for PDAC patients.

### Proteomic subtypes of PDAC patients

**Fig. 6:**
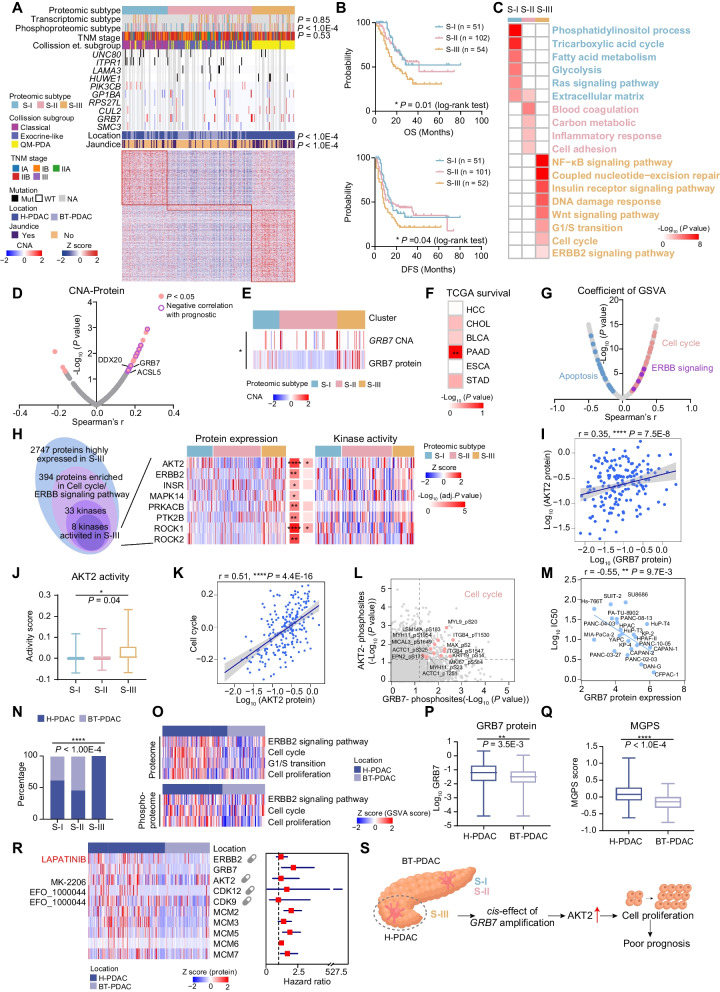
Multi-omics subtypes of PDAC patients. **A** Consensus-clustering analysis of proteomic profiling identifying three proteomic subtypes: S-I (blue, n = 57), S-II (pink, n = 106) and S-III (yellow, n = 54). The associations of proteomic subtypes with mutation information and clinical characteristics are annotated in the middle panel. The heatmap depicts the relative abundance of signature proteins (bottom). **B** The association of three proteomic subtypes with clinical outcomes in 217 PDAC samples (log-rank test). OS, overall survival; DFS, disease free survival. **C** Gene Ontology (GO) terms enriched in the three proteomic subtypes. **D**, **E** Spearman-rank correlation of CNA and protein that highly expressed in S-III. The significant correlations are colored in pink, and prognostic related proteins are labeled with purple circles (D). The *cis*-effect of *GRB7* among CNA and protein is shown in (E). **F** *P* values for the correlation between IRF6 protein expression and prognosis in six kinds of gastrointestinal tumors in TCGA data. **G** Spearman-rank correlation of the abundance of GRB7 protein and GSVA score. Pink, cell cycle; purple, ERBB signaling pathway; blue, apoptosis. **H** The Venn plot on the left depicting the activated kinases in S-III and heatmaps on the right showing the global abundance and the kinase activity score of these selected kinases. For each kinase, the *P* value of its global abundance and kinase activity among three subtypes are shown in the center panel. **I** Spearman-rank correlation of the abundance of GRB7 and AKT2. **J** The boxplot indicating the comparison of kinase activity of AKT2 among three subtypes (one-way ANOVA). **K** Spearman-rank correlation of the abundance of AKT2 and GSVA score of cell cycle. **L** Scatter plot displaying the association between each phosphosites’ abundance with the global abundance of AKT2 (y axis) versus the global abundance of GRB7 (x axis). Cell cycle-associated phosphosites are labeled in pink. **M** The scatter plot indicating the cell lines with higher level of GRB7 protein expressions are more sensitive to ERBB2 inhibitor treatment (lower IC50) (Spearman’s correlation). **N** The bar plot indicating the frequency of pancreatic head (H-PDAC) and pancreatic body and tail (BT-PDAC) among three subtypes (Fisher’s exact test). **O** The heatmap indicating the GSVA scores of the pathway between H-PDAC and BT-PDAC in proteome and phosphoproteome levels. Each column represented a patient sample, color of each cell showed GSVA scores. **P**, **Q** The boxplot indicating the comparison of abundance of GRB7 (P) and MGPS (Q) score between H-PDAC and BT-PDAC (Student’s t test). **R** The heatmap indicating the cascading expression patterns of ERBB2, GRB7, and cell cycle related genes between tumor locations. The hazard ratios of these proteins are showed on the right. **S** Illustration of the regulatory role of *GRB7* amplification. ****p < 1.0E−4, ***p < 1.0E−3, **p < 1.0E−2, *p < 0.05, ns > 0.05

As the current radiologic or pathologic staging system used for PDAC could not precisely predict prognosis or provide indications for effective treatment, we employed a consensus clustering [57] based on proteins expression ranks in the tumor samples, and identified three subgroups among the 217 PDAC tumors (Fig. 6A, Additional file 11: Fig. S11A-B, Additional file 28: Table S6A-B, Methods).

Combined with clinical data, we found the proteomic subgroups significantly differed in overall survival (OS; log-rank test, *P* = 0.01) and disease free survival (DFS; log-rank test, *P* = 0.04, Fig. 6B). Evaluation of the clinical features of the proteomic subgroups revealed that the S-III subgroup, which had a poorest overall survival, was characterized with 100 percentage of pancreaticoduodenal patients, and comparatively high percentage of patients with jaundice (46.3%) (Fig. 6A). Additionally, we observed no significantly difference in tumor purity among proteomic subgroups (Additional file 15: Fig. S15A). GO enrichment analysis revealed that S-I (denoted by glycolysis and TCA cycle) was associated with metabolic bioprocesses including tricarboxylic acid cycle (TCA), fatty acid metabolism, and glycolysis; S-II (denoted by blood coagulation) was associated with blood coagulation; whereas S-III (denoted as cell cycle) was featured with ERBB2 signaling pathway, DNA damage response, and cell cycle (Fig. 6C, Additional file 28: Table S6C). Importantly, glycolysis-related proteins PFKL and MDH2 enriched in S-I (ANOVA, Benjamini–Hochberg adjusted p < 0.05), blood coagulation-related proteins FGG and GP1BA enriched in S-II (ANOVA, *P* < 0.05) were positively associated with prognosis (log-rank test, Benjamini–Hochberg adjusted p < 0.05) (Additional file 11: Fig. S11D). In contrast, MCM2 and NCF1 were highly expressed in the S-III, and were negatively associated with prognosis (log-rank test, Benjamini–Hochberg adjusted p < 0.05) (Additional file 11: Fig. S11D).

We further observed the distinctive molecular features of three proteomic subgroups at transcriptomic and proteomic level. Particularly, 754 genes, 233 genes and 350 genes showed elevated expression at both mRNA and protein level in S-I, S-II, and S-III, respectively. For instance, the expression of some reported molecular such as, APOA1, S100A4, and C19orf33, showed consistent expression tendency at both proteomic and transcriptomic level [17, 58, 59] (Additional file 12: Fig. S12A-C). Noticeably, although both proteomic and transcriptomic data could reveal the specific molecular features of distinctive subgroup, some alterations of druggable targets were only observed at proteomic level. For instance, the key molecules of ErbB_EGFR signaling pathway (ERBB2, MAP2K7, etc.), only showed significantly elevated expression among three subtypes at proteomic level (Additional file 12: Fig. S12D). These findings implied that the proteomic data provided an opportunity for further exploration of the mechanism of PDAC.

In addition, with the tremendous appeal of kinases as drug targets, it is of great importance to characterize the distinctive kinase phosphosubstrate among the three proteomic subtypes. The phosphoproteomic analysis of kinase revealed the obvious difference in three proteomic subtypes (Additional file 12: Fig. S12E-F). Further integrative analysis among phosphosubstrates and kinases revealed the similar prognostic tendency between kinase and their phosphosubstrate, such as the dominant kinase PRKCB and its substrate HMGA1_pT42 in S-I, and kinase MTOR and its substrate SRRM2_pS1329 in S-III (log-rank test, *P* < 0.05) (Additional file 12: Fig. S12G). These results implied that patients belonging to different proteome subtypes had different kinase profiles and might be suitable for different therapeutic strategies.

To further assess the intersection of our proteomic subtypes with TNM stage, we compared subtypes assignment of 217 patients using each of the two classifiers. As a result, there was no significant correlation between proteomic subtype and TNM stage distribution (Fig. 6A). Importantly, the early-stage (IA and IB) PDAC was orthogonally distributed across the three proteomic subtypes, suggesting that this class was not restricted to a specific proteomic feature (Additional file 13: Fig. S13A). Further survival analysis revealed that our proteomic subtyping could capture differences in survival in the early-stage PDACs, suggested that our proteomic subtype might indicate the prognosis for patients with early-stage PDAC (Additional file 13: Fig. S13B). Pathway enrichment analysis clearly demonstrated similar molecular features among the three proteomic subgroups in early-stage PDAC patients (Additional file 13: Fig. S13C). Similarly, differentially expressed kinase signatures among the three proteomic subtypes were also present in patients with early-stage PDAC (Additional file 13: Fig. S13D). Furthermore, univariate analysis showed that our proteomic subtype might be an independent prognostic indicator for PDAC patients (Table 2).

**Table 2:** Univariate analysis of overall survival in PDAC patients

	No. of patients	HR (95% CI)	*P* value
Age	226	0.98 (0.96–1.01)	0.189
History of diabetes		1.33 (0.75–2.33)	0.327
Yes	44		
No	183		
Not sure	2		
TNM stage*		1.2 (1–1.45)	0.046
IA	28		
IB	77		
IIA	16		
IIB	89		
III	19		
ECM subtype		1.33 (0.97–1.82)	0.076
Deserted	48		
Inter	77		
Reactive	85		
Proteomic subtype*		1.5 (1.09–2.06)	0.012
S-I	57		
S-II	106		
S-III	54		
Immune subtype**		0.38 (0.2–0.7)	0.002
Im-S-I	9		
Im-S-II	15		
Im-S-III	9		
Im-S-IV	8		
Im-S-V	13		

We also conducted clustering analyses on the tumor transcriptome (n = 54, consensus clustering) and phosphoproteome (n = 113, consensus clustering), and identified three subtypes in each dataset (Additional file 11: Fig. S11A). Generally, a moderate concordance among the transcriptomic, proteomic, and phosphoproteomic subtypes was revealed (45% between proteomic and phosphoproteomic subtypes and 39% between proteomic and transcriptomic subtypes). There was no obvious difference of the proportion of transcriptomic subtypes among three proteomic subtypes (*P* = 0.85) (Additional file 11: Fig. S11C).

Next, we applied the Collisson’s transcriptomics-based subtyping strategies for PDAC to the entire set of tumors to explore inter-sample heterogeneity [60]. As a result, the significant concordance was observed between our proteome-based subtypes and Collisson’s subtypes (Additional file 11: Fig. S11E). For instance, our S-I subgroup overlapped with “classic” (80.7% overlap), our S-II subgroup overlapped with “exocrine-like” (62.3% overlap) and our S-III subgroup mainly overlapped with “QM-PDA” (98.1% overlap). Notably, even though the majority of the “QM-PDA” tumors were classified into S-III subgroup, 21 “QM-PDA” tumors were classified into our S-II subgroup (Fig. 6A). Interestingly, splitting “QM-PDA” tumors according to the proteome-based clusters revealed a trend of distinct prognostic outcomes (Additional file 11: Fig. S11F). Concordantly, we observed that for 217 PDAC samples with both proteome-based and Collisson’s assignments, the proteome-based subtypes showed stronger prognostic separation than Collisson-dichotomized subtypes (log-rank test, *P* = 0.01 versus *P* = 0.19). We further performed comparative analysis between the proteome-based S-II and S-III “QM-PDA” tumors. As a result, the S-III “QM-PDA” tumors presented an aggressive characteristic. Proteins elevated in this S-III “QM-PDA” subgroup were dominant in mRNA splicing and cell proliferation process (Additional file 11: Fig. S11G-H). Together, these results supported the association of proteome-based subtyping with patient outcome. Further experimental investigation of the over-activated proliferative and signaling pathways in the poor prognosis proteome-based S-III might facilitate the development of subtype-specific therapeutic strategies.

In addition, we also performed consensus clustering for 137 CPTAC tumor samples in the same way as we used in our study, and stratified three proteomic subgroups with significant prognostic values [17] (Additional file 14: Fig. S14A-E and J). Subgroup-specific pathway enrichment analysis clearly demonstrated similar molecular features among the three proteomic subgroups (S-I: metabolic bioprocesses; S-II: blood coagulation; S-III: cell cycle) in CPTAC cohort and Fudan cohort (Additional file 14: Fig. S14D-F). These results supported the reliable subgrouping procedure in our study. In addition, we conducted an integrated analysis of characteristic proteins of three subgroups and the clinical information to explore the association of our proteomic subtypes with patients’ outcome. Three characteristic proteins (DPT, NAMPT, and MCM7) which significantly associated with prognosis were identified in both Fudan and CPTAC cohort (Additional file 14: Fig. S14I), revealing the prognostic relevance of proteomic subtypes, further highlighting the clinical implications of our proteomic subtypes.

In order to find proteins that could be used to verify each proteomic subtypes by immunohistochemistry, we set the following criteria to screen discriminative markers for PDAC proteomic subtypes: (1) expressed in at least 90% of samples; (2) expression of protein in S-I, S-II or S-III was significantly higher than the other two subtypes (ratio > 1.5, Benjamini–Hochberg adjusted p < 0.05); (3) related to prognosis. We finally identified 34 proteins biomarkers (ACOX1, LTF and MCM2 etc.) that showed dominant expression in a specific proteomic subgroup and were functionally relevant to the main function of the distinctive subgroup (Additional file 15: Fig. S15B-C). We then randomly selected 3 protein marker candidates (S-I: ACOX1; S-II: S100A4; S-III: MCM6) and validated their expression in specific proteome-subgroup by immunohistochemistry (IHC) (Additional file 15: Fig. S15D). These suggested that the panel of biomarker candidates could be potential candidates used to distinguish different subtypes, implying the possibility to directly translate our findings into laboratory tests.

### GRB7 amplification was enriched in S-III subgroup

To explore the mechanism of different proteomic subtypes, we focused on the changes on genome level. We observed the subtype-specific mutations: S-I had the highest mutational frequency of *UNC80* (17.1%, Fisher’s exact test) and *ITPR1* (17.1%), which were involved in transport of glucose; *LAMA3* mutations specifically occurred in the S-II (7.9%); whereas S-III had the highest mutational frequency of *HUWE1* (10.8%), which was involved in DNA damage response and VEGF receptor signaling [61] (Fig. 6A). We also observed the subtype-specific SCNA: *PIK3CB* (*3q22.3*, 11.4%, Fisher’s exact test) was higher amplificated in S-I; the amplification of platelet glycoprotein Ibα*(GP1BA*) specifically occurred in the S-II (*17p13.2*, 5.2%); whereas S-III had the highest amplification of *GRB7*, which were involved in cell cycle process (*17q12*, 16.2%) [62] (Fig. 6A).

Noticeably, S-III subgroup showed poor prognosis was characterized with higher amplification frequency of *GRB7* (location:17q12, S-I:8.6%, S-II:10.4%, S-III:16.2%), at genomic level. Gene ontology analysis showed the enhanced enrichment of ERBB2 signaling pathway, and cell cycle process at proteomic level (Fig. 6C). *GRB7* is an oncogene, participating in various signaling pathways implicated in cell migration, metastatic invasion, cell proliferation and tumor-associated angiogenesis [62]. Combined with proteomic data, we observed the *cis*-effect of *GRB7* on its cognate protein’s expression (Fig. 6D, E). Besides, survival analysis revealed that the higher protein expression of GRB7 was associated with poor prognosis. This observation was further supported by TCGA data. Intriguingly, we found among the 6 digestive systems tumor types, GRB7 was most significantly associated with prognosis in PDAC, further emphasized the clinical importance of GRB7 in PDAC (Fig. 6F).

Previous studies have indicated that upregulated GRB7 could promote proliferation and tumorigenesis via AKT pathway and could be affected by ERBB [63, 64]. Consistent with previous studies, our data showed strong association between the protein expression of GRB7 and the GSVA score of ERBB signaling pathway and cell cycle process, suggested the regulatory role of GRB7 in leading to poor prognosis through upregulating ERBB-cell cycle process (Fig. 6G). To decipher this hypothesis, we analyzed the 394 S-III highly expressed ERBB-cell cycle-related proteins, and found 8 kinases (AKT1, ERBB2, ROCK1, etc.) were activated in S-III, in which the top-ranked kinase AKT2 was positively correlated with the protein expression of GRB7 (Fig. 6H–J; Additional file 28: Table S6D). Consistently, the protein expression of AKT2 was also positively correlated with the cell cycle process (Fig. 6K). In addition, GRB7, ERBB2, and AKT2 were also highly expressed in S-III which were characterized by cell cycle in CPTAC cohort [17] (Additional file 14: Fig. S14G-H). These results proposed the possible causal link between GRB7 and cell cycle process, mediated by AKT2. We then employed a network-based approach to study the effect of AKT2 under *GRB7* amplification on the phosphoproteome. The elevated abundance of phosphosites enriched in cell cycle, such as MYL9_pS20, ITGB4_pT1530, and LSM14A_pS183, were significantly associated with the high expression of both *GRB7* and AKT2 (Fig. 6L). Moreover, examining published cell line perturbation experiments from GDSC, we found the cell lines with higher GRB7 expression were more sensitive to LAPATINIB (ERBB inhibitor) (Fig. 6M). These results suggested regulatory role of AKT2 in promoting ERBB-cell cycle process in *GRB7* amplificated tumors, and provided a possible therapeutical option of inhibiting ERBB2 in *GRB7* amplification PDAC patients.

### ERBB2-cell cycle signaling axis promoted cell proliferation of H-PDAC patients

According to previous researches, tumor location strongly associated with the prognosis of PDAC [65]. Patients with tumor located on head (H-PDAC) showed poor prognosis comparing to the patients with tumor located on body-tail (BT-PDAC) in both Fudan and CPTAC cohort [17] (Additional file 11: Fig. S11I). Interestingly, the GSVA score of ERBB2 signaling pathway and cell cycle were higher in H-PDAC both in protein and phosphoprotein level, and similar results were verified in the CPTAC cohort (Fig. 6O, Additional file 11: Fig. S11J, Additional file 28: Table S6E). Furthermore, we observed comparing to the S-I and S-II, tumors of S-III patients (shortest overall survival) were all H-PDAC (Fig. 6N).

To illustrate the molecular features which contributed to the poor prognosis of H-PDAC, comparative analysis was performed. As a result, the pathways overrepresented in H-PDCA tumors were also ERBB2-cell cycle signaling axis (Student’s t test, *P* < 0.05), supporting by the elevated protein expression of GRB7, ERBB2, AKT2, MCM2/3/5/6/7 (Student’s t test, *P* < 0.05) and higher MGPS in H-PDAC (Fig. 6P–R, Additional file 28: Table S6F). Further survival analysis revealed that these proteins implicated in GRBP7-ERBB2-AKT2-cell cycle signaling axis were related to the poor prognosis.

In sum, these findings portrayed an GRBP7-ERBB2-AKT2-cell cycle signaling axis promoted enhanced proliferation feature of H-PDAC, and provided possible therapeutical target for H-PDAC (LAPATINIB: ERBB2, MK-2206: AKT2, EFO_1000044: CDK12/9) (Fig. 6S).

### Characterization of immune infiltration in PDAC

**Fig. 7:**
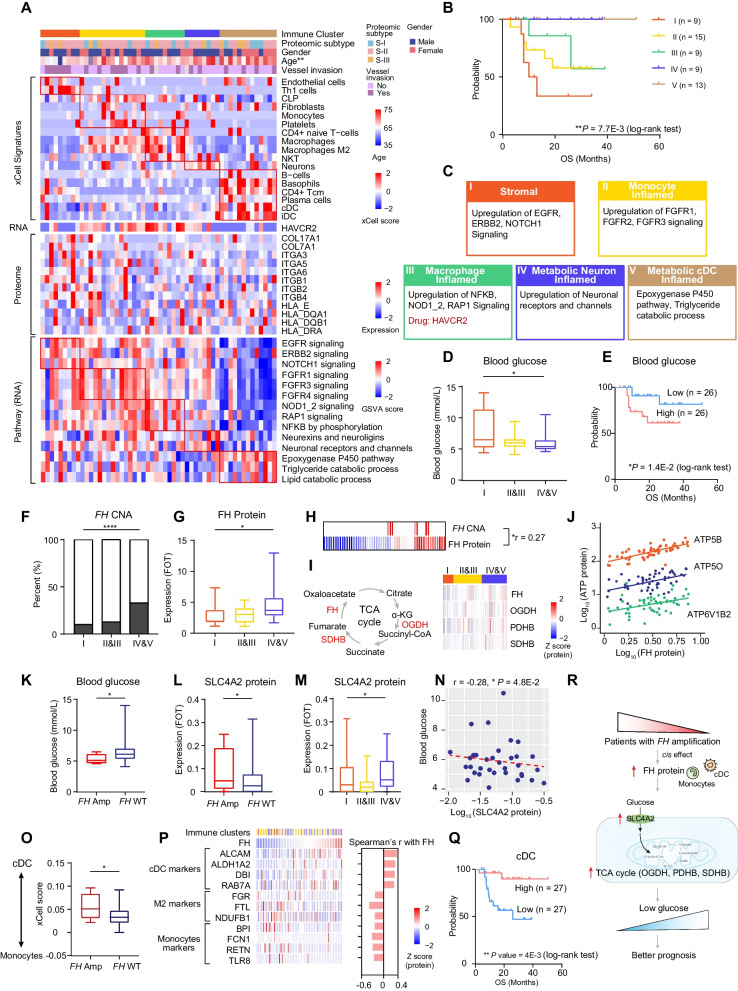
Characterization of Immune Infiltration in PDAC. **A** Heatmap illustrating cell type compositions and activities of selected individual gene/proteins and pathways across 5 immune clusters. The heatmap in the first section illustrates the immune/stromal signatures from xCell. RNA and protein abundance of immune-related markers and GSVA scores based on mRNA data for biological pathways upregulated in different immune groups are illustrated in the remaining sections. **B** Kaplan–Meier curves for overall survival based on immune clusters (log-rank test). **C** Key upregulated pathways based on RNA-seq for each immune cluster showing in the annotation boxes. **D** Boxplot illustrating blood glucose among Im-I, Im-II&III and Im-IV&V (one-way ANOVA). **E** Kaplan–Meier curves for overall survival based on blood glucose high patients (n = 26) and blood glucose low patients (n = 26) (log-rank test) involved in immune clusters. **F** *FH* amplification status in 3 immune clusters (Fisher’s exact test). **G** Boxplot illustrating FH protein expression among Im-I, Im-II&III and Im-IV&V (one-way ANOVA). **H** The heatmap indicating the *cis*-effect of *FH* amplification (Spearman’s correlation). **I** Heatmap illustrating 3 key enzymes’ expression among Im-I, Im-II&III and Im-IV&V (right). These key enzymes are involved in TCA cycle (left). **J** Spearman-rank correlation of the abundance of 3 ATP synthases and FH (Spearman’s correlation). **K** Boxplot indicating blood glucose of patients with *FH* amplification and *FH* wild type (Wilcoxon test). **L**, **M** Boxplots indicating glucose transporter SLC4A2 protein expression of patients with *FH* amplification and *FH* wild type (L) (t test) and among 3 immune clusters (M) (one-way ANOVA). **N** Spearman-rank correlation of the abundance of SLC4A2 and blood glucose (Spearman’s correlation). **O** Distribution of monocytes and cDC polarization in PDAC with different *FH* statuses (Wilcoxon test). **P** Heatmap illustrating cDC, macrophage M2 and monocytes markers expression (left) and their correlation with FH abundance (right). **Q** Kaplan–Meier curves for overall survival based on cDC score high patients (n = 27) and cDC score low patients (n = 27) (log-rank test) involved in immune clusters. **R** The systematic diagram summarizing the impact of the amplification of *FH* on blood glucose and tumor microenvironment. ****p < 1.0E−4, ***p < 1.0E−3, **p < 1.0E−2, *p < 0.05, ns > 0.05

Although immunotherapy has been used as a novel treatment for PDAC, its efficacy differs among patients. To better understand the features of immune infiltration in PDAC, we performed xCell (https://xcell.ucsf.edu) (Methods), based on the transcriptomic data to infer relative abundance of different cell types in the tumor microenvironment (Fig. 7A, Additional file 29: Table S7A-C). Consensus clustering based on inferred cell proportion helped identify the following five subgroups of tumors with distinct immune and stromal features: Im-S-I (Stromal: n = 9), Im-S-II (Monocyte-Inflamed: n = 15), Im-S-III (Macrophage-Inflamed: n = 9), Im-S-IV (Metabolic-Neuron-Inflamed: n = 9) and Im-S-V (Metabolic-cDC-Inflamed: n = 13). Survival analysis indicated the immune subgroups significantly differed in overall survival (OS; log-rank test, *P* = 7.7E−3), suggesting that different type of immune cell infiltration could lead to diverse prognostic outcomes (Fig. 7B).

The stromal subgroup, containing relatively young patients, was characterized by endothelial cells and elevated expression of stromal related proteins (COL17A1, COL7A1, ITGA3, etc.). Pathway analysis indicated that the stromal group showed upregulation of EGFR signaling pathway, ERBB2 signaling pathway. The Monocyte-Inflamed subgroup was characterized by high level of monocytes-infiltration and low T cell infiltration. Pathway analysis indicated that this subgroup showed upregulation of FGFRs signaling pathway. The Macrophage-Inflamed subgroup, showed infiltration of tumor-associated macrophage (TAM) and increased expression of the immune evasion marker HAVCR2 (TIM-3). In concordant with previous studies which emphasized the TAM infiltration is associated with a poor prognosis [66], this subgroup was also showed comparatively poor prognosis (Fig. 7B). Pathway enrichment revealed significant enrichment of NF-κB signaling pathway which implicated in the macrophage activation. The Metabolic-Neuron-Inflamed subgroup was characterized by neurons. Pathway analysis indicated that this subgroup showed upregulation of neuronal receptors and channels. The Metabolic-cDC-Inflamed group was characterized by cDC, CD4^+^ Tcm and B cells, etc. Pathway analysis indicated that this group showed upregulation of triglyceride and lipid catabolic process. Accordingly, the antigen presentation MHC molecules: HLA-E, HLA-DQA1, HLA-DQB1, HLA-DRA, were enhanced in this subgroup (Fig. 7C).

In order to explore the correlation of immune subtype with age, we compared age among 5 immune subtypes. The age of patients in Im-S-I, Im-S-II and Im-S-III were younger than patients in Im-S-IV and Im-S-V (Additional file 16: Fig. S16A). These results suggested that younger PDAC patients might have less immune infiltration compared to older PDAC patients. To confirm this observation, we referred clinical and transcriptomic data from TCGA-PDAC cohort [67], performed xCell deconvolution analysis based on transcriptomic data, and dichotomized patients into younger and older groups based on age (younger group: median = 58 versus older group: median = 66). By comparing the xCell algorism-based cell deconvolution results among the two groups, we found the patients belong to the younger group showed lower immune scores, suggested less immune cell infiltration (Additional file 16: Fig. S16B). On the contrary, patients in older group were featured with elevated immune cell infiltrations. Enrichment score of the cells, such as pro B cells, pDC and CD8^+^ Tem, were higher in older group than younger group (Additional file 16: Fig. S16B). These results confirmed our finding that older patients had more immune cell infiltration than younger patients, implying the immune therapy might be more likely to benefit older patients.

To assess the intersection of our immune subtypes with proteomic subtypes, we compared subtypes assignment of PDAC patients using each of the two classifiers. As a result, the S-II subtype was orthogonally distributed across the five immune subtypes, suggesting the proteomic subtypes was not restricted to a specific immune feature (Additional file 16: Fig. S16C-D). Further survival analysis revealed that our proteomic subtyping could capture differences in survival in S-II subtype (Additional file 16: Fig. S16E). We then conducted comparative analysis and found the immune cell infiltration characteristics of the five immune subtypes in S-II were also Th1 cells (Im-S-I), fibroblasts, monocytes (Im-S-II), macrophages, macrophages M2 (Im-S-III), neurons (Im-S-IV) and basophils, CD4 + Tcm, cDC, iDC (Im-S-V) (Additional file 16: Fig. S16F).

### The role of FH in affecting tumor progression and microenvironment in PDAC

The immune subtypes were annotated based on their gene expression profiles and immune cells infiltration. Im-S-II and Im-S-III had similar expression signature, and Im-S-IV and Im-S-V had similar expression signature (Additional file 16: Fig. S16G-H). The prognosis was similar for Im-S-II and Im-S-III and similar for Im-S-IV and Im-S-V. Based on the above observation, we merged the 5 subtypes into 3 subtypes (Im-1: Im-S-I; Im-2: Im-S-II and Im-S-III; Im-3: Im-S-IV and Im-S-V) (Methods). Intriguingly, combined with clinical features, we found the blood glucose were comparatively lower in Im-3 (featured with metabolism). Previous studies have indicated that the alteration of blood glucose levels greatly affected tumor microenvironment and patients’ prognosis [68]. Consistently, our data indicated blood glucose was negatively associated with overall survival (Fig. 7D, E).

To reveal the possible mechanisms underlined the blood glucose difference among the three subgroups, we first compared the genomic events among them, and observed the amplification frequency of *FH* was elevated in Im-3 (Fig. 7F, Additional file 29: Table S7D). Proteogenomic analysis revealed a *cis*-effect of *FH* on its cognate protein (Fig. 7G, H). In concordant with our result, the association between the protein expression of *FH* and its copy number were also observed in TCGA [14] and WSU [45] cohort (Additional file 17: Fig. S17A). Intriguingly, the frequencies of *FH* amplification showed no difference between patients with and without diabetic history (Additional file 17: Fig. S17B). The same phenomenon was also observed in both TCGA and WSU cohort (Additional file 17: Fig. S17C). Fumarate hydratase (FH) is an enzyme of the tricarboxylic acid cycle (TCA cycle) that catalyzes the hydration of fumarate into malate [69]. Therefore, we surveyed the TCA related proteins’ expression and observed the expression of several key enzymes in TCA cycle, including 2-oxoglutarate dehydrogenase (OGDH), Pyruvate dehydrogenase beta subunit (PDHB) and succinate dehydrogenase B (SDHB) were upregulated in Im-3 subgroup (Fig. 7I, Additional file 29: Table S7E). Moreover, three ATP synthase: ATP5B, ATP5O and ATP6V1B2 were also positively correlated with FH expression and elevated in Im-3, further confirming the upregulation of TCA metabolism and ATP synthesis in Im-3 (Fig. 7J). We then hypothesized that the amplification of *FH* might decrease patients’ blood glucose through elevated TCA cycle. To confirm this assumption, we first compared the blood glucose between patients with and without *FH* amplification. As expected, patients with *FH* amplification showed lower blood glucose (Fig. 7K).

We then utilized siRNA to knock down the expression of FH in two PDAC cell lines (SW1990 and PANC-1), and further examined tumor cell proliferation, detected the tumor cell glycolytic rates as well as evaluated the concentration of core metabolites of glucose metabolism. The experiments were performed under both high glucose and low glucose conditions (Additional file 17: Fig. S17D). As a result, comparing to cell lines transfected with scrambled siRNA, cell lines that knock down *FH* showed elevated cell proliferation rates in both SW1990 and PANC-1, under both high and low glucose conditions (Additional file 17: Fig. S17E). Meanwhile, we found that the cell lines with *FH* knock down showed significantly higher tumor cell proliferation rates under high glucose condition, comparing to low glucose condition. In contrast, the cell lines transfected with scrambled siRNA showed no difference in tumor cell proliferation rates between high glucose and low glucose conditions (Additional file 17: Fig. S17E). These results emphasized the loss of *FH* could promote tumor cell proliferation, which could be further enhanced under high glucose condition.

To illustrate whether lower FH expression promote tumor cell proliferation through regulating the glucose metabolism, we detected glycolytic rate, glucose consumption, oxaloacetate, and lactate production. As a result, in line with the cell proliferation patterns, the *FH* knock down cell lines exhibited decreased glycolytic rate, lower glucose consumption, decreased oxaloacetate production and increased lactate production under both high glucose and low glucose conditions (Additional file 17: Fig. S17F). These results demonstrated that *FH* knockdown might altered glucose metabolism into a more efficient way and led to increase tumor cell proliferation, specifically under high glucose condition.

Since glucose transporters play an essential role in regulating both glucose concentration and TCA cycle activity [70]. We then surveyed the protein expression of GLUT family, and identified GLUT4 showed upregulated protein expression in patients with *FH* amplification (Fig. 7L, M). Moreover, the protein expression of GLUT4 was also negatively correlated with blood glucose (Fig. 7N). Therefore, our results indicated the *FH* amplification elevated its cognate protein’s expression, increased the TCA cycle, ATP synthesis, and increased the glucose transport, which led to decrease the patients’ blood glucose.

Multiple researches have emphasized the close relationship between the metabolism status of cancer cell and immune infiltration. Interestingly, we observed significant difference between M2 macrophage and cDC across *FH* statuses. To be more specific, the amplification of *FH* promoted more cDC infiltration, and *FH* wild type had more M2 macrophage infiltration, which was supported by the positive correlation between FH and cDC signature proteins (ALCAM, ALDH1A2, DBI, RAB7A) and the negative correlation between FH and M2 macrophage markers (FGR, FTL, NDUFB1) (Fig. 7P). Coordinately, the cDC polarization score was higher in *FH* amplification group (Fig. 7O). Moreover, the protein expression of cDC markers including ALCAM, ALDH1A2, DBI, were positively correlated with the protein expression of FH, whereas the protein expression of M2 markers, and monocyte markers (such as BPI, FCN1, RETN) were negatively correlated with the protein expression of FH, further confirming the FH promoted the monocyte to cDC polarization (Fig. 7P). Survival analysis indicated higher cDC infiltration led to better prognosis (Fig. 7Q). More importantly, to further confirm the potential role of FH in altering monocytes and cDC infiltration in PDAC microenvironment, we collected cultivate supernatant of both *FH* knock down cell lines and scrambled siRNA treated cell lines, which were cultivated in either high glucose or low glucose conditions, then performed comparative proteomic analysis (Additional file 17: Fig. S17G). As a result, cytokines including CCL2, CCL3, CCL5 and CCL7 which participated in monocytes recruitment were significantly higher in cultivate supernatant of *FH* knock down cell lines, specifically under high glucose condition (Additional file 17: Fig. S17H). On the contrary, the cytokines that promoted the transformation from monocytes to cDCs such as IL-1, IL-12, IL-23 and TNF-α, were significantly decreased in cultivate supernatant of *FH* knock down cell lines, specifically under high glucose condition (Additional file 17: Fig. S17H). These results demonstrated the impact of FH on the monocytes’ transformation to cDC cells and also emphasized that high glucose condition could further enhanced this affect. In sum, PDAC patients with *FH* amplification might mean more favorable prognosis because of lower blood glucose and more cDC infiltration in tumor microenvironment (Fig. 7R). It also reminded us that maintaining blood glucose levels within a normal range was important for PDAC patients to prolong survival.

### Integration with immune subtype revealed immune infiltrative features of ECM subtypes

PDAC is a kind of stroma-rich tumor. To investigate the relationship between tumor microenvironment (TME) and immune subtypes in PDAC, we performed integrative analysis to investigate the distinctive features of ECM subtypes [71]. For 226 patients with HE staining, the 48 patients (21.3%) were identified as desert-subtype, 85 patients (37.6%) were identified as reactive subtype, and 77 patients (34.1%) were identified as inter-subtype; 16 patients (7.0%) were defined as NEC (not elsewhere classified), respectively (Additional file 18: Fig. S18A).

To illustrate the diverse molecular features of different ECM subtypes, we performed comparative analysis among three ECM subtypes, utilizing both proteomic and phosphoproteomic data. Pathway enrichment analysis indicated that proteins upregulated in deserted subtype were enriched in focal adhesion, complement and coagulation cascades etc. In reactive subtype, proteins involved in DNA replication, DNA repair and chromatin remodeling were upregulated. The molecular characteristic of inter-subtype was intermediate between the two. At the phosphoproteome level, the three ECM subtypes exhibited similar characteristics as we observed at proteome level (Additional file 18: Fig. S18B).

To illustrate whether tumor purity was associated with ECM subtyping, we then compared the tumor purity among the ECM subtypes and found that deserted subtype had the lowest tumor purity (median = 40%) among the three types, reactive subtype had the highest tumor purity (median = 60%), and inter-subtype was between the two (median = 50%) (Additional file 18: Fig. S18C). Correspondingly, the expression of ECM marker proteins (COL1A1, COL8A1, and ENG) was significantly lower in reactive than in deserted subtype (Additional file 18: Fig. S18D). The lower expression patterns of ECM marker proteins (COL1A1 and MMP23B) in reactive subtype were further confirmed by IHC staining (Additional file 18: Fig. S18E). Different proportions of stroma in deserted, inter-, and reactive subtype are associated with different molecular characteristics [71]. In order to investigate the biological diversity between samples with high and low tumor purity, we divided the samples into high and low tumor purity group (high: 50%-80%, median: 70%; low: 10%-50%, median: 40%) and conducted both comparative proteomic and phosphoproteomic analysis between groups. As a result, we found that both proteins and phosphoproteins that enriched in the key pathways of tumorigenesis such as cell cycle, DNA replication, and RNA splicing were upregulated in patients with high tumor purity, whereas proteins that enriched in ECM–receptor interaction and focal adhesion were upregulated in patients with low tumor purity (Additional file 19: Fig. S19B). These results revealed the biological diversity of PDAC tumor with different tumor purity.

Importantly, in order to provide therapeutic advice to patients with different ECM subtypes, we further matched subtype specific proteins with known drug target protein. As a result, we observed MAP2K3 which could be targeted by Pimasertib, MAPK1 which could be targeted by Purvalanol, MMP9 which could be targeted by Marimastat were significantly elevated in reactive ECM subtypes (Additional file 18: Fig. S18F).

Additionally, to further illustrate the association between immune subtypes and ECM subtypes, we compared subtype assignment of our PDAC patients using each of the two classifiers. As a result, no significant overlap of samples between two subtypes was observed, yet the reactive subtype of ECM subtyping was orthogonally distributed across the three immune subtypes, suggesting that this class is not restricted to a specific immune feature (Additional file 18: Fig. S18G-H). Further survival analysis revealed that our immune subtyping could capture differences in survival in reactive subtype (Additional file 18: Fig. S18I). We then conducted comparative analysis and found the immune cell infiltration characteristics were consistent with immune subtypes (Additional file 18: Fig. S18J). Our immune subtype could further divide ECM subtype into different immune subtypes and identify the different immune infiltration of patients in the same ECM subtype.

In sum, the molecular characteristics were different among deserted, reactive, and inter-ECM subtype, which were associated with tumor purity. Our immune subtyping could help to illustrate the immune infiltration of diverse ECM subtypes and help to decipher the complexity and heterogeneity of patients belonging to the same ECM subtype.

### HOGA1 inactivation promotes PDAC growth through activating LARP7-CDK1 pathway

**Fig. 8:**
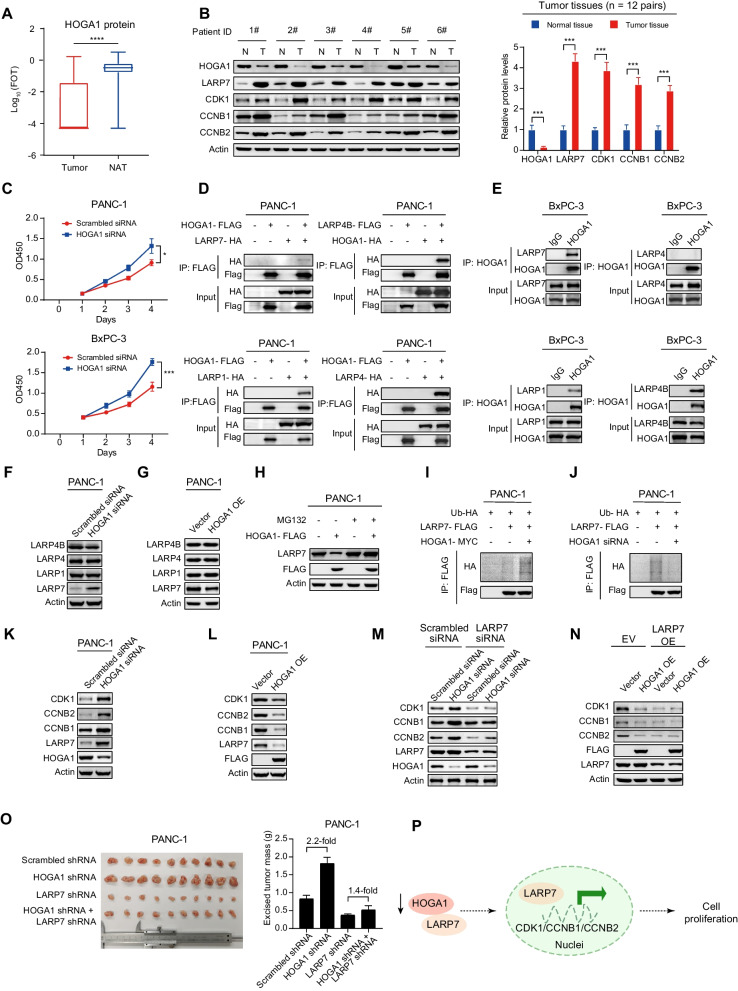
HOGA1 loss promotes pancreatic cancer growth via activating LARP7-CDK1 pathway. **A** Boxplot showing the differential expression of HOGA1 in tumors and NATs (t test). **B** Western blot analysis detecting the expression of HOGA1, LARP7, CDK1, CCNB1, and CCNB2, in tumor tissues and tumor-adjacent non-tumor tissues. N = tumor-adjacent non-tumor tissue, T = tumor tissue. The right panel shows quantified western blot results of the 12 pairs of samples. **C** Proliferation of PANC-1 and BxPC-3 cells associated with various treatments (n = 4 repeats per group). **D** Co-immunoprecipitation assay showing that exogenous HOGA1 and exogenous LARP1/4/4B/7 interact in the PANC-1 cells. **E** Co-immunoprecipitation assay showing that endogenous HOGA1 and endogenous LARP1/4/4B/7 interact in the BxPC-3 cells. **F**, **H** The expression levels of LARP1/4/4B/7 in PANC-1 cells with various treatments. **I**, **J** The ubiquitination levels of LARP7 in PANC-1 cells with various treatments. **K**–**N** The expression levels of CDK1, CCNB1, and CCNB2, in PANC-1 cells with various treatments. **O** Xenograft tumor image pictures (left) and tumor weight (right) of indicated PANC-1 cells subcutaneously injected into nude mice. **P** The systematic diagram summarizing the impact of the HOGA1 expression on cell proliferation. ****p < 1.0E−4, ***p < 1.0E−3, **p < 1.0E−2, *p < 0.05, ns > 0.05

We found that HOGA1 (4-hydroxy-2-oxoglutarate aldolase, mitochondrial) was downregulated by more than 80% in pancreatic cancer tumor tissues compared with non-tumor tissues in proteomic results (Student’s t test, *P* < 1.0E−4, FC = 0.14) (Fig. 8A), and western blotting of 12 pairs of pancreatic cancer tissues (Fig. 8B, Additional file 20: Fig. S20C, Additional file 30: Table S8A). We verified that HOGA1 expression in tumors was also significantly downregulated compared with NATs in CPTAC cohort [17] (Additional file 20: Fig. S20B). The expression of HOGA1 was association with better prognosis (Additional file 20: Fig. S20A). To investigate the role of HOGA1 in the development of pancreatic cancer, we constructed both HOGA1 overexpressed and HOGA1 knocked down PANC-1 and BxPC-3 cell lines and performed further analysis. As a result, overexpressed *HOGA1* slowed down cell proliferation in PANC-1 and BxPC-3 cells (Student’s t test, *P* = 6.1E−4, *P* = 1.1E−4, respectively) (Additional file 20: Fig. S20D, Additional file 30: Table S8C), whereas *HOGA1*-knocking down cells exhibited increased proliferation ability in comparison to control cells (Student’s t test, *P* = 0.01, *P* = 1.7E−4, respectively) (Fig. 8C, Additional file 30: Table S8B). HOGA1 is known as a mitochondrial protein and is one of the enzymes (4-hydroxy-2-oxoglutarate aldolase) involved in metabolism of hydroxyproline to glyoxylate [72]. To determine whether the metabolic enzymatic activity of HOGA1 is important for the expansion of tumor cells, we treated PANC-1 and BxPC-3 cells with hydroxyproline and glyoxylate, respectively, and found the neither of them affected the cell proliferation (Additional file 20: Fig. S20E, Additional file 30: Table S8D). Together with the observations that HOGA1 not only expressed in mitochondrial, but also located in cytosol (Additional file 20: Fig. S20F), these results suggested that the tumor suppressor role of HOGA1 was not due to its metabolic activity.

To investigate the potential non-metabolic role of HOGA1 in PDAC, we further utilized *HOGA1* overexpressed-, *HOGA1* knocked down-PANC-1 cell lines (PANC-1-*HOGA1*-OE, PANC-1-*HOGA1*-KD) and set PANC-1 cell lines without transfection (WT) as control (Additional file 21: Fig. S21A), and performed comparative proteomic analysis among PANC-1-*HOGA1-*KD, PANC-1-*HOGA1-*OE, and WT. As a result, 7878 proteins were detected across the different cell lines. The correlation among three replicates were 0.95. Comparing to the wild type PANC-1, a total of 220 proteins were significantly upregulated in PANC-1-*HOGA1*-KD. Meanwhile, 209 proteins were downregulated in PANC-1-*HOGA1*-OE. Further pathway enrichment indicated that, in concordant with the fast cell proliferation rates of PANC-1-*HOGA1-*KD, we found proteins that enriched in cell cycle process, DNA repair process showed significantly enhanced expression in PANC-1-*HOGA1*-KD, and decreased expression in PANC-1-*HOGA1*-OE, comparing to wild type PANC-1, suggested HOGA1 might impact tumor cell proliferation process (Additional file 21: Fig. S21B, Additional file 30: Table S8K). This assumption was further supported by the increased expression of CDKs (CDK1, CDK4, CDK5 and CDK6, etc.), MCMs (MCM2, MCM4, MCM6, MCM7 etc.) in PANC-1-*HOGA1*-KD, and decreased expression of them in PANC-1-*HOGA1*-OE, comparing to wild type PANC-1 (Additional file 21: Fig. S21B, Additional file 30: Table S8J). Intriguingly, the LARP7, which has been reported to regulated cell proliferation process in tumors [73], was found to be dominantly expressed in PANC-1-*HOGA1*-KD (Additional file 21: Fig. S21C).

We used tandem affinity purification to identify HOGA1-interacting proteins in PANC-1 cells. A total of 773 different proteins were detected in the cells (Additional file 30: Table S8E). GO analysis showed that most HOGA1-interacting proteins took part in the mRNA binding related pathways (Additional file 20: Fig. S20G, Additional file 30: Table S8F). Among them, we found La family RNA-binding proteins (LARPs) were enriched in HOGA1-interacting proteins, including LARP1, LARP4, LARP4B, and LARP7. Accordingly, the interactions between HOGA1 and those LARPs were confirmed via co-immunoprecipitation assays using either exogenous HOGA1 and LARPs in PANC-1 cells (Fig. 8D), or endogenous HOGA1 and LARPs in BxPC-3 cells (Fig. 8E). To explore whether HOGA1 affected the LARPs function, we examined the expression level of LARPs in HOGA1 knocking down and overexpressing PANC-1 cells. We found the protein levels of LARP7, but not other LARP members, increased significantly in HOGA1 knocking down cells (Fig. 8F). Conversely, HOGA1 overexpression resulted in decreased protein levels of LARP7 (Fig. 8G). LARP7 mRNA level did not change along with the HOGA1 levels (Additional file 20: Fig. S20H-I, Additional file 30: Table S8G), excluding the possibility that HOGA1 regulated LARP7 levels at the transcriptional level. Treatment with proteasome inhibitor MG132 elevated cellular LARP7 levels and prevented the degradation of LARP7 caused by HOGA1 overexpression (Fig. 8H). In addition, overexpression of HOGA1 increased the ubiquitination levels of LARP7 (Fig. 8I); in contrast, knock down of HOGA1 decreased the ubiquitination levels of LARP7 (Fig. 8J). These results indicated that HOGA1 regulated LARP7 expression by the ubiquitin proteasome pathway.

As a known oncogene, LARP7 promoted G2/M cell cycle transition and tumorigenesis via the CDK1 complex (Additional file 20: Fig. S20J, Additional file 30: Table S8H). We validated that knocking down HOGA1 resulted in increased levels of CDK1, CCNB1, and CCNB2 (Fig. 8K), and facilitated G2/M cell cycle transition in PANC-1 cells (Additional file 20: Fig. S20J). In contrast, overexpression of HOGA1 decreased the levels of CDK1, CCNB1, and CCNB2 (Fig. 8L). Moreover, depleting LARP7 abrogated the regulatory effects of HOGA1 on CDK1, CCNB1, and CCNB2 (Fig. 8M, N). In the pancreatic cancer tissues, we confirmed that protein levels of LARP7, CDK1, and CCNB1 increased notably in tumor tissues compared with non-tumor tissues (Fig. 8B, Additional file 20: Fig. S20C). In addition, we noted that loss of HOGA1 expression promoted the xenograft growth of PANC-1 cells, whereas knockdown of LARP7 abrogated the effect of HOGA1 and delayed the xenograft growth of tumor cells (Fig. 8O, Additional file 30: Table S8I). Taken together, these results indicated that HOGA1 loss in pancreatic cancer promoted cancer progress through activating LARP7-CDK1 pathway.

In summary, our study presented a comprehensive proteogenomic map of PDAC. The dominant pathway that was altered in the proteome subtypes of PDAC revealed the molecular mechanism underlying clinical phenotypes and outcomes. We identified a potential druggable protein, HOGA1, and demonstrated the value of this multi-omics approach. We believe that this study provides valuable information regarding biology of PDAC and enables discovery novel diagnostic and therapeutic strategies.

## Discussion

As an aggressive disease that typically presents at an advanced stage, PDAC is usually refractory to most treatment modalities. Despite the remarkable progress in precision oncology by genomic profiling of PDAC, the existing strategies targeting genetic abnormalities have limitations. A more comprehensive understanding of PDAC based on proteogenomic can fill the gap between genome abnormalities and oncogenic protein machinery. Here, we presented an integrative proteogenomic characterization of 217 paired tumor/NAT samples with 7 years of prognosis information that exhibited a range of clinic-pathologic spectrum of this disease, extending and refining analytical opportunities provided by prior studies.

Although multiple genomic studies have been performed on PDAC, the biomarker for accurately predicting the metastasis of PDAC is still lacking. Our data showed the amplification of *ADAM9* (*8p11.22*) had a critical role in PDAC metastasis. The role of ADAM9 in metastatic capacity of PDAC has not previously been reported. Our findings deciphered a novel mechanism that *ADAM9* amplification led to increase the epithelial cell migration-related proteins and break the cell–matrix interaction to promote PDAC metastasis. Furthermore, our data contributed to the understanding of the specific cooperation of ADAM9 and CDCP1 in activating WNT/β-catenin signaling pathway through SRC-CTNNB1_pS191. Indeed, recent studies demonstrated the WNT/β-catenin signaling pathway in promoting pancreatic cancer development [74]. Our results elucidated the potential role of ADAM9 and CDCP1 in activating WNT/β-catenin signaling pathway and contributed to the metastasis in PDAC. Hence, our systematic analysis not only proposed amplification of *ADAM9* which could be utilized as a biomarker for PDAC metastasis, but also offered support for the utilization of SRC inhibitor (Bosutinib) in the treatment of pancreatic cancer.

Given the clear importance of the TME in tumorigenesis, understanding the multi-faceted roles of the complex TME components has garnered much attention. Recently, integrated proteogenomic provided a valuable resource for investigating TME [75]. Immune classification allowed the description of 5 PDAC subtypes (Im-S-I-V). Our results included protein signatures and cellular pathways for each subtype, as well as distinctive immune cell infiltration. For example, Im-S-IV&V with favorable prognosis showed elevated TCA cycle and cDC cell infiltration. These characteristics have been suggested to be caused by the *cis*-effect of *FH* (Fumarate hydratase). Since a hallmark of PDAC is dependency on cellular metabolic pathways for tumor growth [76], it is important to decipher the mechanism of metabolic alteration in PDAC tumors. The integrative proteogenomic analysis identified the amplification of *FH* as a previously undocumented metabolic regulator of PDAC. Further analysis indicated the *FH* amplification functioned by increasing the rate-limiting enzymes in TCA cycle, and upregulating the glucose transporter 4 (GLUT4)-mediated glucose transport and consequentially decreasing patients’ blood glucose. Although clinical researches have indicated patients with low blood glucose concentrations harbored favorable prognosis, the mechanism has not been illustrated [77]. Our findings illustrated the importance of blood glucose control in treating PDAC.

Here, we showed that HOGA1, which was previously known as a mitochondrial protein and involved in metabolism of hydroxyproline to glyoxylate, contributed to tumor progression through its signaling role. Targeting LARP7 was able to intercept the tumor-promoting effect of HOGA1 loss and avoid affecting glyoxylate metabolism. HOGA1 functions like a tumor suppressor and HOGA1 decreased notably in pancreatic cancer, indicating the loss of HOGA1 might affect PDAC progression. Therefore, the decreased expression of HOGA1 might be a diagnostic indicator of PDAC, and inhibiting LARP7, which collaborated with HOGA1, would provide another feasible strategy for suppressing pancreatic cancer development in clinic.

The aims of this study were to characterize PDAC tumors and NATs using a multi-omics synthesis and to provide a proteogenomic resource to decipher the impact of genomic alterations in gene expression, protein abundance, and phosphorylation modification. There are some limitations to a study of this type. (1) The information of PDAC remote metastasis came from 80 months of follow-up, and samples used in our study were primary tumor without remote metastatic samples. The conclusion that ADAM9 promotes metastasis requires further validation in metastatic samples. (2) The proteomic measurements in this study were performed from PDAC tumor and NAT tissues, while the effects of intratumoral heterogeneity were not fully accessed. Integrating single-cell and spatial omics would be useful to further explore the mechanism of pancreatic cancer progression in the future.

We hope that the specific observations and hypotheses described in this manuscript, and the data that underlie them, will serve as a rich resource for those studying PDAC and for the larger research community, including for the development of targeted chemotherapy or immunotherapy.

In summary, the proteogenomic analysis presented here demonstrated that proteomic data provided additional insights beyond those available with genomic data alone, improving our understanding of PDAC and stratification of PDAC patients, while also identifying potentially useful diagnostic and therapeutic targets.

## Conclusions

In summary, integrated multi-omics analysis has improved our understanding of PDAC and stratification of PDAC patients, while also identified potentially useful diagnostic and therapeutic targets. We found that *TP53* mutations led to poor prognosis in younger PDAC patients by upregulating CDK4-mediated cell proliferation process. The *ADAM9* (*8p11.22*) gain could drive PDAC metastasis. Proteome-based stratification of PDAC revealed three subtypes (S-I, S-II, and S-III) related to different clinical and molecular features. Analysis of the immune subtypes of PDAC revealed a metabolic tumor subgroup that harbored *FH* amplicons led to better prognosis through increased glucose transport and enhanced TCA cycle process. Finally, we found that loss of HOGA1 promoted the tumor growth via activating LARP7-CDK1 pathway. These results suggested that HOGA1 could be a new therapeutic target in PDAC.

## Methods

### Sample selection

The tumor tissue and normal adjacent tissue samples used in this study were obtained from the Zhongshan Hospital, Fudan University. The present study was carried out in compliance with the ethical standards of Helsinki Declaration II and approved by the Institution Review Board of Fudan University Zhongshan Hospital (B2019-200R). A total of 229 patients were randomly selected from January 2012 to December 2016 with pancreatic ductal adenocarcinomas, who were undergoing surgical resection. All cases had PDAC histology but collected regardless of histologic grade or surgical stage. Patients were excluded if they had advanced disease, active second malignancy, or received any prior treatment, such as radiotherapy or chemotherapy. Clinical information of 229 patients, including gender, age, degree of differentiation, TNM stage (AJCC cancer staging system 8th edition), cancer metastasis status, survival status, drinking status, smoke status, vessel invasion, postoperative radiotherapy, postoperative chemotherapy, hypertension, diabetes, preoperative jaundice, CA19-9, metastatic site, is listed in Additional file 23: Table S1. In particular, only patients with diabetes > 1 year were included, patients with recent onset diabetes were not considered. Patients were classified as TNM stages I, II, and III, respectively. Our cohort did not include clinically stage IV (metastatic) patients. The information of remote metastasis came from 80 months of follow-up. And samples used in our study were primary tumor without remote metastatic samples. Each sample was assigned a new research ID, and the patient’s name or medical record number used during hospitalization was de-identified.

### Sample preparation

Formalin-fixed, paraffin-embedded (FFPE) specimens were prepared and provided by Zhongshan hospital. One 3-μm-thick slide from FFPE blocks was sectioned for hematoxylin and eosin (H&E) staining. For genomic, proteomic and phosphoproteomic sample preparation, 8-μm-thick slides were sectioned, deparaffinized with xylene and washed with gradient ethanol. Samples were just sectioned by 8-μm-thick slides without xylene deparaffinization nor gradient ethanol wash for RNA sample preparation. Selected specimens according to H&E staining were scraped, and materials were aliquoted and kept in storage at − 80 °C until further processing.

### Tumor purity

Histology of the tumor and normal adjacent tissues was checked by two expert pathologists to confirm the sample quality according to the following standards: (1) Pathologically defined PDAC tumors; (2) no tumor cells in the normal adjacent tissues. Selected specimens according to H&E staining were scraped. Information regarding tumor histological subtype, grade, and tumor purity was provided. The samples used for multi-omics analysis were characterized with histologic tumor purity ranged from 10 to 80% (median = 50%). The tumor purity of all samples is presented in Additional file 23: Table S1B, and examples of H&E-stained tumor are presented in Additional file 22: Fig. S22.

Both ABSOLUTE and ESTIMATE algorithms were utilized to evaluate the overall computational purity score for each sample. Computational tumor purity was inferred by R package ESTIMATE v1.1.0 [78] and ABSOLUTE [79] using proteome/mRNA data and WES data, respectively.

### Whole-exome sequencing

DNA extraction and DNA quantification. DNA from the 149 paired tumor/NAT specimens was extracted according to the manufacturer’s instructions of QIAamp DNA Mini Kit (QIAGEN, Hilden, Germany). The quality of the isolated and contamination was verified by the following methods:

1. DNA degradation and contamination were monitored on 1% agarose gels.
2. DNA concentration was measured by Qubit® DNA Assay Kit in Qubit® 2.0 Fluorometer (Invitrogen, CA, USA).

Library preparation and DNA sequencing. A total amount of 0.2 µg genomic DNA per sample was used as input material for the DNA preparation. For whole-exome sequencing, libraries were generated by using Agilent SureSelect Human All Exon kit (Agilent Technologies, CA, USA) following manufacturer’s recommendations and index codes were added to each sample. Briefly, fragmentation was carried out by hydrodynamic shearing system (Covaris, Massachusetts, USA) to generate 180–280 bp fragments. Remaining overhangs were converted into blunt ends via exonuclease/polymerase activities. After adenylation of 3′ ends of DNA fragments, adapter oligonucleotides were ligated. DNA fragments with ligated adapter molecules on both ends were selectively enriched in a PCR reaction. After PCR reaction, libraries hybridize with liquid phase with biotin labeled probe and then use magnetic beads with streptomycin to capture the exons of genes. Captured libraries were enriched in a PCR reaction to add index tags to prepare for sequencing. Products were purified using AMPure XP system (Beckman Coulter, Beverly, USA) and quantified using the Agilent high sensitivity DNA assay (Agilent) on an Agilent Bioanalyzer 2100 system (Agilent Technologies, CA, USA).

Clustering and Sequencing. The clustering of the index-coded samples was performed on a cBot Cluster Generation System using a HiSeq PE Cluster Kit (Illumina) according to the manufacturer’s instructions. After cluster generation, the DNA libraries were sequenced on Illumina NovaSeq 6000 platform and 150 bp paired-end reads were generated.

Whole-exome Sequencing Quality Control. The original fluorescence image files obtained from NovaSeq 6000 platform are transformed to short reads (Raw data) by base calling, and these short reads are recorded in FASTQ format, which contains sequence information and corresponding sequencing quality information. Sequence artifacts, including reads containing adapter contamination, low-quality nucleotides, and unrecognizable nucleotide [80], undoubtedly set the barrier for the subsequent reliable bioinformatics analysis. Hence, quality control is an essential step and applied to guarantee the meaningful downstream analysis.

The steps of data processing were as follows:

1. Discard the paired reads if either one read contains adapter contamination (> 10 nucleotides aligned to the adapter, allowing ≤ 10% mismatches).
2. Discard the paired reads if more than 10% of bases are uncertain in either one read.
3. Discard the paired reads if the proportion of low quality (Phred quality < 5) bases is over 50% in either one read.

All the downstream bioinformatics analyses were based on the high-quality clean data, which were retained after these steps. At the same time, QC statistics including total reads number, raw data, raw depth, sequencing error rate, percentage of reads with Q30 (the percent of bases with Phred-scaled quality scores greater than 30) and GC content distribution were calculated and summarized. WES was conducted with mean coverage depths of 179X (range 92-444X) for tumor samples and 149X (range 68-386X) for adjacent non-tumor samples, which is consistent with the recommendations for WES [81].

### Genomic variant calling

WES sequencing reads after the exclusion of low-quality reads were mapped to the UCSC hg19 reference sequence with Burrows-Wheeler Aligner (BWA) software to obtain the original mapping results stored in BAM format [82, 83]. Then, SAMtools was used to sort the BAM files and perform duplicate marking, local realignment, and base quality recalibration to generate the final BAM file for computing the sequence coverage and depth [84]. ANNOVAR was performed to annotate the Variant Call Format file obtained in the previous step [85].

Filter conditions were set to identify the candidate genetic alterations as follows:

1. Remove mutations with coverage less than 10 × ;
2. Remove variant sites in dbSNP and with mutant allele frequency (MAF) > 0.001 in the 1000 Genomes databases (1000 Genomes Project Consortium) and the Novo-Zhonghua (in-house unrelated healthy individual database), but include sites with MAF ≥ 0.001 and < 0.1 with COSMIC evidence (http://cancer.sanger.ac.uk/cosmic) [86–88];
3. Variations in the exosmic or splicing region (10 bp upstream and downstream of splicing sites);
4. Remove synonymous mutations;
5. Retain the non-synonymous SNVs if the functional predictions by PolyPhen-2, SIFT, MutationTaster, and CADD all show the SNV is not benign [89–92];
6. Retain genes identified by Cancer Gene Census (CGC, http://www.sanger.ac.uk/science/data/ cancer-gene-census).

### Somatic variant calling

For the 149 PDAC tumor samples, we referred to previous published papers [93, 94] and developed a variant selection pipeline to detect somatic variant calling.

1. Known constitutional polymorphisms using known human variation databases, 1000 Genomes databases (1000 Genomes Project Consortium) and the Novo-Zhonghua (in-house unrelated healthy individual database) [87];
2. Known somatic variation in PDAC and other common malignancies as reported in COSMIC V90 [95];
3. The presence of the same sequence change in exome or whole genome sequencing data derived from 186 constitutional DNA samples analyzed in CGP (CGP normal panel); specifically, where the same base change was observed in at least two constitutional sample at allele fractions greater than 10%, the variant has not previously been confirmed as somatic in COSMIC or in two or more samples at < 10%.
4. Sequence contexts 5′ and 3′ to the reported sequence change highlighting regions of homopolymer sequence that are prone to PCR slippage and artifacts altering the last base of the homopolymer or inserting the same base as the homopolymer at + 1, + 2 of the tracks and often present in unidirectional reads and < 10% variant allele burden;
5. Variant specific metrics to include protein annotation, sequence depth, and % of reads reporting the variant allele.

### Effects of copy number alternations

GISTIC2 was applied to identify significantly amplification/deletion regions and genes based on UCSC known genes. Genomic alterations impacted mRNA and protein abundance at the same locus were defined as *cis*-effects, while the impact of other loci was defined as *trans*-effects.

### Copy-number alterations analysis

Exome-based somatic copy number alteration (SCNA) was called by following somatic CNA calling pipeline in GATK’s (GATK 4) Best Practice. The results of this pipeline and segment files of every 1000 were input in GISTIC2, to identify significantly amplified or deleted regions across all samples, which could be accumulated driving regions. To exclude false positives as much as possible, relatively stringent cutoff thresholds were used with parameters: -ta 0.5 -tb 0.5 -brlen 0.5 -conf 0.95. Other parameters were the same as default values.

### Detection and calling of somatic mutations

BWA and SAMtools were used for genome alignment, and muTect Software [96] was used for identifying the Somatic SNV sites, whereas Strelka [97] was used to detect the Somatic InDels. Control-FREEC was used to detect SCNAs. SAMtools mpileup and bcftools were used for the variant calling and to identify the SNPs and InDels. Statistical analysis included two-tailed Student’s t test and Fisher’s exact test.

### Mutation signature analysis

Based on the single nucleotide substitution and its adjacent bases pattern of samples, frequencies of 96 possible mutation types for each sample could be estimated. Nonnegative matrix factorization (NMF) algorithm was used to estimate the minimal components that could explain maximum variance among samples. Then each component was compared to mutation patterns of 30 validated cancer signatures reported from the COSMIC database (https://cancer.sanger.ac.uk/signatures) individually to identify cancer-related mutational signatures and carcinogen signatures. Cosine similarity analysis [98, 99] was used to measure the similarity between component and signatures, which ranged from 0 to 1, indicating maximal dissimilarity to maximal similarity. After decomposing matrix of samples’ 96 substitution classes into 5 signatures, contribution of signatures in each sample could be estimated.

### RNA extraction

RNA was extracted from tissues by using TIANGEN® RNAprep Pure FFPE Kit (#DP439) according to the reagent protocols. For library preparation of RNA sequencing, a total amount of 500 ng RNA per sample was used as the input material for the RNA sample preparations. Sequencing libraries were generated using Ribo-off® rRNA Depletion Kit (H/M/R) (Vazyme #N406) and VAHTS® Universal V6 RNA-seq Library Prep Kit for Illumina (#N401-NR604) following the manufacturer’s recommendations, and index codes were added to attribute sequences to each sample. The libraries were sequenced on an Illumina platform and 150 bp paired-end reads were generated.

### RNA-Seq data analysis

RNA-seq raw data quality was assessed with the FastQC (v0.11.9), and the adaptor was trimmed with Trim_Galore (version 0.6.6) before any data filtering criteria was applied. Reads were mapped onto the human reference genome (GRCh38.p13 assembly) by using STAR software (v2.7.7a). The mapped reads were assembled into transcripts or genes by using StringTie software (v2.1.4) and the genome annotation file (hg38_ucsc.annotated.gtf). For quantification purpose, the relative abundance of the transcript/gene was measured by a normalized metrics, FPKM (Fragments Per Kilobase of transcript per Million mapped reads). Transcripts with an FPKM score above one were retained, resulting in a total of 23,655 gene IDs. All known exons in the annotated file were 100% covered.

### Protein extraction and tryptic digestion

Lysis buffer [0.1 M Tris–HCl (pH 8.0), 0.1 M DTT (Sigma, 43,815), 1 mM PMSF (Amresco, M145)] was added to the extracted tissues and subsequently sonicated for 1 min (3 s on and 3 s off, amplitude 25%) on ice. The supernatants were collected, and the extracted tissues were then lysed with 4% sodium dodecyl sulfate (SDS) and kept for 2 h at 99 °C with shaking at 1500 rpm. The solution was collected by centrifugation at 12,000 × *g* for 5 min. A fourfold volume of acetone was added to the supernatant and kept in -20 °C for a minimum of 4 h. Subsequently, the acetone-precipitated proteins were washed three times with cooled acetone and then pumped out using the Concentrator plus (Eppendorf, Germany). Filter-aided sample preparation (FASP) procedure was used for protein digestion [100]. The proteins were resuspended in 200 μL 8 M urea (pH 8.0) and loaded twice in 30 kD Microcon filter tubes (Sartorius) and centrifuged at 12,000*g* for 20 min. The precipitate in the filter was washed twice by adding 200 μL 50 mM NH_4_HCO_3_. The precipitate was resuspended in 50 μL 50 mM NH_4_HCO_3_. Protein samples underwent trypsin digestion (enzyme-to-substrate ratio of 1:50 at 37 °C for 18–20 h) in the filter and then were collected by centrifugation at 12,000*g* for 15 min. Additional washing, twice with 200 μL of MS water, was essential to obtain greater yields. Finally, the centrifugate was dried by using the Concentrator plus (Eppendorf, Germany).

### First dimensional reversed-phase separation

The dried peptides were re-dissolved with 100μL 10 mM NH_4_HCO_3_ and loaded into a homemade Durashell Reverse Phase column [2 mg packing (3 μm, 150 Å, Agela) and two C18 layers (12 μm, Empore) in a 200 μL tip] and then eluted sequentially with nine gradient elution buffer that contains 6%, 9%, 12%, 15%, 18%, 21%, 25%, 30%, 35% ACN (diluted with 10 mM NH_4_HCO_3_, adjusted pH to 10.0 using NH_3_^.^H_2_O). The nine fractions then were combined into three groups (6% + 15% + 25%, 9% + 18% + 30%, 12% + 21% + 35%) and dried under Concentrator plus (Eppendorf, Germany) for sub-sequential MS analysis.

### Phosphopeptide enrichment

For the phosphoproteomic analysis, peptides were extracted from the FFPE slides after trypsin digestion using the methods described above. The tryptic peptides were then enriched with High-Select™ Fe-NTA Phosphopeptide Enrichment Kit (Thermo Scientific cat. A32992), following the manufacturer’s recommendation. Briefly, peptides were suspended with binding/wash buffer (contained in the enrichment kit), mixed with the equilibrated resins, and incubated at 21–25 °C for 30 min. After incubation, the resins were washed thrice with binding/wash buffer and twice with water. The enriched peptides were eluted with elution buffer (contained in the enrichment kit) and dried in a Concentrator plus (Eppendorf).

### LC–MS/MS analysis

For the proteome profiling samples, the peptide samples were analyzed using Q Exactive HFX LC–MS/MS analyses on an Easy-nLC 1200 liquid chromatography system (Thermo Fisher Scientific) coupled to a Q Exactive HFX via a nano-electrospray ion source (Thermo Fisher Scientific). Dried peptide samples re-dissolved in buffer A (0.1% FA in water) were loaded to a 2-cm self-packed trap column using buffer A and separated on a 15 cm long, homemade C18 nano-capillary analytical column (75 mm inner diameter) packed with C18 resin (particle size: 3 μm, pore size: 100 Å; Dikma Technologies Inc., Lake Forest, CA, USA). The peptides were separated onto an analytical column with a 75 min gradient (buffer A: 0.1% formic acid in water; buffer B: 0.1% formic acid in 80% ACN) at a constant flow rate of 600 nL/min (0–75 min, 0 min, 4% B; 0–10 min, 4–15% B; 10–60 min, 15– 30% B; 60–69 min, 30–50% B; 69–70 min, 50–100% B; 70–75 min, 100% B). The eluted peptides were ionized under 2 kV and subjected to MS. MS analysis was performed in a data-dependent manner with full scans (m/z 300–1400) acquired using an Orbitrap mass analyzer at a mass resolution of 120,000 at m/z 400. The top 20 precursor ions were selected for fragmentation in an HCD cell at a normalized collision energy of 32%, and then fragment ions were transferred into the Orbitrap analyzer operating at a resolution of 7500 at m/z 400. The automatic gain control (AGC) for full MS was set to 3e6, and that for MS/MS was set to 5e4, with maximum ion injection times of 20 and 60 ms, respectively. The dynamic exclusion of previously acquired precursor ions was enabled at 18 s.

For the phosphoproteomic analysis, the phosphopeptides were analyzed on FAIMS interfaced Orbitrap Fusion Lumos Tribrid Mass Spectrometer (Thermo Fisher Scientific, Rockford, IL, USA) equipped with an Easy nLC-1000 (Thermo Fisher Scientific, Rockford, IL, USA) and a Nanoflex source (Thermo Fisher Scientific, Rockford, IL, USA). Dried peptide samples re-dissolved in buffer A (0.1% FA in water) were loaded to a 2 cm self-packed trap column using buffer A and separated on a 150 μm inner diameter column with a length of 30 cm over a 150 min gradient (buffer A: 0.1% FA in water; buffer B: 0.1% FA in 80% ACN) at a constant flow rate of 600 nL/min (0–150 min, 0 min, 4% B; 0–10 min, 4–15% B; 10–125 min, 15–30% B; 125–140 min, 30–50% B; 140–141 min, 50–100% B; 141–150 min, 100% B). The eluted phosphopeptides were ionized and detected. Compensation Voltages (CV) among -30 V, -60 V, and -120 V were interrogated to find precursor rich CVs. Mass spectra were acquired over the scan range of m/z 350–1500 at a resolution of 120,000 (AUG target value of 5E5 and max injection time 50 ms). For the MS2 scan, the higher-energy collision dissociation fragmentation was performed at a normalized collision energy of 30%. The MS2 AGC target was set to 1e4 with a maximum injection time of 10 ms, peptide mode was selected for monoisotopic precursor scan, and charge state screening was enabled to reject unassigned 1 + , 7 + , 8 + , and > 8 + ions with a dynamic exclusion time of 45 s to discriminate against previously analyzed ions between ± 10 ppm.

### Peptide identification and protein quantification

MS raw files generated by LC–MS/MS were processed with “Firmiana” (a one-stop proteomic cloud platform (https://phenomics.fudan.edu.cn/firmiana/) [101] software utilizing Mascot search engine against the human NCBI reference proteome database (updated on 04–07-2013). Protease was Trypsin/P. The maximum number of missed cleavages was set to two. A mass tolerance of 10 ppm for precursor and 50 mmu for production was allowed. The fixed modification was carbamidomethyl (C), and the variable modifications were acetylation (Protein N-term) and oxidation (M). For the phosphoproteomic data, variable modifications were oxidation (M), acetylation (Protein N-term) and phospho (S/T/Y). The cutoff of false discovery rate (FDR) by using a target-decoy strategy was 1% for peptide. Each peptide was assigned either as a unique peptide to a particular protein group or set as a razor peptide to a single protein group with the most peptide evidence. The protein groups assembled by “Firmiana” were filtered to 1% protein-level FDR also using target-decoy strategy. In generating site-level reports (phosphopeptide-enriched data), sites were computed localization probability using ptmRS [102] algorithm. Sites probability equal or greater than 0.75 were considered as confidently localized.

### Quality control of the mass spectrometry data

For the quality control of the performance of mass spectrometry, the HEK293T cell (National Infrastructure Cell Line Resource) lysate was measured every three days as the quality control standard. The quality control standard was digested and analyzed using the same method and conditions. A pairwise Spearman’s correlation coefficient was calculated for all quality control runs in the statistical analysis environment R (version 4.0.2). The average correlation coefficient among the standards was 0.95, demonstrating the consistent stability of the mass spectrometry platform.

### Principal component analysis

To elucidate the multi-omics difference between PDAC tumors and NATs and reveal molecular alterations upon PDAC tumorigenesis, we performed principal component analysis (PCA) on all three omics levels (transcriptome, proteome, and phosphoproteome) between tumors and NATs. The PCA function under the scikit-learn R package was implemented for unsupervised clustering analysis with the parameter “n_components = 2” on the expression matrix of global proteomic data containing 19,992 mRNAs, 7055 proteins, or 7399 phosphoproteins. The 95% confidence coverage was represented by a colored ellipse for each group, which was calculated based on the mean and covariance of points in each specific group (tumor and NAT).

To elaborate the proteomics data difference based on sample collection time and experimental batches, PCA of proteomic data was conducted based on years of sample collection and experimental batches. The PCA function under the scikit-learn R package was implemented for unsupervised clustering analysis with the parameter “n_components = 2” on the expression matrix of global proteomic data containing 7055 proteins.

### Global Heatmap

We applied two-way hierarchical clustering to the global proteomic data on samples and mRNA/proteins/phosphoproteins to identify the global differential protein expression. Each protein expression value in the global proteomic expression matrix was transformed to a z-score across all samples. For the sample-wise and protein-wise clustering, distance was set as “Euclidean” distance, and the weight method was “complete.” The z-score-transformed matrix was clustered using R package: pheatmap (version 1.0.12).

### Differential protein analysis

Student’s t test was used to examine whether proteins were differentially expressed between the tumors and NATs. Upregulated or downregulated proteins in tumors were defined as proteins differentially expressed in tumors compared with NATs (T/NAT > 2 or < 1/2, two-tailed Student’s t test, Benjamini–Hochberg adjusted p < 0.05). Wilcoxon rank-sum test was used to examine whether proteins were differentially expressed between patients with different mutation statuses (Benjamini–Hochberg adjusted p < 0.05). Wilcoxon rank-sum test was also used to examine whether proteins were differentially expressed between patients with diabetes history or not (Benjamini–Hochberg adjusted p < 0.05). One-way ANOVA test was used to examine whether proteins were differentially expressed among three proteomic subtypes and immune subtypes (Benjamini–Hochberg adjusted p < 0.05).

### Pathway enrichment analysis

Differential expressed proteins identified in tumor or NAT were subjected to Gene Ontology and KEGG pathway enrichment analysis in DAVID (https://david.ncifcrf.gov/) with the *P* value < 0.05.The significance of the pathway enrichment analysis was determined by Fisher’s exact test on the basis of KEGG pathways and categorical annotations, including the GO “biological process” term.

### Cell cycle analysis

Multi-gene proliferation scores (MGPSs) were calculated as the mean expression level of all cell cycle-regulated genes in each sample as described previously [103, 104]. Briefly, MGPS was calculated from the mean normalized proteomic data in each sample in our study.

### Kinase activity score calculation

To investigate the kinase activity of PDAC patients, we used gene set variation analysis (GSVA) to estimate the kinase activity, which define an activity score calculated from the degree of expression of a substrate’s set in each sample within the given dataset. Substrates of every kinase were collected from the PhosphoSitePlus database (version 6.6.0.4).

### Phosphopeptide analysis–kinase and substrate regulation

KSEA algorithm was used to estimate the kinase activities based on the abundance of phosphosites. Kinase–substrate enrichment analysis (KSEA) estimates changes in a kinase’s activity by measuring and averaging the amounts of its identified substrates instead of a single substrate, which enhances the signal-to-noise ratio from inherently noisy phosphoproteomic data [105, 106]. If the same phosphorylation motif was shared by multiple kinases, it was used for estimating the activities of all known kinases. The use of all curated substrate sequences of a particular kinase minimizes the overlapping effects from other kinases and thus improves the precise measurement of kinase activities. The information of kinase-substrate relationships was obtained from publicly available databases including PhosphoSite [107], Phospho.ELM [108], and PhosphoPOINT [109]. The information of substrate motifs was obtained either from the studies [110] or from an analysis of KSEA dataset with Motif-X [105].

### Protein–protein interaction network construction

Interaction network among the proteins and phosphorylated proteins was generated with STRING v 11.0 (https://string-db.org/) using medium confidence (0.4), and experiments and database as the active interaction sources. The network was visualized using Cytoscape version 3.8.0 [111].

### Immunohistochemistry (IHC)

Formalin-fixed, paraffin-embedded tissue sections of 10 µm thickness were stained in batches for detecting YAP1_pS109, RB1_pS807, ADAM9, CDCP1, ACOX1, S100A4, MCM6, COL1A1, and MMP23B in a central laboratory at the Zhongshan Hospital according to standard automated protocols. Deparaffinization and rehydration were performed, followed by antigen retrieval and antibody staining. IHC was performed using the Leica BOND-MAX auto staining system (Roche). Antibody was introduced, followed by detection with a Bond Polymer Refine Detection DS9800 (Bond). Slides were imaged using an OLYMPUS BX43 microscope (OLYMPUS) and processed using a ImageScope (Leica).

- YAP1_pS109: Phospho-YAP (Ser109) (E5I9G) Rabbit mAb (#53,749) (Cell Signaling)
- RB1_pS807: Phospho-Rb (Ser807/811) (D20B12) Rabbit mAb (#8516) (Cell Signaling)
- E2F1: rabbit monoclonal anti-E2F1 (ab288369) antibody (Abcam)
- E2F1_pS364: Phospho-E2F1(aa 350–450) Rabbit polyclonal antibody (ab5391) (Abcam)
- ADAM9: rabbit polyclonal anti-ADAM9 (ab186833) antibody (Abcam)
- CDCP1: rabbit monoclonal anti-CDCP1 (ab252947) antibody (Abcam)
- ACOX1: rabbit polyclonal anti-ACOX1 (10,957–1-AP) antibody (Proteintech)
- S100A4: rabbit polyclonal anti-S100A4 (16,105–1-AP) antibody (Proteintech)
- MCM6: rabbit polyclonal anti-MCM6 (13,347–2-AP) antibody (Proteintech)
- COL1A1: mouse monoclonal anti-collagen type I (67,288–1-Ig) antibody (Proteintech)
- MMP23B: rabbit polyclonal anti-MMP23B (13,020–1-A) antibody (Proteintech)

### Weighted gene co-expression network analysis (WGCNA)

WGCNA was performed on the entire 161 samples using R package [112] to identify differentially co-expressed gene modules. A soft threshold at power 5 was selected based on scale free topology model fit (R^2^ = 0.8). Gene modules functional annotation was performed using ConsensusPathDB (http://cpdb.aamolgen.mpg.de/) [113]. The eigengenes of each module were used to measure the association between a module and clinical information. Pearson correlations of each module and clinical information were further analyzed.

### ECM subtyping

ECM subtypes of tumor tissues were checked by two expert pathologists following standards: (1) “deserted” regions with thin, spindle-shaped fibroblasts, loose matured fibers, and often keloid or myxoid features, (2) “reactive” regions containing plump fibroblasts with enlarged nuclei, few acellular components, often rich in inflammatory infiltrate, (3) regions with intermediate levels of these features [71]. The information of ECM subtypes is in Additional file 23: Table S1B.

### Consensus clustering analysis

The protein expression matrix of the 217 paired tumor samples was used to identify the proteomic subtypes using the consensus cluster method. Consensus clustering was performed using the ConsensusClusterPlus R package [57] with the top 50% standard deviations proteins (n = 3527). The following detail settings were used for clustering: number of repetitions = 10,000 bootstraps; pItem = 0.8 (resampling 80% of any sample); pFeature = 1 (resampling 100% of any protein); and clusterAlg = “hc”; and distance = “spearman.” The number of clustering was determined by three factors, the average pairwise consensus matrix within consensus clusters, the delta plot of the relative change in the area under the cumulative distribution function (CDF) curve, and the average silhouette distance for consensus clusters. We selected a 3-cluster as the best solution for the consensus matrix with k = 3 or k = 4 deemed to be a cleanest separation among clusters, but the average silhouette distance for k = 3 was larger than k = 4 or k = 5 and did not have significant negative values. Based on the evidence above, the PDAC proteomic data were clustered into 3 groups (Additional file 11: Fig. S11A-B).

For the transcriptomic and phosphoproteomic data, the top 50% standard deviations genes within the tumor tissues were selected for subtyping. Here as well, we performed consensus clustering and set the same parameters as that for the proteome subgrouping. k = 3 provided the clearest separation among the clusters, and we selected three clusters as the best solution for the consensus matrix (Additional file 11: Fig. S11A).

### Survival analysis

Kaplan–Meier survival curves (log-rank test) were used for OS or DFS of the proteomic subtypes and patients. *P* value less than 0.05 was considered as significantly different. Prior to the log-rank test of a given protein, phosphoprotein, or phosphosite, survminer 0.2.4 R package with maxstat (maximally selected rank statistics) (http://r-addict.com/2016/11/21/Optimal-Cutpoint-maxstat.html) was used to determine the optimal cutpoint for the selected samples according to the previous study [114]. DFS or OS curves were then calculated (Kaplan–Meier analysis, log-rank test) based on the optimal cutpoint. This method was used in the previous studies, such as Xu et al. *Cell* [115].

### Immune subtype identification

The abundances of 64 different cell types for PDAC samples in mRNA level were computed via xCell (https://xcell.ucsf.edu/). Therefore, for this analysis, 54 PDAC tumor samples with mRNA data were utilized. Based on these 64 signatures, consensus clustering was performed in order to identify groups of samples with similar immune/stromal characteristics. Consensus clustering was performed using the R packages ConsensusClusterPlus [57]. Consensus Cluster Plus parameters were reps = 10,000, pItem = 0.8, pFeature = 1, clusterAlg = “hc,” distance = “spearman.” As summarized in Fig. 7, the clustering analysis of the tumors (vertical column) by xCell score (horizontal rows) divided 54 PDAC tumors into five immune clusters. A consensus matrix with k = 5 appeared to have the clearest cut between clusters and showed significant association with the patients’ survival.

### Screening potential drug targets

The potential clinical utility of those proteins is annotated in Additional file 6: Fig. S6E, Additional file 18: S18F and Fig. 6R, supported by the annotation in the DrugBank database (https://www.dgidb.org/sources/DrugBank).

### Functional experiments

#### Cell culture

PANC-1, SW1990, and BxPC-3 cells were purchased from ATCC. Dulbecco’s modified Eagle’s medium (DMEM) was used to culture the PANC-1 and SW1990, which also contained 10% fetal bovine serum (FBS), 100 U/ml penicillin, and 100 mg/ml streptomycin (HyClone; GE Healthcare Life Science). Roswell Park Memorial Institute (RPMI 1640 Medium) was used to culture the BxPC-3, which also contained 10% fetal bovine serum (FBS), 100 U/ml penicillin, and 100 mg/ml streptomycin (HyClone; GE Healthcare Life Science). All the cells were maintained at 37 °C in a humidified 5% CO_2_ atmosphere.

#### Plasmids

The sequence of human HOGA1, LARP1, LARP4, and LARP7 open reading frame was obtained using Polymerase chain reaction (PCR) from CDNA. The PCR fragment was inserted into Pcdna3.1-FLAG, Pcdna3.1-HA, Pcdna3.1-myc, or pLVXyu-BirA by the recombinant method and was confirmed by sequencing identification.

- BioID-HOGA1-F:5′-GTCGTCGTCGTCGTCGAATTCATGCTGGGTCCCCAAGTCTG-3′
- BioID-HOGA1-R:5′-AGAGGGGCGGGATCCGAGCCAGCCGTTGCTGGT -3′
- HOGA1-F:5′-AACGGGCCCTCTAGACTCGAGATGCTGGGTCCCCAAGTCT-3′
- HOGA1-R:5′-TAGTCCAGTGTGGTGGAATTCGAGCCAGCCGTTGCTGGT-3′
- LARP7-F:5′-AACGGGCCCTCTAGACTCGAGATGATCCCTAACATAGAAGGAATGG-3′
- LARP7-R:5′-TAGTCCAGTGTGGTGGAATTCATCATATTCAGAAAATCTTATATGTTTACT-3′
- LARP4-F:5′-AACGGGCCCTCTAGACTCGAGATGTTGCTTTTCGTGGAGCAG-3′
- LARP4-R:5′-TAGTCCAGTGTGGTGGAATTCCTTTGGTGATCTGGGTGGCA-3′
- LARP4B-F:5′-AACGGGCCCTCTAGACTCGAGATGACTTCTGATCAGGACGCTAAGG-3′
- LARP4B-R:5′-TAGTCCAGTGTGGTGGAATTCCTGAGGAGACTTGGGGGGAG-3′
- LARP1-F:5′-AACGGGCCCTCTAGACTCGAGATGAAGGAACAGGAGAAAGGAGAA-3′
- LARP1-R:5′-TAGTCCAGTGTGGTGGAATTCCTTTCCCAAAGTCTGTGTGTTCG-3′

#### Cell transfections

Plasmid transfections were carried out by the polyethylenimine (PEI), Lipofectamine 3000 (Invitrogen), and Lipofectamine 2000 (Invitrogen) methods. In the PEI transfection method, 400 μL of DMEM (serum-free medium) and the plasmid were placed in an empty EP tube and PEI was added into the medium. The mixture was incubated for 15 min. Meanwhile, the cell culture medium was replaced with fresh 10% FBS medium. After 15 min, the mixture was added to the cells, and the fresh medium was replaced after 12–16 h. After 36–48 h, the transfection was completed. In the Lipofectamine 3000 transfection method, DMEM (250 μL) was added to two empty EP tubes and Lipofectamine 3000 was added to one of the tubes and mixed for 5 min. The plasmid and P3000 were added in the other tube and then added to the medium containing Lipofectamine 3000, mixed, and allowed to stand for 5 min. Meanwhile, the cell culture medium was replaced with fresh 10% FBS medium. After 5 min, the mixture was added to the cells, and the fresh medium was replaced after 12 h. After 36–48 h, the transfection was completed and the cells were treated. In the Lipofectamine 2000 transfection method, 125 μL of DMEM (serum-free medium) and the siRNA were placed in an empty EP tube.125 μL of DMEM (serum-free medium) and the Lipofectamine 2000 were placed in another empty EP tube then added to the medium containing siRNA. The mixture was incubated for 5 min. Meanwhile, the cell culture medium was replaced with fresh 10% FBS medium. After 5 min, the mixture was added to the cells. After 36–48 h, the transfection was completed.

- HOGA1 siRNA-homo-sense:5′-GGAUAAAUGUUACAUUCUATT-3′
- HOGA1 siRNA-homo-antisense:5′-UAGAAUGUAACAUUUAUCCTT-3′
- LARP7 siRNA-homo-sense:5′-GAAGAAAGGCCGAAUGAAATT-3′
- LARP7 siRNA-homo-antisense:5′-UUUCAUUCGGCCUUUCUUCTT-3′
- ADAM9 siRNA-1-homo-sense: 5′-GGAAGUACCUGUAAGCUUAAA-3′
- ADAM9 siRNA-1-homo-antisense: 5′-UAAGCUUACAGGUACUUCCUU-3′
- ADAM9 siRNA-2-homo-sense: 5′-GUAUGUAUAUUAUGUUAAAUA-3′
- ADAM9 siRNA-2-homo-antisense: 5′-UUUAACAUAAUAUACAUACUA-3′
- FH siRNA-1-homo-sense: 5′-GGAUCAACAAGCUGAUGAAUG-3′
- FH siRNA-1-homo-antisense: 5′-UUCAUCAGCUUGUUGAUCCUU-3′

### Gene silencing

To generate cells stably knocked down for HOGA1 and LARP7, pMKO-HOGA1, or pMKO-LARP7 were transfected into cells, using pCMV-VSVG and pCMV-Gag as packaging plasmids. Twenty-four hours after transfection, the virus supernatant was collected to infect target cells. Puromycin was used to select stable cells for ~ 7 days.

- Human shHOGA1: GATTGGGCTGATTGTTCACAA
- Human shLARP7: CGTGAGTGGAGTGATTGTGAA

### Transwell migration assays

Cell migration assays were performed with 24-well transwells (8-μm pore size, Falcon). In total, 1.5 × 10^5^ transfected cells were suspended in serum-free DMEM medium and added to the upper chamber, and 700μL DMEM with 10% FBS was placed in the lower chamber. After 16 h of incubation, cells on the lower surface of membrane were fixed in 4% paraformaldehyde and stained with crystal violet. Cells in six microscopic fields were counted and photographed.

### PDAC cells proteome

For the proteomic analysis of PDAC cells, cells were lysed in lysis buffer (8 M Urea, 100 mM Tris Hydrochloride, pH 8.0) containing protease and phosphatase Inhibitors (Thermo Scientific) followed by 1 min of sonication (3 s on and 3 s off, amplitude 25%). The lysate was centrifuged at 14,000*g* for 10 min and the supernatant was collected as whole tissue extract. Protein concentration was determined by Bradford protein assay. Extracts from each sample (500 μg protein) was reduced with 10 mM dithiothreitol at 56 °C for 30 min and alkylated with 10 mM iodoacetamide at room temperature (RT) in the dark for additional 30 min. Samples were then digested using the filter-aided proteome preparation (FASP) method with trypsin. Briefly, samples were transferred into a 30kD Microcon filter (Millipore) and centrifuged at 14,000*g* for 20 min. The precipitate in the filter was washed twice by adding 300μL washing buffer (8 M urea in 100 mM Tris, pH 8.0) into the filter and centrifuged at 14,000*g* for 20 min. The precipitate was resuspended in 200μL 100 mM NH4HCO3. Trypsin with a protein-to enzyme ratio of 50:1 (w/w) was added into the filter. Proteins were digested at 37 °C for 16 h. After tryptic digestion, peptides were collected by centrifugation at 14,000*g* for 20 min and dried in a vacuum concentrator (Thermo Scientific). Dried peptides were then used for proteomic analysis.

### Immunoprecipitation

For immunoprecipitation, cells were lysed with 0.5% NP-40 buffer containing 50 mM Tris–HCl (pH 7.5), 150 mM NaCl, 0.3% NONIDET P-40, 1 μg mL − 1 aprotinin, 1 μg mL − 1 leupeptin, 1 μg mL − 1 pepstatin, and 1 mM PMSF. Cell lysates were incubated with Flag beads (Sigma) for 3 h at 4 °C. The binding complexes were washed with 0.5% NP-40 buffer and mixed with loading buffer for SDS-PAGE.

### Glycolytic rate assays

Glycolytic rate assay was performed according to the published procedure with slight modifications [116]. Briefly, PDAC cells (high glucose cultivated PANC-1, high glucose cultivated PANC-1 FH KD, low glucose cultivated PANC-1, low glucose cultivated PANC-1 FH KD, high glucose cultivated SW1990, high glucose cultivated SW1990 FH KD, low glucose cultivated SW1990, low glucose cultivated SW1990 FH KD) (5 × 10^5^) seeded in a 6-well plate were washed once with PBS and incubated in 2 ml of Krebs buffer (126 mM NaCl, 2.5 mM KCl, 25 mM NaHCO3, 1.2 mM NaH2PO4, 1.2 mM MgCl_2_, 2.5 mM CaCl_2_) without glucose for 30 min at 37 °C. The Krebs buffer was removed, and 2 ml of Krebs buffer containing 5.5 mM glucose spiked with 10 μCi of D-[5-3H]-glucose was then added into each well followed by incubation for 1 h at 37 °C. An aliquot of Krebs buffer (50 μl) was mixed with an equal volume of 0.2 N HCl in an uncapped PCR tube, which was then transferred into a 1.5 ml Eppendorf tube containing 0.5 ml of unlabeled distill water. The Eppendorf tubes were sealed to allowed diffusion of 3H_2_O into unlabeled water for 24 h at 37 °C. The amount of diffused 3H_2_O was measured using a scintillation counter and normalized to the cell number counted in each sample.

### Glucose uptake assay

Glucose uptake was measured by a kit obtained from Promega (#J1342) according to the manufacturer’s instructions. Briefly, 10,000 PDAC cells were seeded into 96-well plates and grew for 12 h. The cells were washed twice with PBS and incubated with 1 mM of 2-DG for 10 min at 37 °C. The uptake was terminated by the addition of an acid detergent solution (stop buffer), and neutralization buffer was then added to neutralize the acid. The 2-DG6P detection reagent containing glucose-6-phosphate dehydrogenase, NADP+, reductase, recombinant luciferase, and proluciferin substrate was added to the sample wells. The plate was incubated for 1 h at 25 °C, and luminescence intensity was read on a luminometer with 0.3–1 s integration. The glucose uptake level was normalized according to the cell number counted in a duplicate sample.

### Metabolite measurement by LC–MS/MS

The metabolites were extracted using a previously described protocol [117, 118]. Briefly, the samples were resuspended in HPLC-grade H_2_O before flow injecting and analyzing with the Q Executive HFX mass spectrometer (Thermo Fisher) coupled to a Shimadzu HPLC system (LC 20AB) via Multiple reaction monitoring (MRM) mode to only detect the concentration of pyruvic acid, lactate, and oxaloacetate. The relative concentration of pyruvic acid, lactate, and oxaloacetate could all be analyzed.

### Tandem affinity purification

Cells were transfected with pLVXyu-BirA-HOGA1. Apply biotin (50 μM final concentration) to cells after 12–16 h. After 36–48 h, the transfection was completed and the cells were lysed on ice in 0.1% NP40 buffer containing 50 mM Tris–HCl (pH 7.5), 150 mM NaCl, 0.3% NONIDET P-40, 1 μg mL − 1 aprotinin, 1 μg mL − 1 leupeptin, 1 μg mL − 1 pepstatin, and 1 mM PMSF. After removal of insoluble cell debris by high-speed centrifugation, cell lysates were incubated with SBP beads (Millipore) for 3 h at 4 °C. The precipitates were washed three times with 0.1% NP40 buffer, two times with ddH2O, and three times with 50 mM NH4HCO3. On-bead tryptic digestion was performed at 37 °C overnight. The peptides in the supernatant were collected by centrifugation and dried in a speed vacuum (Eppendorf). Samples were re-dissolved in NH4HCO3 buffer containing 0.1% formic acid and 5% acetonitrile (ACN) before being subjected to mass spectrometry.

### Western blotting

Protein samples were separated using SDS-PAGE. The protein was transferred onto a NC membrane. Then, the membranes were blocked using tris-buffered saline tween (TBST) with 5% skimmed milk at room temperature for 1 h, followed by incubation using corresponding primary antibodies at 4 °C overnight. Next day, the membranes were then incubated in anti-mouse or anti-rabbit IgG at room temperature for 1 h. The protein bands were visualized using an enhanced chemiluminescence protein detection kit (Pierce Biotechnology; Thermo Fisher Scientific, Inc), and then, the signal was quantified by Image J (NIH, Bethesda, MD, USA).

### Quantitative RT-PCR

The Superscript III RT kit (Invitrogen) was used with random 3 hexamer primers to produce cDNA from 4 μg total RNA. ACTIN was used as the endogenous control for samples. All primers for analysis were synthesized by TSINGKE Biological Technology (Shanghai). The analysis was performed by using an Applied Biosystems 7900HT Sequence Detection System, with SYBR green labeling.

- QPCR-LARP1-F: 5′-ACACAAGTGGGTTCCATTACAAA-3′
- QPCR-LARP1-R:5′-CTCCGCGATTGGCAGGTAT-3′
- QPCR-LARP4B-F:5′-GACAAGTTCCATCCCACCTTTG-3′
- QPCR-LARP4B-R:5′-CCATTGGCATCCGATCCCT-3′
- QPCR-LARP4-F:5′-CCACCCCAGTAACTCATGGAA-3′
- QPCR-LARP4-R:5′-AGCTCTGCATTACCCTCAGGA-3′
- QPCR-CDK1-F:5′-GGATGTGCTTATGCAGGATTCC-3′
- QPCR-CDK1-R:5′-CATGTACTGACCAGGAGGGATAG-3′
- QPCR-CCNB1-F:5′-AACTTTCGCCTGAGCCTATTTT-3′
- QPCR-CCNB1-R:5′-TTGGTCTGACTGCTTGCTCTT-3′
- QPCR-CCNB2-F:5′-TTGGCTGGTACAAGTCCACTC-3′
- QPCR-CCNB2-R:5′-TGGGAACTGGTATAAGCATTGTC-3′
- QPCR-HOGA1-F:5′-GGTCCCCAAGTCTGGTCTTCT-3′
- QPCR-HOGA1-R:5′-GATACCCGCAATGTCCACCTT-3′
- QPCR-LARP7-F:5′-GGCTCTGACATTGAGTCCACT-3′
- QPCR-LARP7-R:5′-TGCTTCAACTCTGTCCCGTTT-3′

### Cell proliferation assay

Different groups of cells (2000 cells/well) were seeded into 96-well plates. At the indicated detection times, CCK8 reagent was added into each well. The plates were incubated at 37 °C for 1 h, and then, absorbance of the 96-well plates was detected at a wavelength of 450 nm.

### Ubiquitination assay

pcDNA3.1-Ub-HA and target plasmids were transfected for 24–48 h. MG132 was added for 8 h before harvesting the cells. Cells were next lysed in 1% SDS buffer (Tris–HCl pH 7.5, 0.5 mM EDTA, 1 mM DTT), and boiled for 10 min, and the lysates were diluted 5–tenfold. Flag Beads was added into the lysates for 3 h at 4 °C.

### Flow cytometric analysis

Approximately 10^6^ treated cells were suspended in cold 70% ethanol for 3 h and incubated for 1 h at 37 °C in PBS with DNase-free RNase A (100 mg mL − 1) and propidium iodide (50 mg mL^−1^). The cells were next analyzed using a fluorescence-activated cell sorter (FACS). Data are presented as means ± SD of three independent experiments.

### Xenograft tumorigenesis experiments

Different groups of PANC-1 cells (5 × 10^6^) were re-suspended in PBS and injected subcutaneously (SC) into the right flank of 5-week-old BALB/c-nude mice. The weight and the tumor diameter of each mouse were measured every week. Tumor volume (mm^3^) was calculated as follows: (shortest diameter)^2^ × (longest diameter) × 0.5. Four weeks later all mice were killed.

### Quantification and statistical analysis

Statistical details of experiments and analyses were noted in the figure legends and supplementary tables. Standard statistical tests were used to analyze the association between clinical information and multi-omics data. Student’s t test, Wilcoxon test, one-way ANOVA, and Kruskal–Wallis test were used for continuous data; Fisher’s exact test was used for categorical data. The Benjamini–Hochberg adjusted *P* values of differentially expressed RNA/proteins/phosphoproteins were calculated. Log-rank tests and Kaplan–Meier survival curves were used to compare the overall survival and disease free survival. All statistical tests were two-sided, and statistical significance was considered when *p* < 0.05. Variables associated with survival were identified using univariate Cox proportional hazards regression models. The correlation between two sets of data was calculated using Spearman’s correlation. All the analyses of clinical data were performed in R (version 4.0.2) and GraphPad Prism 8. For functional experiments, each was repeated at least three times independently, and results were expressed as mean ± standard error of the mean (SEM). Statistical analysis was performed using GraphPad Prism 8.

## Supplementary Information

**Additional file 1: Fig. S1**. Quality assessment of proteomic data of PDAC, related to Fig. 1. **A-D.** The number of identified mRNA (A), protein (B), phosphoprotein (C) and phosphosite (D) in the tumors and NATs. **E** Trinucleotide motif frequency plots and mutations frequency similarity of identified five mutational profiles (Fudan cohort). **F** Trinucleotide motif frequency plots and mutations frequency similarity of identified five mutational profiles (CPTAC cohort). **G** Spearman’s correlation analysis of 16 HEK293T cell samples as MS quality control to evaluate the robustness of label-free quantification (Spearman’s correlation coefficients, 0.88-0.98). **H** The cumulative identified proteins in the 220 NATs (blue) and 226 tumors (red). **I** Distribution of log10-transformed FOT abundance of identified proteins in 226 tumors and 220 NATs that passed quality control. Red presents tumor samples (n = 226), blue denotes NAT samples (n = 220). In the box plots, the middle bar represents the median, and the box represents the interquartile range; bars extend to 1.5× the interquartile range. **J** Proteomic datasets filtered at different levels for various statistical analyses. 16,584 gene products (GPs) identified in 446 PDAC samples; 16,567 GPs: at 1% protein level FDR; 12,602 GPs: at high abundance range (FOT ≥ 1E−5) and discard KRTs; 7,055 GPs: GPs identified in at least 38 tumor samples or 37 NAT samples. **K** Venn diagram summary of the number of identified mRNA (19,992), proteins (16,584), phosphoproteins (7,399). **L** PCA analysis of proteomic data based on years of sample collection (left) and experimental batches (right)..

**Additional file 2: Fig. S2**. Phosphoproteomic characterization, related to Fig. 1. **A.** MS2 spectrums of the YAP1 at S109 and RB1 at S807. **B.** IHC staining of YAP1 at S109 and RB1 at S807 in PDAC tumor tissues and NATs. **C.** Boxplot indicting the phosphosite expression of YAP1_pS109 and RB1_pS807 between tumors and NATs.

**Additional file 3: Fig. S3**. The impacts of somatic copy number alterations in PDAC cohort, related to Fig. 2. **A** Bar plots showing pathway enrichment of *cis*/*trans*-effect between CNA and protein (left) or phosphoprotein (right). **B** Distribution of *IRF6* status between the younger patients’ group (≤ 60) and the older patients’ group (> 60) (left). The boxplot reveals the comparison of the mRNA (middle) and protein (right) abundance of IRF6 between the younger patients (≤ 60) and the older patients (> 60) (Wilcoxon test). **C** Kaplan-Meier curves for overall survival based on IRF6 abundance in the younger patients (left) or the older patients (right) (log-rank test). **D** The heatmap indicating the protein abundance of genes participated in cell cycle in the four groups (*TP53* mut, younger patients; WT, younger patients; *TP53* mut, older patients; WT, older patients). Each column represents a sample. **E** Spearman-rank correlation of the abundance of CDK4 and phosphosites. Cell cycle and cell proliferation associated phosphosites are labeled in pink. **** *p* < 1.0E-4, *** *p* < 1.0E-3, ** *p* < 1.0E-2, * *p* < 0.05, ns > 0.05.

**Additional file 4: Fig. S4**. Characteristics of PDAC patients with *KRAS*^*G12D*^ mutations.** A **The volcano plot showing the phosphosites that significantly altered between tumors with *KRAS*^*G12D*^ and with other types of *KRAS* mutations. **B** The heatmap indicating the phosphorylation abundance of RAF1, MAP2K2, MAPK1 across tumors with different *KRAS* mutations. **C** Kaplan-Meier curves for overall survival based on the phosphorylation of E2F1 at S364 (left) and based on the TF activity of E2F1 (right). **D** The box plots indicating the inferred TF activity of E2F1 (right), phosphorylation of E2F1 at S364 (middle) and the protein expression of E2F1 (left) across the tumors with diverse *KRAS* mutations. **E** IHC staining of E2F1_pS364 and E2F1 in tumors with different *KRAS* mutations. **F** The scatter plots indicating correlation between protein expression of E2F1 (green) and phosphorylation of E2F1 at S364 (red) with the TF activity of E2F1. **G** The heatmap revealing the expression patterns of E2F1’s TGs across the PDAC patients. Color of each cell shows the z scored FPKM of the mRNAs. **H** The scatter plot showing significance of correlation between proteins with their cognate mRNAs (y axis), versus the significance of protein’s Hazard ratio (x axis). Proteins negatively associated with patients’ overall survival are color coded in red. **I** The systematic diagram summarizing that the phosphorylation of E2F1 at S364 enhanced the cell proliferation process and led to poor prognosis in tumors with *KRAS*^*G12D*^ mutations. **** *p* < 1.0E-4, *** *p* < 1.0E-3, ** *p* < 1.0E-2, * *p* < 0.05, ns > 0.05.

**Additional file 5: Fig. S5**. Integrated multi-omics features in tumor tissues compared with NATs of the PDAC, related to Fig. 3.** A **Principal component analysis (PCA) of RNA-Seq (19,992 genes) in 54 tumors and 51 NATs. Red, tumors; blue, NATs. **B** PCA of 7,399 phosphoproteins in 113 paired samples. Red, tumors; blue, NATs.** C **A volcano plot showing the results of a two-tailed Student’s t test comparing tumors and NATs at transcriptome level. **D** A volcano plot showing the results of a two-tailed Student’s t test comparing tumors and NATs at phosphoproteome level. **E–G. **KEGG pathway analysis of differentially expressed mRNAs (E), proteins (F) and phosphoproteins (G) revealing pathways that were significantly enriched in tumors and NATs.** H**, **I** Gene-wise correlations of mRNA and protein expression in NATs (H) and tumors (I). **J**, **K** Gene-wise correlations of phosphosites and protein expression in NATs (J) and tumors (K). **** *p* < 1.0E-4, *** *p* < 1.0E-3, ** *p* < 1.0E-2, * *p* < 0.05, ns > 0.05.

**Additional file 6: Fig. S6**. Proteomic features in tumor tissues compared with NATs of the PDAC in CPTAC cohort, related to Fig. 3. **A** Venn diagram summary of the number of up-regulated (left) and down-regulated (right) proteins between Fudan cohort and CPTAC cohort. **B** Differentially expressed proteins in tumors and NATs in CPTAC cohort (right panel). The left panel shows KEGG pathway analysis of identified differentially expressed proteins. Red, up-regulated pathways; blue, down-regulated pathways. **C** Differential expression of the pancreas-specific proteins in tumors and NATs (list from The Human Protein Atlas). Red, pancreas enriched proteins; green, pancreas enhanced protein; blue, pancreas grouped protein (left panel). The right panel shows KEGG pathway analysis of identified pancreas-specific proteins. **D** The expression of pancreas signature proteins in tumors and NATs in CPTAC cohort. **E** Heatmap (left) showing the fold change of 15 potential targets between tumors and NATs in CPTAC cohort. Scatter plot (right) shows the Cox regression coefficient of these proteins. Name in red indicates FDA-approved drugs. **** *p* < 1.0E-4, *** *p* < 1.0E-3, ** *p* < 1.0E-2, * *p* < 0.05, ns > 0.05.

**Additional file 7: Fig. S7**. The expression of phosphotyrosine in PDAC cohort, related to Fig. 3. **A.** The proportion of phosphorylation sites S, T and Y in tumors and NATs, respectively. **B.** The comparison of the percentage of phosphorylation sites S, T and Y in Fudan cohort and other gastrointestinal tumor cohorts. **C.** The scatter plot demonstrating the T/NAT ratio of phosphotyrosine (y axis) and T/NAT ratio of their corresponding proteins (x axis). **D.** Heatmap showing the expression of altered proteins and their corresponding phosphotyrosine. Significance (survival) of proteins which were negatively correlated with prognosis are labeled. **E.** Boxplot of PLEC (left) and PLEC/Y4611 (right) expression between metastasis/non-metastasis patients (Wilcoxon test). **** *p* < 1.0E-4, *** *p* < 1.0E-3, ** *p* < 1.0E-2, * *p* < 0.05, ns > 0.05.

**Additional file 8: Fig. S8**. The effects of diabetes on the proteogenomic characteristics of PDAC, related to Fig. 4. **A** The boxplot representing the comparison of blood glucose concentration between patients with diabetes and without diabetes. **B** The Kaplan-Meier curves for overall survival based on diabetic patients with or without medical treatment. **C** The heatmap showing the distribution of *10q25.3* deletion and *MTOR* mutations across different samples in TCGA cohort. **D** The bar plot showing the distribution of patients with *10q25.3* deletion between patients with diabetes and without diabetes in WSU cohort. **E** The scatter plot depicting the *cis*-effect of genes located in *10q25.3* between CNA and RNA (left) or RNA and protein (right). **F** The boxplot indicating the protein expression of AKT1 between patients with and without diabetes history (Wilcoxon test). **G** The expression heatmap describing the phospho-substrates of MTOR downregulated by *MTOR* mutations. **H** The scatter plot depicting the correlation between abundance of EIF4EBP1_pT37 and GSVA score (Spearman’s correlation). **I** The boxplots indicating the protein expression of AKT1S1, EIF4E3, EIF4EBP1, and RPTOR between patients with and without diabetes history (Wilcoxon test). **J** Spearman-rank correlation of the protein expression of AKT1S1, EIF4E3, EIF4EBP1, and RPTOR versus the GSVA score of translational initiation. **** *p* < 1.0E-4, *** *p* < 1.0E-3, ** *p* < 1.0E-2, * *p* < 0.05, ns > 0.05.

**Additional file 9: Fig. S9**. *8p11.22* amplification is associated with PDAC metastasis, related to Fig. 5. **A** Western blot assays were conducted for detecting ADAM9 protein in PANC-1 cells after si-ADAM9-1 and si-ADMA9-2 knocked down. **B** The scatter plot depicting the correlation between ADAM9 expression and expression of ADAM9-interacting proteins (Spearman’s correlation). **C** The scatter plot depicting the correlation between the abundance of SRC and pathways (GSVA score). **D** The boxplots indicating the protein expression of ADAM9 (left) and CDCP1 (right) between tumor tissues and NATs in CPTAC cohort (Student’s t test). **E** Spearman-rank correlation of ADAM9 and CDCP1 protein expression (left). The scatter plot on the right depicting the correlation of CDCP1 and SRC protein expression in CPTAC cohort (Spearman’s correlation). **F** Spearman-rank correlation of the abundance of ADAM9 and epithelial cell migration (GSVA score) in CPTAC cohort (Spearman’s correlation). **G** Heatmap of the relative abundance of epithelial-cell-migration-related proteins that are significantly associated with ADAM9 expression in CPTAC cohort. **H** WGCNA of 93 PDAC samples depicting module eigengenes (MEs) highly correlated with primary tumor in the patients with five metastasis sites (upper heatmap of the left panel). Enrichment analysis for the different MEs is presented in the lower heatmap of the left panel (*P* value < 0.05). The heatmap (right) shows the differential proteins involved the overrepresented pathways of different modules corresponding to the five metastasis sites. **I** Protein-regulatory network showing proteins participated in metastasis-sites-associated pathways. **J** Boxplot of ADAM9 protein expression among five metastasis sites (Kruskal-Wallis test). **** *p* < 1.0E-4, *** *p* < 1.0E-3, ** *p* < 1.0E-2, * *p* < 0.05, ns > 0.05.

**Additional file 10: Fig. S10**. Protein expression changes in PANC-1 cell lines after *ADAM9* knocked down and the co-expressed proteins of ADAM9, related to Fig. 5. **A** The schematic work flow of validation experiments for the protein expression changes after *ADAM9* knocked down. **B** The volcano plot showed the proteins that significantly altered between PANC-1 cells with *ADAM9* KD and Scrambled siRNA. The GO processes enriched by proteins downregulated in *ADAM9* KD cell lines are noted on the left. **C** Expression of proteins participated in collagen-containing extracellular matrix, WNT signaling pathway and cell migration involved in angiogenesis in the *ADAM9* KD group and Scrambled siRNA group (n = 3 repeats per group). **D** The schematic work flow of co-immunoprecipitation assay of *ADAM9* KD group and Scrambled siRNA group in PANC-1 cell lines. **E** Table showing GO processes enriched by the proteins interact with ADAM9. **F** Network of proteins interacting with ADAM9.

**Additional file 11: Fig. S11**. Multi-omics subtypes of PDAC patients, related to Fig. 6. **A** Consensus matrices of identified clusters (k = 2 to 4) of transcriptomic, proteomic, and phosphoproteomic subtypes. **B** The consensus CDF and delta area (change in CDF area) plots, as well as the silhouette plots, are shown. **C** Heatmaps showing the comparison between transcriptomic and phosphoproteomic subtypes (columns) with proteomic subtypes (rows). Each row sums to one, with different entries showing the proportion of tumors allocated to proteomic subtypes. **D** The boxplots of overrepresented proteins (One-way ANOVA) and the association of the proteins with prognosis (log-rank test) among three proteomic subtypes. **E** The Sankey plot revealing the association between our proteomic subtypes and Collisson subgroups. **F** Kaplan-Meier plot comparing the survival outcomes between Collisson QM-PDA samples assigned into two proteomic clusters (S-II and S-III). **G** Differential expression protein between S-II and S-III included in Collisson QM-PDA samples. **H** Bubble diagram revealing the enriched pathway of upregulated proteins in S-III included in Collisson QM-PDA samples. **I** Kaplan-Meier curves for overall survival of patients with tumor located on head (H-PDAC) and with tumor located on body-tail (BT-PDAC) in Fudan cohort (left) and CPTAC cohort (right) (log-rank test). **J** The heatmap indicating the GSVA scores of the pathway at proteomic level (upper) and proteins enriched in cell cycle and ERBB signaling pathway (lower) between H-PDAC and BT-PDAC in CPTAC cohort. **** *p* < 1.0E-4, *** *p* < 1.0E-3, ** *p* < 1.0E-2, * *p* < 0.05, ns > 0.05.

**Additional file 12: Fig. S12**. Multi-omics characteristics of the three proteomic subtypes, related to Fig. 6. **A** Bar plot showing the number of overexpressed molecules in each proteomic subtypes at both proteome and transcriptome level. **B** Heatmap of the characteristic molecules of each proteomic subtypes at proteome (left) and transcriptome (right) level. Molecules previously reported to be associated with PDAC are marked in red. **C** Scatter plot displaying the significance of molecules that highest expressed in S-III at RNA level (y axis) and at protein level (x axis). Enriched biological pathways are showed on the right. **D** The systematic diagram summarizing significantly altered proteins that enriched in ErbB_EGFR signaling pathway. Color of each cell shows Z-scored average abundance of the protein among proteome subtypes. **E** The kinome tree of three proteomic subtypes. **F** Coxcomb diagrams showing the distribution of 10 kinase family in three proteomic subtypes. **G** The association of kinase-substrate pairs overrepresented in proteomic subtypes. **** *p* < 1.0E-4, *** *p* < 1.0E-3, ** *p* < 1.0E-2, * *p* < 0.05, ns > 0.05.

**Additional file 13: Fig. S13**. Proteomic subtypes divided early-stage PDAC patients into subtypes with different prognosis, related to Fig. 6. **A** Summary of the relationship between proteomic subtypes and TNM stage subtypes. **B** Kaplan-Meier curves for overall survival of early-stage (IA and IB) PDAC patients based on 3 proteomic subtypes (log-rank test). **C** Bar plot indicating the enriched pathways in early-stage patients who were divided into three proteomic subtypes. **D** Heatmap of differentially expressed kinase signatures among the three proteomic subtypes in patients with early-stage PDAC. **** *p* < 1.0E-4, *** *p* < 1.0E-3, ** *p* < 1.0E-2, * *p* < 0.05, ns > 0.05.

**Additional file 14: Fig. S14**. Proteomic subtypes of CPTAC PDAC patients based on Fudan’s proteomic stratification, related to Fig. 6. **A** Consensus matrices of identified clusters (k = 2 to 5) of proteomic subtypes in CPTAC cohort based on Fudan’s proteomic stratification. **B**, **C** The consensus CDF (B) and delta area (change in CDF area) plots (C) are shown. **D** The heatmap depicting the relative abundance of signature proteins among three proteomic subtypes. **E** The association of three CPTAC proteomic subtypes based on our proteomic stratification with clinical outcomes. **F** Representative GO terms in CPTAC proteomic subtypes based on our proteomic stratification. **G**, **H** The boxplots indicating the protein expression of GRB7 (G), AKT2 and ERBB2 (H) among three proteomic subtypes in CPTAC cohort based on our proteomic stratification (Kruskal-Wallis test). **I** A volcano plot showing the prognosis associated characteristic proteins of three subgroups in CPTAC cohort based on our proteomic stratification. Red, proteins that negatively associated with prognosis in Fudan cohort; green, proteins that positively associated with prognosis in Fudan cohort. **J** The Sankey plot revealing the association between CPTAC subgroups and CPTAC subgroups based on our proteomic stratification. **** *p* < 1.0E-4, *** *p* < 1.0E-3, ** *p* < 1.0E-2, * *p* < 0.05, ns > 0.05.

**Additional file 15: Fig. S15**. Protein markers for three PDAC proteomic subtypes, related to Fig. 6. **A** The boxplot indicating the comparison of tumor purity across the proteomic subtypes. **B** Heatmap indicating the expression of protein biomarkers in the three proteomic subtypes. **C** Kaplan-Meier curves for overall survival based on proteomic abundance of ACOX1, S100A4 and MCM6 (log-rank test). **D** IHC profiling of proteomic subtype markers in PDAC. FFPE sections were stained for ACOX1, S100A4 and MCM6 protein markers in PDAC tumor tissues. The scale bar indicates 100 μm. **** *p* < 1.0E-4, *** *p* < 1.0E-3, ** *p* < 1.0E-2, * *p* < 0.05, ns > 0.05.

**Additional file 16: Fig. S16**. Characterization of immune infiltration in PDAC, related to Fig. 7. **A** Boxplot showing the age among five immune clusters. **B** Heatmap of xCell score between samples in old/young group of TCGA cohort. **C** Distribution of the percentage of proteomics subtypes across different immune subtypes (Fisher’s exact test). **D** The Sankey plot revealing the association between proteomics subtypes and immune subtypes. **E** Kaplan-Meier curves for overall survival based on immune subtypes in S-II (log-rank test). **F** Heatmap illustrating cell type compositions and pathways across 5 immune clusters. **G** Consensus matrices of identified clusters (k = 3 and 5) of immune subtypes. **H** Bar plots of GSVA scores among 5 immune subtypes. **** *p* < 1.0E-4, *** *p* < 1.0E-3, ** *p* < 1.0E-2, * *p* < 0.05, ns > 0.05.

**Additional file 17: Fig. S17**. The impact of *FH* amplification in PDAC, related to Fig. 7. **A** Scatter plot on the left presents the correlation between *FH* copy number and FH expression in TCGA cohort (left) or in WSU cohort (right). **B**, **C** The bar plot shows the distribution of patients with *FH* amplification between diabetes and non-diabetes in our cohort (B), in TCGA cohort (C, left) and in WSU cohort (C, right). **D** The schematic work flow of our validation experiments for the *FH* in prompting PDAC tumor cell proliferation through regulating glucose metabolism. **E** Proliferation of PDAC cells (SW1990: left; PANC1: right) associated with various treatments (n = 4 repeats per group). **F** The comparison of glycolytic rates, glucose consumption, oxaloacetate and lactate production associated with various treatments (n = 4 repeats per group). **G** The schematic work flow of our validation experiments for the impacts of *FH* on tumor immune microenvironments. **H** The heatmap showing the expression patterns of cytokines detected in supernatants of cell cultures across cells under different treatments (n = 3 repeats per group). The schematic diagrams on the right shows the impacts of cytokines such as IL12, TNF in transforming monocytes to cDCs (up); the impacts of cytokines including CCL7, CCL3 in recruiting monocytes (bottom). **** *p* < 1.0E-4, *** *p* < 1.0E-3, ** *p* < 1.0E-2, * *p* < 0.05, ns > 0.05.

**Additional file 18: Fig. S18**. Characterization of ECM subtypes in PDAC, related to Fig. 7. **A** H&E-stained slides of samples of deserted, inter, reactive ECM subtypes. **B** The expression heatmap describing differentially expressed proteins and phosphoproteins among three ECM subtypes. The enriched biological pathways were annotated on the right. **C** Boxplot illustrating the purity of sample among ECM subtypes. **D** Boxplots showing the expression of ECM markers among ECM subtypes. **E** IHC profiling of ECM markers in ECM subtypes. **F** Heatmap of drug targets of deserted, inter, reactive ECM subtypes. Drugs are labeled on the left. **G** Boxplot illustrating the purity of sample among 5 immune subtypes. **H** The Sankey plot revealing the association between ECM subtypes and our immune subtypes. **I** The association of five immune clusters with clinical outcomes in reactive ECM subtype samples. **J** Heatmap showing the xCell signatures among five immune clusters in reactive ECM subtype samples. **** *p* < 1.0E-4, *** *p* < 1.0E-3, ** *p* < 1.0E-2, * *p* < 0.05, ns > 0.05.

**Additional file 19: Fig. S19**. Biological diversity between high and low purity samples. **A** The heatmap indicating the correlation between histological purity and purity assessed by ESTIMATE and ABSOLUTE package. **B** The expression heatmap describing differentially expressed proteins and phosphoproteins in high/low tumor purity. The enriched biological pathways are annotated on the right.

**Additional file 20: Fig. S20**. HOGA1 regulated LARP7 expression, related to Fig. 8. **A** The association of the mRNA expression of HOGA1with prognosis (log-rank test). **B** Boxplot showing the differential expression of HOGA1 between tumors and NATs in CPTAC cohort (Student’s t test). **C** Expression of HOGA1, LARP7, CDK1, CCNB1, and CCNB2, in tumor tissues were detected by western blot analysis. N = non-tumor adjacent tissue, T = tumor tissue. **D** The impacts of HOGA1 overexpression on PANC-1 and BxPC-3 cells proliferation. **E** Proliferation of PANC-1 and BxPC-3 cells associated with various treatments (n = 4 repeats per group). **F** Cellular localization of HOGA1 in PANC-1 cells. **G** GO term category of HOGA1 interacting proteins. **H**–**I** LARP7 mRNA levels in PANC-1 cells with various treatments. **J** The percentage of G2/M phase cells in PANC-1 cells (n = 5 repeats per group). **** *p* < 1.0E-4, *** *p* < 1.0E-3, ** *p* < 1.0E-2, * *p* < 0.05, ns > 0.05.

**Additional file 21: Fig. S21**. Protein expression changes in PANC-1 cell lines after *HOGA1* knocked down and over expressed, related to Fig. 8. **A** The schematic work flow of validation experiments for the protein expression changes after *HOGA1* knocked down and over expressed. **B** The volcano plot showing the proteins that significantly altered between PANC-1 cells with *HOGA1* KD (left)/HOGA1 OE (right) and WT. The GO processes enriched by proteins upregulated in *HOGA1* KD cell lines and downregulated in *HOGA1* OE cell lines compared with WT are noted on the bottom. **C** Expression of proteins involved in cell cycle in the *HOGA1* OE group, *HOGA1* KD group and WT group. (n = 3 repeats per group).

**Additional file 22: Fig. S22**. Hematoxylin and eosin (H&E) staining on PDACs. The tumor cell purities of tumor tissues and the non-tumor cell purities of tumor-adjacent tissues.

**Additional file 23: Table S1**. Clinicopathologic Information and Multi-omics Data in PDAC Cohort. Table S1A Multi-omics information of PDAC cohort. Table S1B Clinicopathologic information of the 229 PDAC.

**Additional file 24: Table S2**. The Impacts of Somatic Copy Number Alterations in PDAC Cohort. Table S2A Matrix of amplification peaks, followed by the genes contained in them, 95% confidence level. Table S2B Matrix of deletion peaks, followed by the genes contained in them, 95% confidence level. Table S2C Matrix of the top 10 mutated genes in PDAC of Fudan cohort. Table S2D Matrix of the 10 mutated genes used in Figure 1C of ICGC, 2012 cohort. Table S2E Matrix of the 10 mutated genes used in Figure 1C of ICGC, 2016 cohort. Table S2F Matrix of the 10 mutated genes used in Figure 1C of TCGA, 2018 cohort. Table S2G Matrix of the 10 mutated genes used in Figure 1C of UTSW, 2015 cohort. Table S2H Matrix of the 10 mutated genes used in Figure 1C of CPTAC, 2021 cohort.

**Additional file 25: Table S3**. Integrated Multi-omics Features in Tumor Tissues Compared with NATs of the PDAC. Table S3A Matrix of 7,055 protein groups and their relative intensities in at least 1/6 of the samples (226 tumors and 220 NATs). Table S3B 19,992 mRNA and their ratio of tumors/NATs (54 tumors and 51 NATs). Table S3C A list of protein identified both in protein and mRNA level. Table S3D A list of phosphosites used for KSEA analyses.

**Additional file 26: Table S4**. The Effects of Diabetes on the Proteogenomic Characteristics of PDAC. Table S4A Mutations and arm events information in PDAC patients with or without diabetes history. Table S4B Matrix of differential pathway enrichment in patients with or without diabetes history at mRNA level. Table S4C Matrix of differential pathway enrichment in patients with or without diabetes history at protein level.

**Additional file 27: Table S5**. *8p11.22* Amplification Associated with PDAC Metastasis. Table S5A The matrix describing mutations in PDAC metastatic patients and non-metastatic patients. Table S5B Matrix of GSVA scores of pathways significantly altered in PDAC metastatic patients and non-metastatic patients, proteome level. Table S5C Matrix of xCell signatures significantly altered in PDAC metastatic patients and non-metastatic patients, transcriptome level. Table S5D Matrix describing the *cis*-effects of genes located on chromosome *8p11.22*. Table S5E Matrix describing the expression of ADAM9-interacting proteins in PDAC metastatic patients and non-metastatic patients. Table S5F Relative abundance of WNT-signaling-related proteins and phosphosite. Table S5G MS matrix of protein groups and their relative intensities in Scrambled siRNA and *ADAM9* KD groups. Table S5H List of interacting proteins of ADAM9 identified by immunoprecipitation assay. Table S5I WGCNA analysis showing module-trait relationships in PDAC patients with five different metastasis sites. Table S5J Protein expression in metastatic site-specific modules.

**Additional file 28: Table S6**. Proteomic Subtypes of PDAC Patients. Table S6A Proteomic subtypes, transcriptomic subtypes and phosphoproteomic subtypes of PDAC patients. Table S6B A list of differentially expressed proteins among three subtypes. Table S6C Pathway alterations in the proteomic subtypes of the PDAC cohort. Table S6D Matrix of protein expression and kinase activity of kinase activated in S-III. Table S6E Matrix of differential pathway enrichment between patients located in H-PDAC and BT-PDAC. Table S6F Matrix of differentially expressed proteins between patients located in H-PDAC and BT-PDAC.

**Additional file 29: Table S7**. Characterization of Immune Infiltration in PDAC. Table S7A Matrix of xCell signatures significantly altered in 5 immune subgroups. Table S7B Matrix describing the expression of proteins in 5 immune subgroups. Table S7C Matrix of GSVA scores of pathways significantly altered in 5 immune subgroups, transcriptome level. Table S7D Matrix of amplification/deletion events of genes in 5 immune subgroups. Table S7E Matrix describing the expression of proteins involved in TCA cycle.

**Additional file 30: Table S8**. HOGA1 inactivation promotes pancreatic cancer growth through activating LARP7-CDK1 pathway. Table S8A Quantified western blot results of 12 pairs of samples. Table S8B Proliferation of PANC-1 and BxPC-3 cells associated with *HOGA1* knocked down. Table S8C Proliferation of PANC-1 and BxPC-3 cells associated with *HOGA1* overexpressed. Table S8D Proliferation of PANC-1 and BxPC-3 cells associated with metabolic enzyme treatment. Table S8E A list of 773 different proteins detected in PANC-1 cells. Table S8F GO term category of HOGA1 interacting proteins in cultured PANC-1 cells. Table S8G LARP7 mRNA levels in PANC-1 cells with various treatments. Table S8H The percentage of G2/M phase cells in PANC-1 cells. Table S8I Tumor weight of indicated PANC-1 cells subcutaneously injected into nude mice. Table S8J MS matrix of protein groups and their relative intensities in WT, *HOGA1* KD and *HOGA1* OE groups. Table S8K GO term category of proteins upregulated in *HOGA1* KD group and downregulated in *HOGA1* OE group.

## Data Availability

All proteomic and phosphoproteomic raw data have been uploaded to the iProx Consortium (https://www.iprox.org/) with the subproject ID (IPX0002796002 and IPX0002796001, respectively). All the WES raw data have been deposited at NODE (https://www.biosino.org/node/) with accession number (OEP001784). All the RNA-Seq raw data have been deposited at GSA (https://ngdc.cncb.ac.cn/gsa/) with the accession number (HRA002195).

## Abbreviations

ACN: Acetonitrile
AGC: Automatic gain control
BT-PDAC: Pancreatic body-tail
CAG: Cancer associate gene
CAGs: Cancer-associated genes
CDF: Cumulative distribution function
CNA: Copy number alteration
DFS: Disease free survival
DMEM: Dulbecco’s modified Eagle’s medium
EOPC: Early-onset pancreatic cancer
FACS: Fluorescence-activated cell sorter
FBS: Fetal bovine serum
FDR: False discovery rate
FFPE: Formalin-fixed paraffin-embedded
FOT: Fraction of total
FPKM: Fragments Per Kilobase of transcript per Million mapped reads
GO: Gene Ontology
GSVA: Gene set variation analysis
H-PDAC: Pancreatic head
HCD: Higher-energy collisional dissociation
H&E: Hematoxylin and eosin
HR: Hazard ratio
HPLC: High-performance liquid chromatography
iBAQ: Intensity-based absolute quantification
ICGC: International Cancer Genome Consortium
IHC: Immunohistochemistry
KSEA: Kinase–substrate enrichment analysis
MGPS: Multi-gene proliferation score
MS: Mass spectrometry
NATs: Non-cancerous adjacent tissues
NMF: Nonnegative matrix factorization
OS: Overall survival
PBS: Phosphate-buffered saline
PC: Pancreatic carcinoma
PCA: Principal component analysis
PCR: Polymerase chain reaction
PDAC: Pancreatic ductal adenocarcinoma cancer
PSM: Peptide-spectrum match
PTM: Post-translational modification
QC: Quality Control
SEM: Standard error of the mean
SMGs: Significantly mutated genes
SNV: Single nucleotide variant
TAM: Tumor-associated macrophage
TBST: Tris-buffered saline tween
TCGA: The Cancer Genome Atlas
TF: Transcription factor
TG: Target gene
TME: Tumor microenvironment
UTSW: UT Southwestern Medical Center
WES: Whole-exome sequencing
WGCNA: Weighted gene correlation network analysis
