# Supplementary figures and images for: Proteogenomic insights into the biology and treatment of pancreatic ductal adenocarcinoma
Source: J Hematol Oncol. 2022 Nov 25;15:168. doi: 10.1186/s13045-022-01384-3 (PMC9701038; doi:10.1186/s13045-022-01384-3)

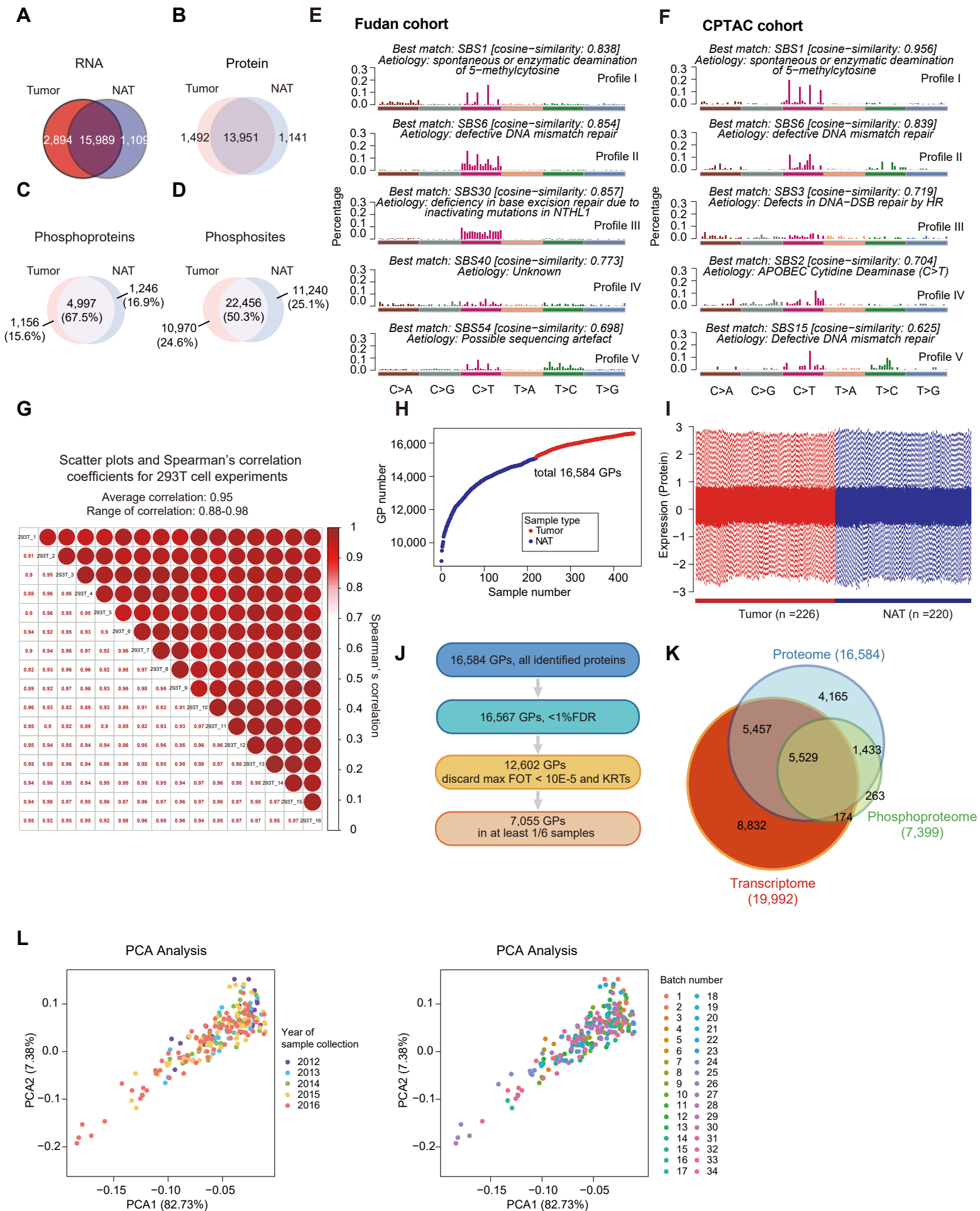

Supplement: Supplementary file 1 — Additional file 1: Fig. S1. Quality assessment of proteomic data of PDAC, related to Fig. 1. A-D. The number of identified mRNA (A), protein (B), phosphoprotein (C) and phosphosite (D) in the tumors and NATs. E Trinucleotide motif frequency plots and mutations frequency similarity of identified five mutational profiles (Fudan cohort). F Trinucleotide motif frequency plots and mutations frequency similarity of identified five mutational profiles (CPTAC cohort). G Spearman’s correlation analysis of 16 HEK293T cell samples as MS quality control to evaluate the robustness of label-free quantification (Spearman’s correlation coefficients, 0.88-0.98). H The cumulative identified proteins in the 220 NATs (blue) and 226 tumors (red). I Distribution of log10-transformed FOT abundance of identified proteins in 226 tumors and 220 NATs that passed quality control. Red presents tumor samples (n = 226), blue denotes NAT samples (n = 220). In the box plots, the middle bar represents the median, and the box represents the interquartile range; bars extend to 1.5× the interquartile range. J Proteomic datasets filtered at different levels for various statistical analyses. 16,584 gene products (GPs) identified in 446 PDAC samples; 16,567 GPs: at 1% protein level FDR; 12,602 GPs: at high abundance range (FOT ≥ 1E−5) and discard KRTs; 7,055 GPs: GPs identified in at least 38 tumor samples or 37 NAT samples. K Venn diagram summary of the number of identified mRNA (19,992), proteins (16,584), phosphoproteins (7,399). L PCA analysis of proteomic data based on years of sample collection (left) and experimental batches (right).. [file 13045_2022_1384_MOESM1_ESM.pdf]

**A**

Protein: YAP1 Sequence: QASTDAGTAGALTPQHVR, S109-Phos

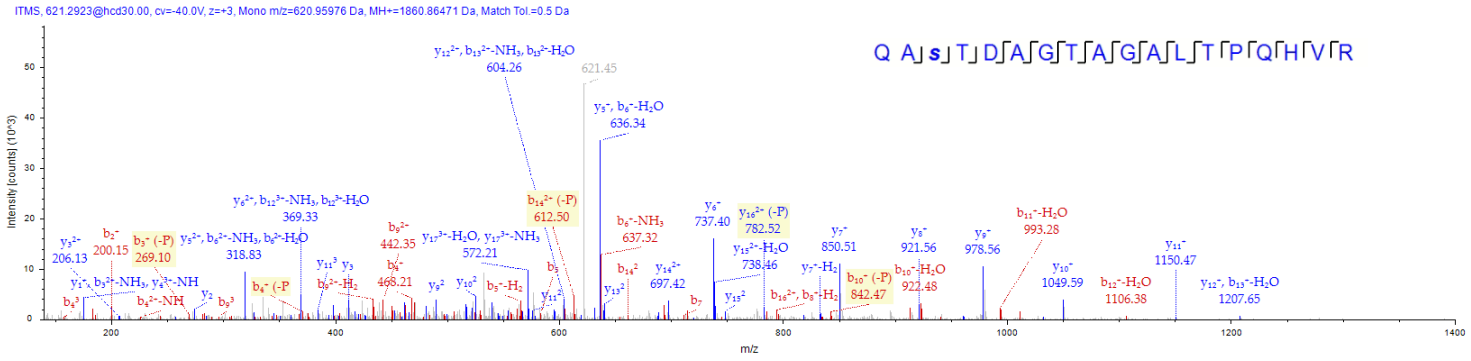

Protein: RB1 Sequence: IPGGNIYISPLK, S807-Phos

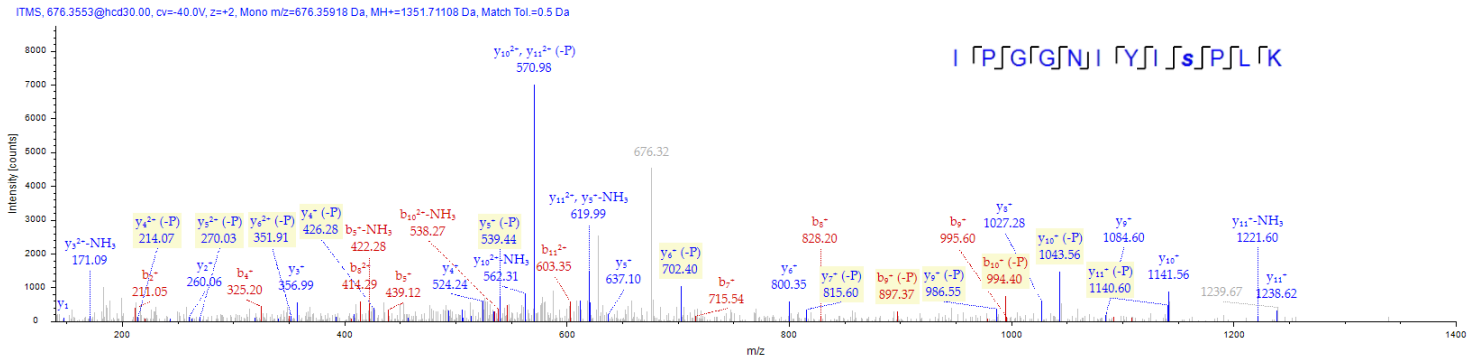

**B**

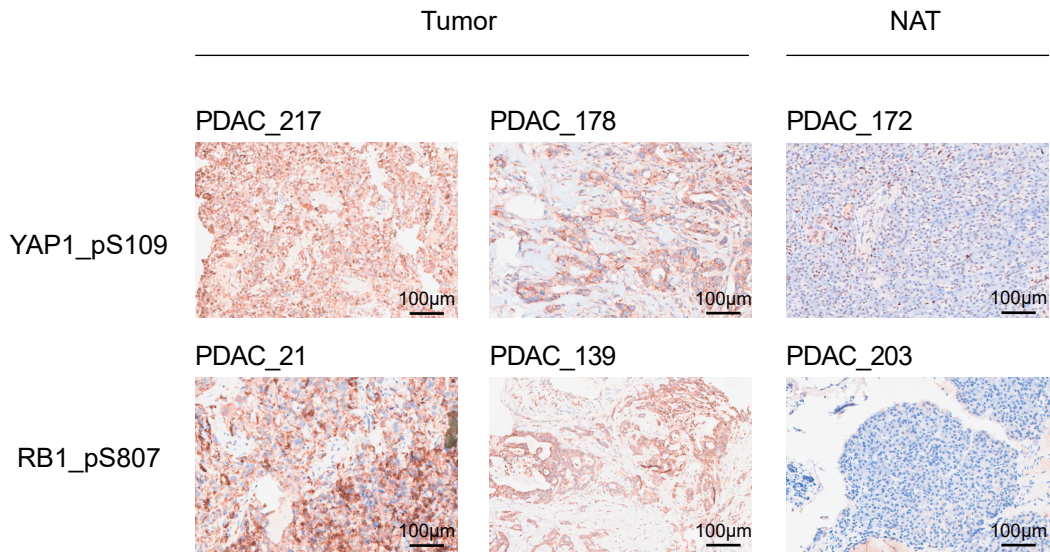

**C**

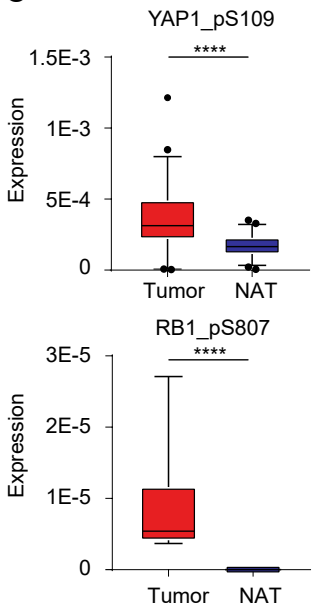

Supplement: Supplementary file 2 — Additional file 2: Fig. S2. Phosphoproteomic characterization, related to Fig. 1. A. MS2 spectrums of the YAP1 at S109 and RB1 at S807. B. IHC staining of YAP1 at S109 and RB1 at S807 in PDAC tumor tissues and NATs. C. Boxplot indicting the phosphosite expression of YAP1_pS109 and RB1_pS807 between tumors and NATs. [file 13045_2022_1384_MOESM2_ESM.pdf]

**A**

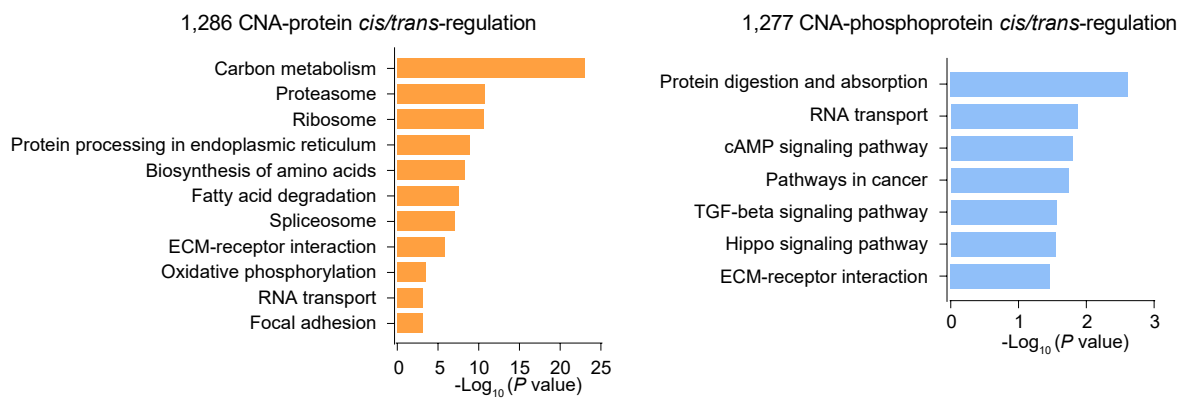

**B**

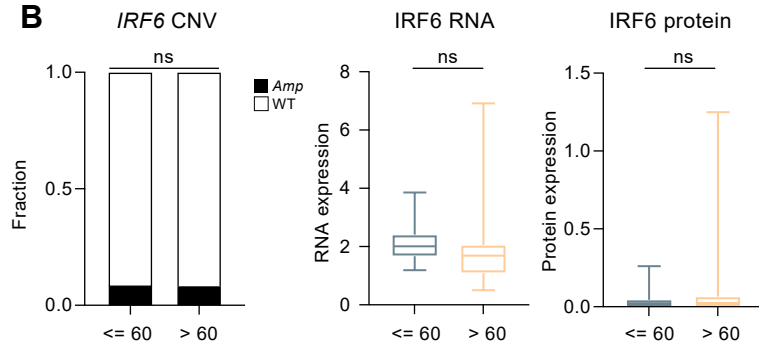

**C**

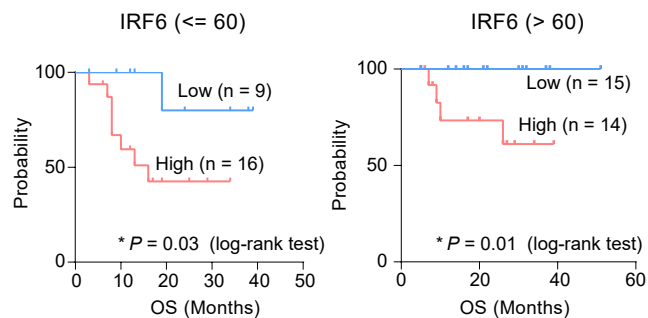

**D**

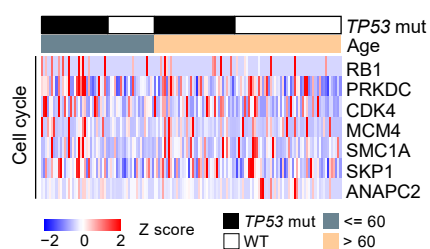

**E**

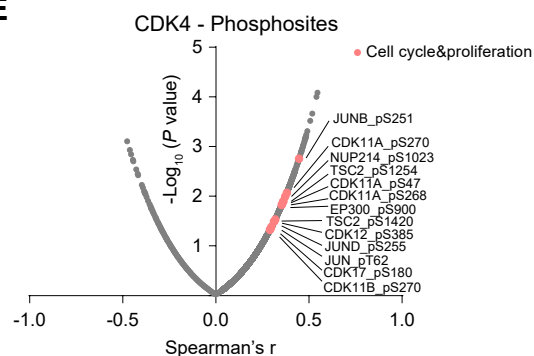

Supplement: Supplementary file 3 — Additional file 3: Fig. S3. The impacts of somatic copy number alterations in PDAC cohort, related to Fig. 2. A Bar plots showing pathway enrichment of cis/trans-effect between CNA and protein (left) or phosphoprotein (right). B Distribution of IRF6 status between the younger patients’ group (≤ 60) and the older patients’ group (> 60) (left). The boxplot reveals the comparison of the mRNA (middle) and protein (right) abundance of IRF6 between the younger patients (≤ 60) and the older patients (> 60) (Wilcoxon test). C Kaplan-Meier curves for overall survival based on IRF6 abundance in the younger patients (left) or the older patients (right) (log-rank test). D The heatmap indicating the protein abundance of genes participated in cell cycle in the four groups (TP53 mut, younger patients; WT, younger patients; TP53 mut, older patients; WT, older patients). Each column represents a sample. E Spearman-rank correlation of the abundance of CDK4 and phosphosites. Cell cycle and cell proliferation associated phosphosites are labeled in pink. **** p < 1.0E-4, *** p < 1.0E-3, ** p < 1.0E-2, * p < 0.05, ns > 0.05. [file 13045_2022_1384_MOESM3_ESM.pdf]

**A**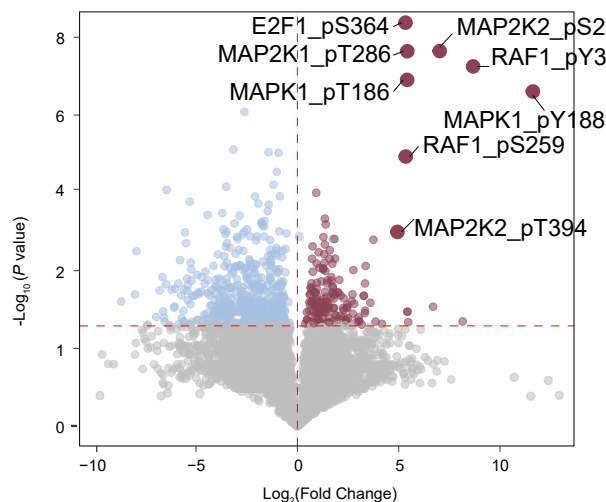**B**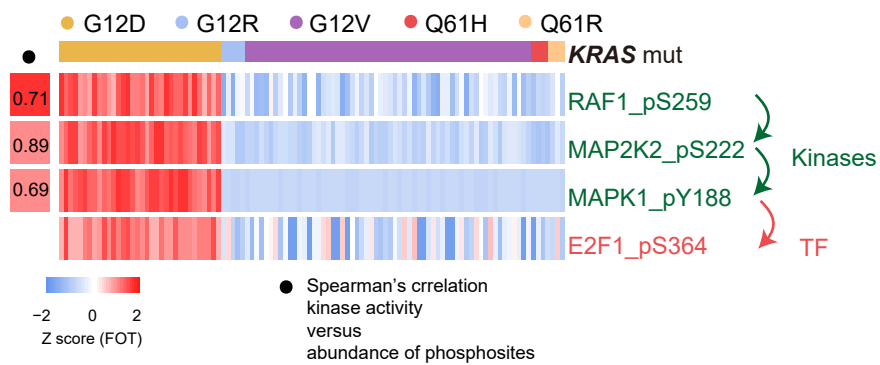**C**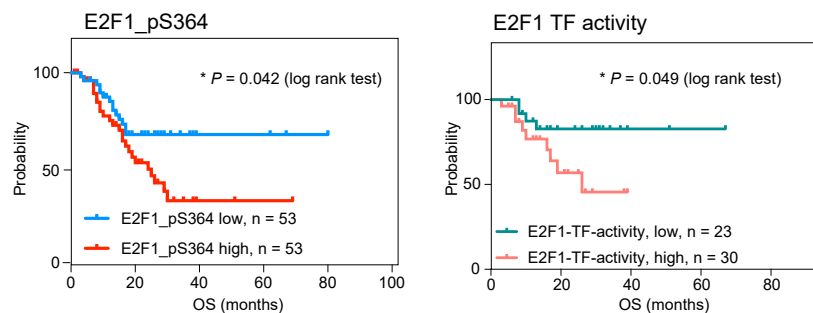**D**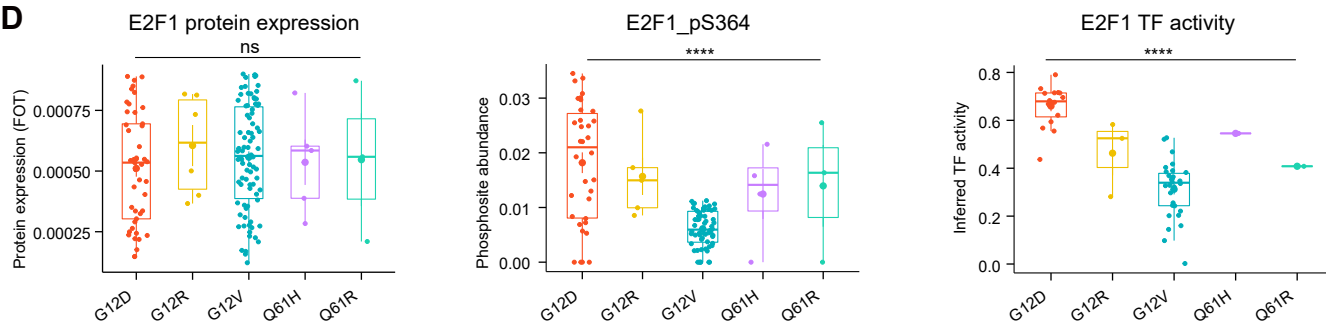**E**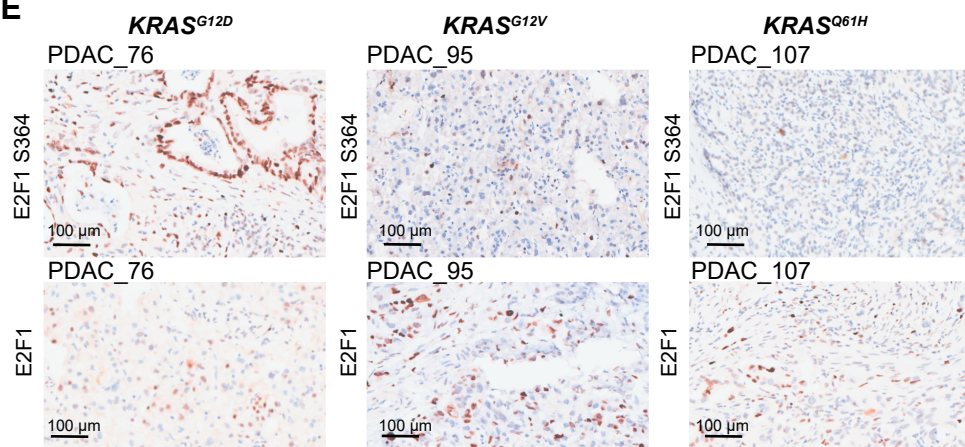**F**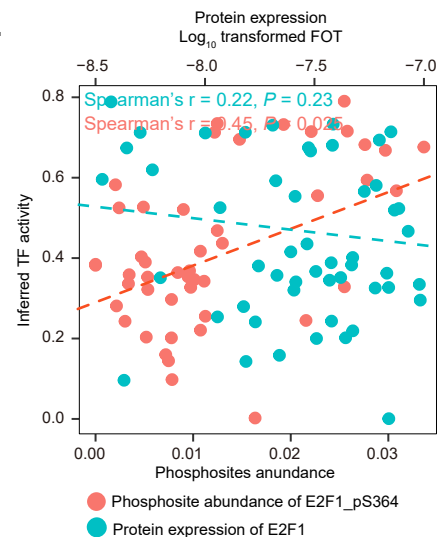**G**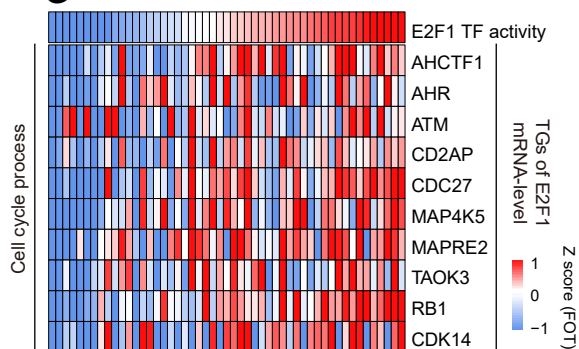**H**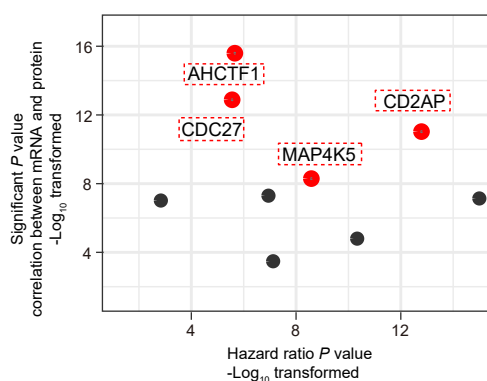**I**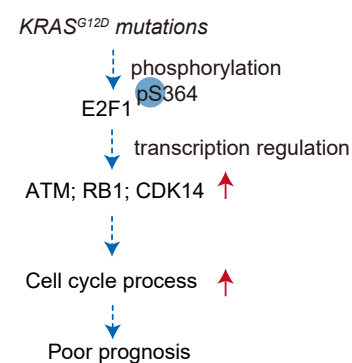

Supplement: Supplementary file 4 — Additional file 4: Fig. S4. Characteristics of PDAC patients with KRASG12D mutations. A The volcano plot showing the phosphosites that significantly altered between tumors with KRASG12D and with other types of KRAS mutations. B The heatmap indicating the phosphorylation abundance of RAF1, MAP2K2, MAPK1 across tumors with different KRAS mutations. C Kaplan-Meier curves for overall survival based on the phosphorylation of E2F1 at S364 (left) and based on the TF activity of E2F1 (right). D The box plots indicating the inferred TF activity of E2F1 (right), phosphorylation of E2F1 at S364 (middle) and the protein expression of E2F1 (left) across the tumors with diverse KRAS mutations. E IHC staining of E2F1_pS364 and E2F1 in tumors with different KRAS mutations. F The scatter plots indicating correlation between protein expression of E2F1 (green) and phosphorylation of E2F1 at S364 (red) with the TF activity of E2F1. G The heatmap revealing the expression patterns of E2F1’s TGs across the PDAC patients. Color of each cell shows the z scored FPKM of the mRNAs. H The scatter plot showing significance of correlation between proteins with their cognate mRNAs (y axis), versus the significance of protein’s Hazard ratio (x axis). Proteins negatively associated with patients’ overall survival are color coded in red. I The systematic diagram summarizing that the phosphorylation of E2F1 at S364 enhanced the cell proliferation process and led to poor prognosis in tumors with KRASG12D mutations. **** p < 1.0E-4, *** p < 1.0E-3, ** p < 1.0E-2, * p < 0.05, ns > 0.05. [file 13045_2022_1384_MOESM4_ESM.pdf]

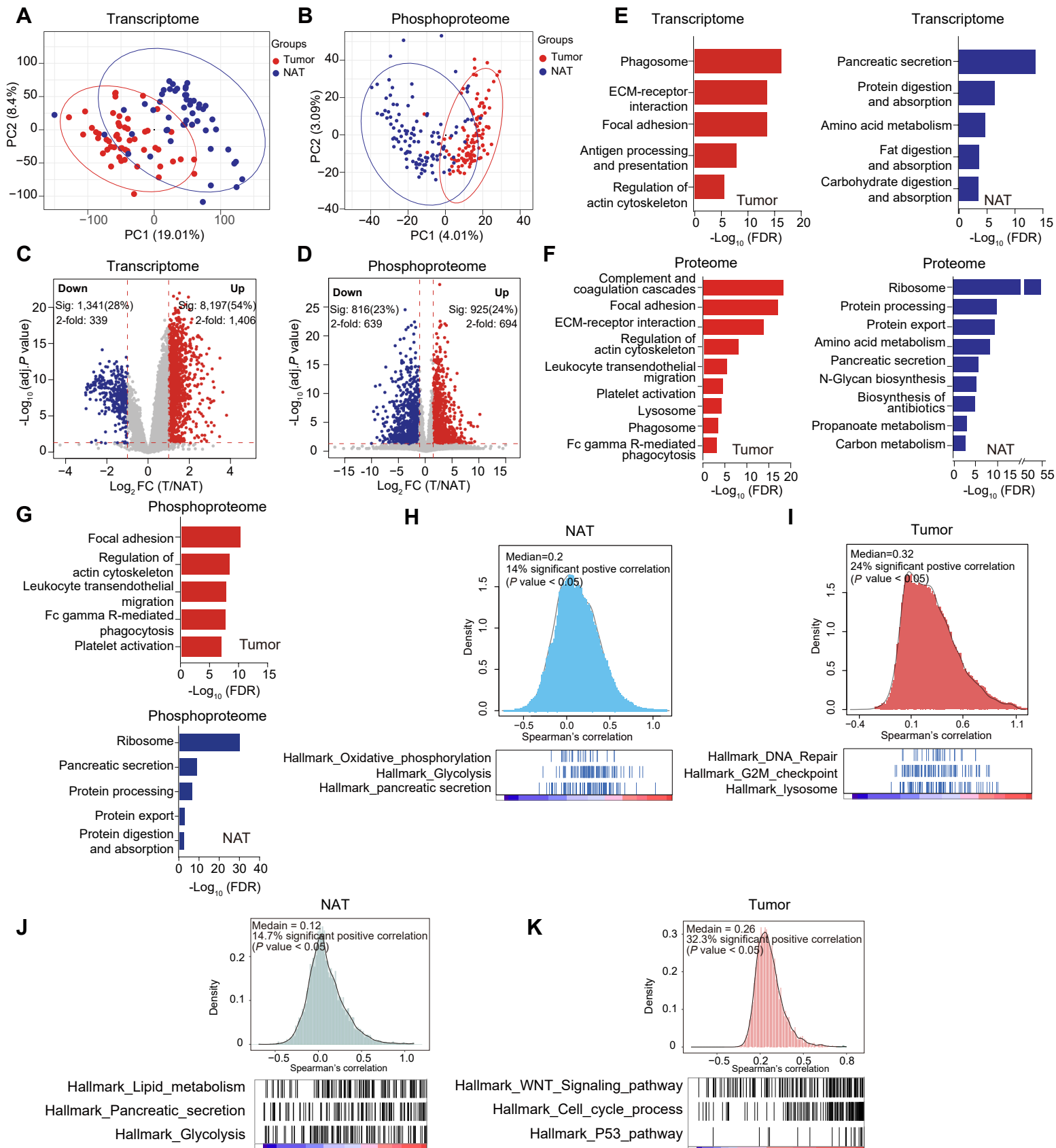

Supplement: Supplementary file 5 — Additional file 5: Fig. S5. Integrated multi-omics features in tumor tissues compared with NATs of the PDAC, related to Fig. 3. A Principal component analysis (PCA) of RNA-Seq (19,992 genes) in 54 tumors and 51 NATs. Red, tumors; blue, NATs. B PCA of 7,399 phosphoproteins in 113 paired samples. Red, tumors; blue, NATs. C A volcano plot showing the results of a two-tailed Student’s t test comparing tumors and NATs at transcriptome level. D A volcano plot showing the results of a two-tailed Student’s t test comparing tumors and NATs at phosphoproteome level. E–G. KEGG pathway analysis of differentially expressed mRNAs (E), proteins (F) and phosphoproteins (G) revealing pathways that were significantly enriched in tumors and NATs. H, I Gene-wise correlations of mRNA and protein expression in NATs (H) and tumors (I). J, K Gene-wise correlations of phosphosites and protein expression in NATs (J) and tumors (K). **** p < 1.0E-4, *** p < 1.0E-3, ** p < 1.0E-2, * p < 0.05, ns > 0.05. [file 13045_2022_1384_MOESM5_ESM.pdf]

A

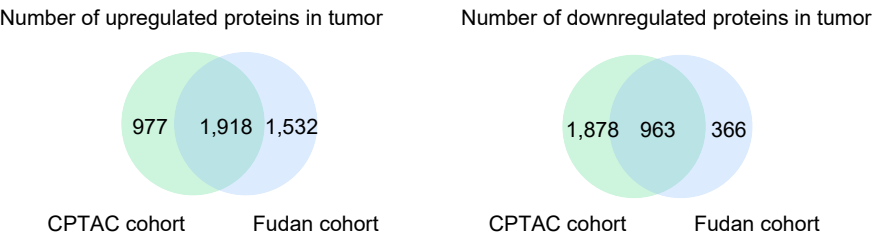

B

CPTAC cohort

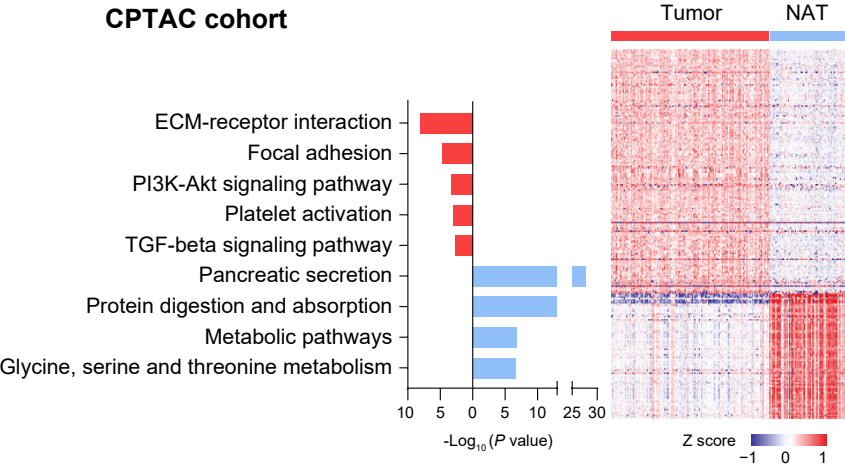

C

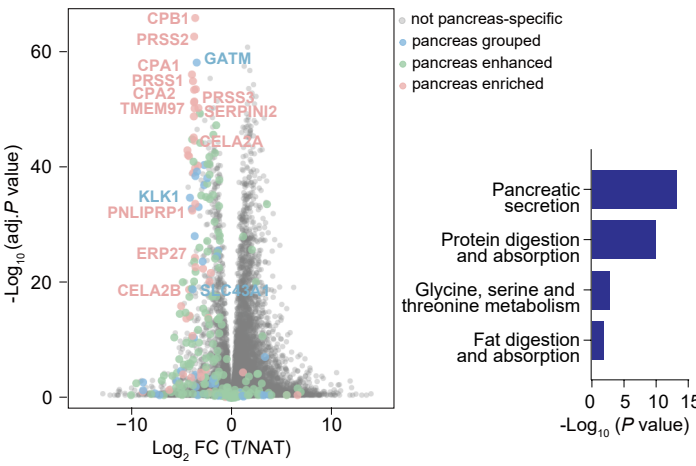

D

CPTAC cohort

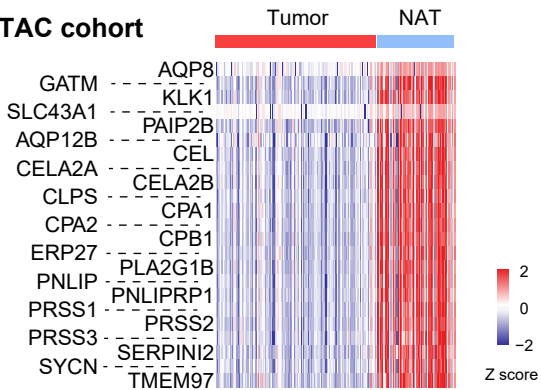

E

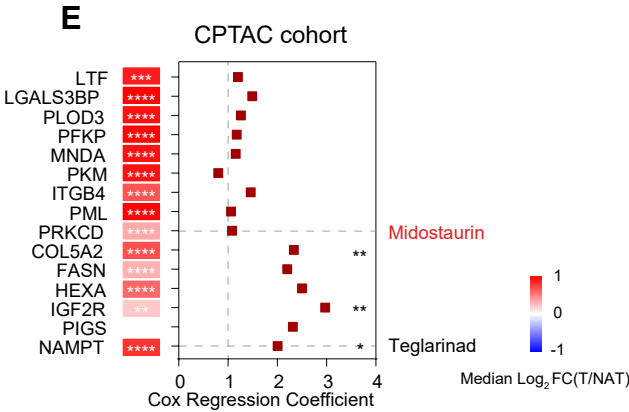

Supplement: Supplementary file 6 — Additional file 6: Fig. S6. Proteomic features in tumor tissues compared with NATs of the PDAC in CPTAC cohort, related to Fig. 3. A Venn diagram summary of the number of up-regulated (left) and down-regulated (right) proteins between Fudan cohort and CPTAC cohort. B Differentially expressed proteins in tumors and NATs in CPTAC cohort (right panel). The left panel shows KEGG pathway analysis of identified differentially expressed proteins. Red, up-regulated pathways; blue, down-regulated pathways. C Differential expression of the pancreas-specific proteins in tumors and NATs (list from The Human Protein Atlas). Red, pancreas enriched proteins; green, pancreas enhanced protein; blue, pancreas grouped protein (left panel). The right panel shows KEGG pathway analysis of identified pancreas-specific proteins. D The expression of pancreas signature proteins in tumors and NATs in CPTAC cohort. E Heatmap (left) showing the fold change of 15 potential targets between tumors and NATs in CPTAC cohort. Scatter plot (right) shows the Cox regression coefficient of these proteins. Name in red indicates FDA-approved drugs. **** p < 1.0E-4, *** p < 1.0E-3, ** p < 1.0E-2, * p < 0.05, ns > 0.05. [file 13045_2022_1384_MOESM6_ESM.pdf]

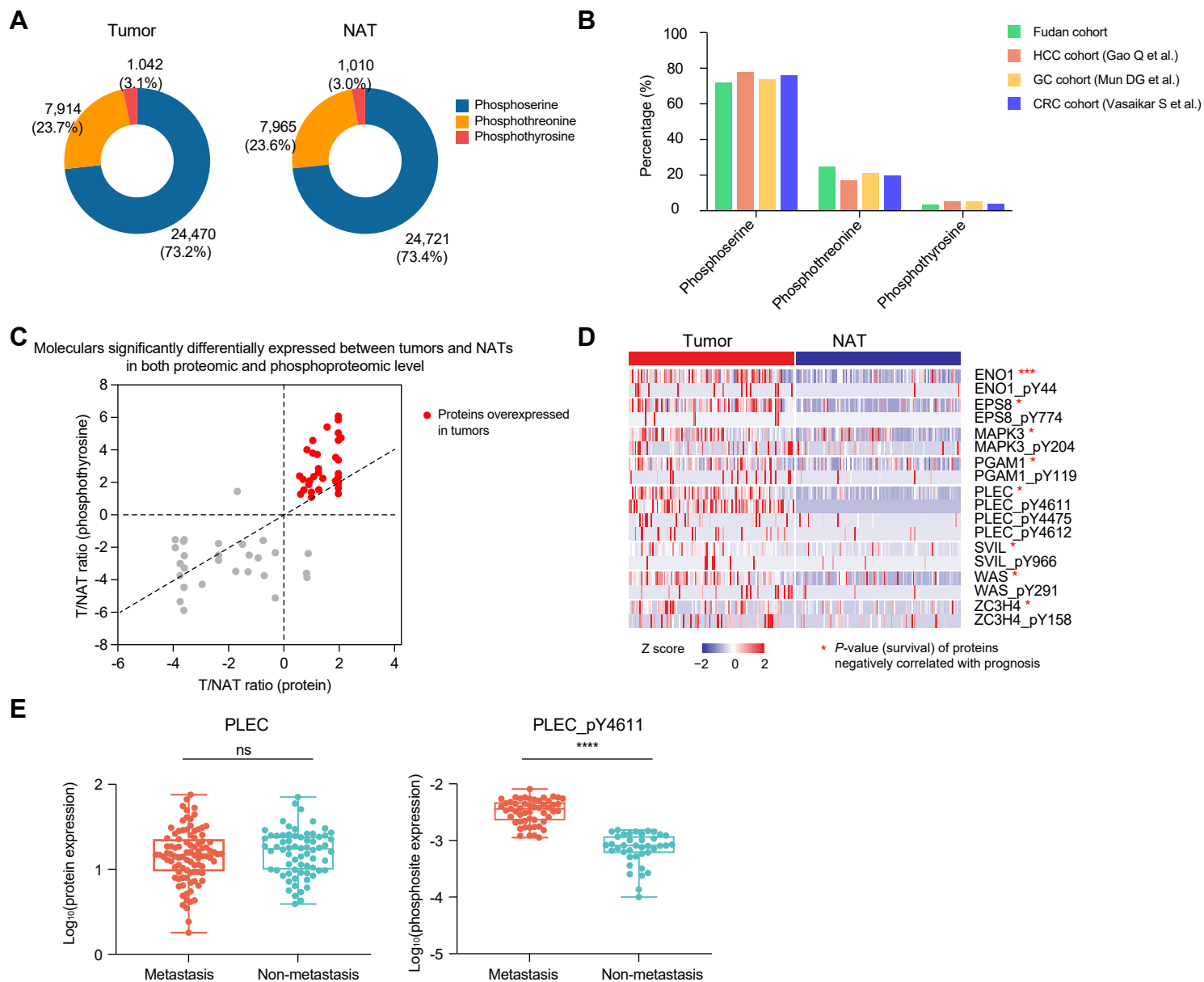

Supplement: Supplementary file 7 — Additional file 7: Fig. S7. The expression of phosphotyrosine in PDAC cohort, related to Fig. 3. A. The proportion of phosphorylation sites S, T and Y in tumors and NATs, respectively. B. The comparison of the percentage of phosphorylation sites S, T and Y in Fudan cohort and other gastrointestinal tumor cohorts. C. The scatter plot demonstrating the T/NAT ratio of phosphotyrosine (y axis) and T/NAT ratio of their corresponding proteins (x axis). D. Heatmap showing the expression of altered proteins and their corresponding phosphotyrosine. Significance (survival) of proteins which were negatively correlated with prognosis are labeled. E. Boxplot of PLEC (left) and PLEC/Y4611 (right) expression between metastasis/non-metastasis patients (Wilcoxon test). **** p < 1.0E-4, *** p < 1.0E-3, ** p < 1.0E-2, * p < 0.05, ns > 0.05. [file 13045_2022_1384_MOESM7_ESM.pdf]

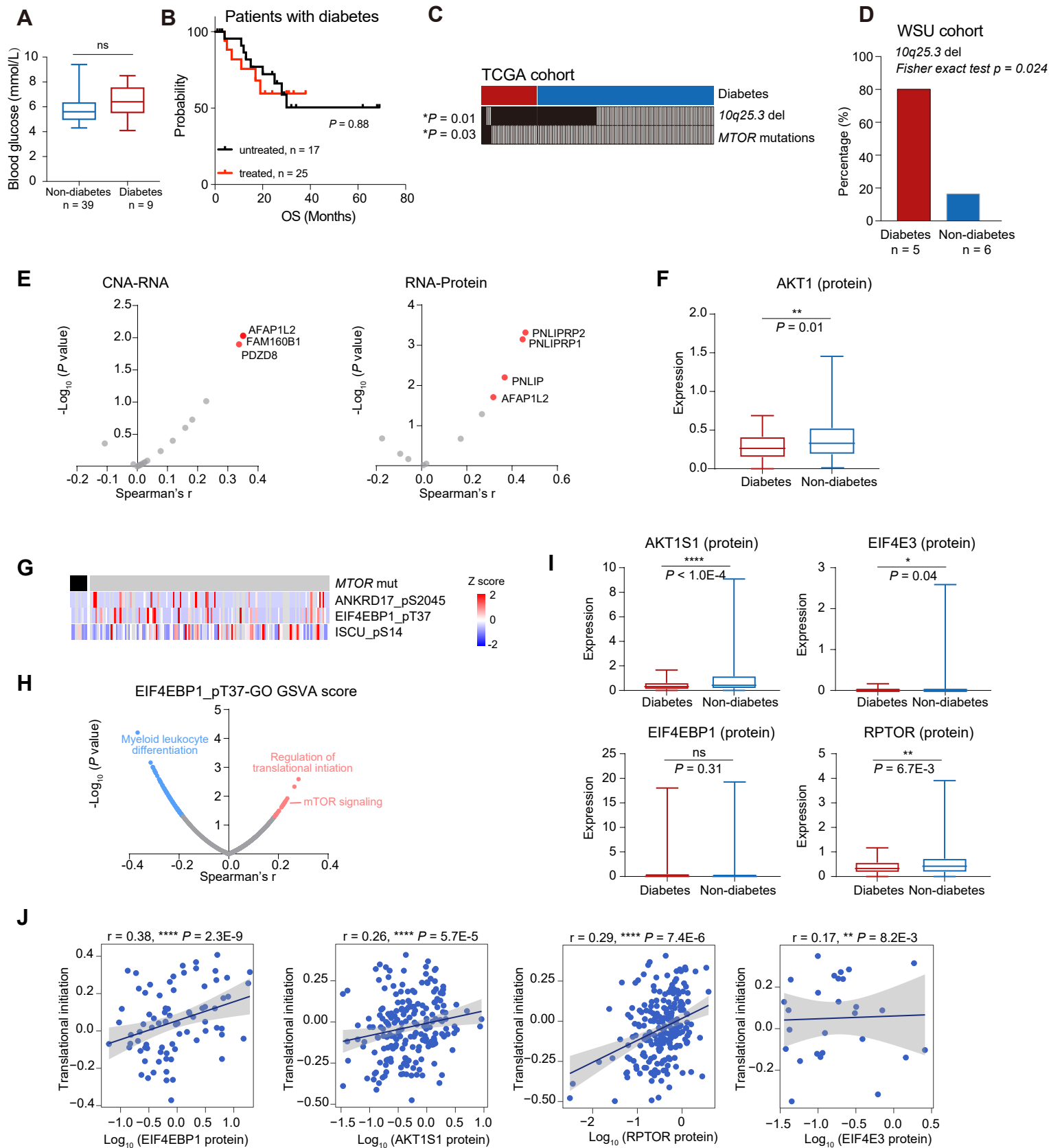

Supplement: Supplementary file 8 — Additional file 8: Fig. S8. The effects of diabetes on the proteogenomic characteristics of PDAC, related to Fig. 4. A The boxplot representing the comparison of blood glucose concentration between patients with diabetes and without diabetes. B The Kaplan-Meier curves for overall survival based on diabetic patients with or without medical treatment. C The heatmap showing the distribution of 10q25.3 deletion and MTOR mutations across different samples in TCGA cohort. D The bar plot showing the distribution of patients with 10q25.3 deletion between patients with diabetes and without diabetes in WSU cohort. E The scatter plot depicting the cis-effect of genes located in 10q25.3 between CNA and RNA (left) or RNA and protein (right). F The boxplot indicating the protein expression of AKT1 between patients with and without diabetes history (Wilcoxon test). G The expression heatmap describing the phospho-substrates of MTOR downregulated by MTOR mutations. H The scatter plot depicting the correlation between abundance of EIF4EBP1_pT37 and GSVA score (Spearman’s correlation). I The boxplots indicating the protein expression of AKT1S1, EIF4E3, EIF4EBP1, and RPTOR between patients with and without diabetes history (Wilcoxon test). J Spearman-rank correlation of the protein expression of AKT1S1, EIF4E3, EIF4EBP1, and RPTOR versus the GSVA score of translational initiation. **** p < 1.0E-4, *** p < 1.0E-3, ** p < 1.0E-2, * p < 0.05, ns > 0.05. [file 13045_2022_1384_MOESM8_ESM.pdf]

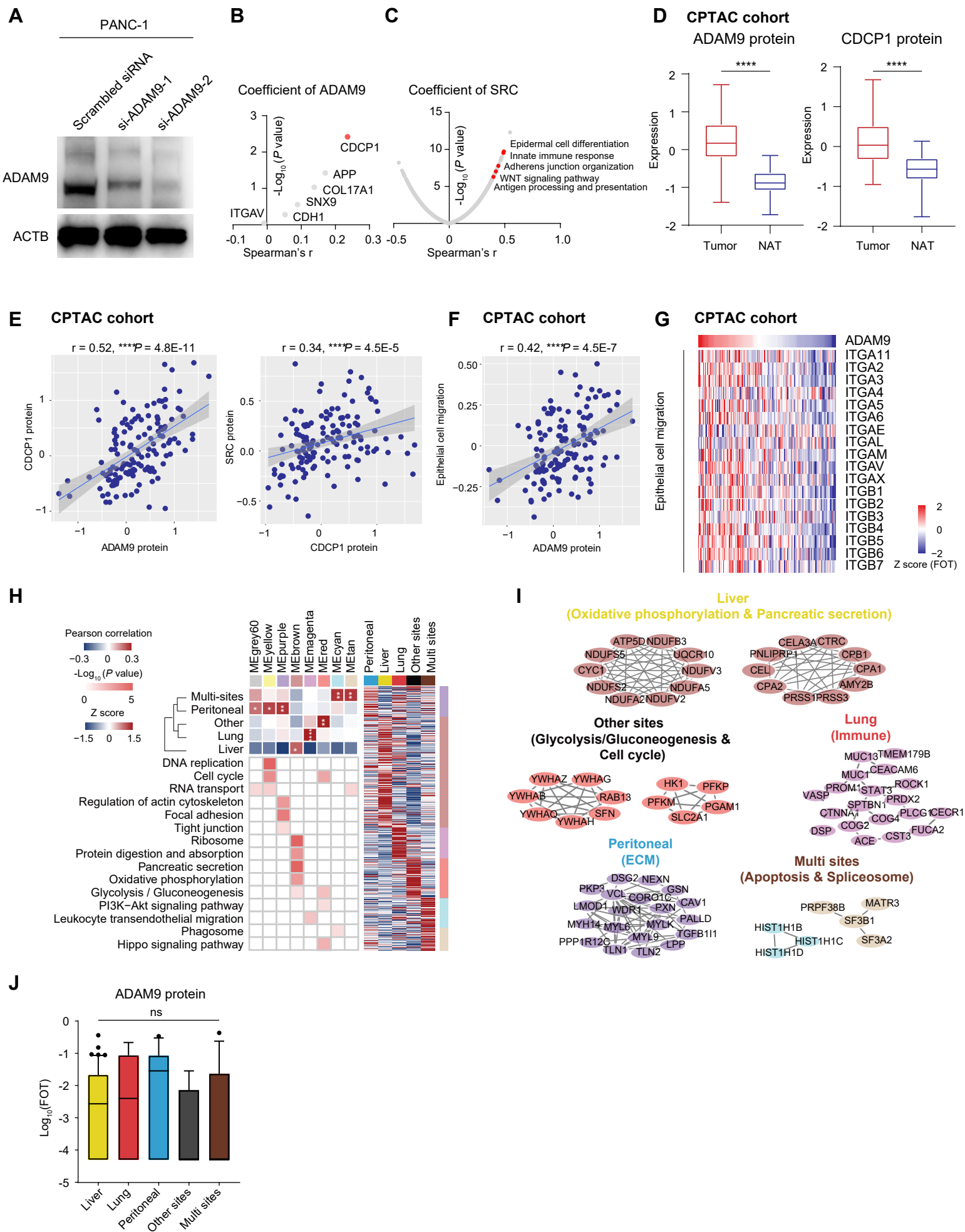

Supplement: Supplementary file 9 — Additional file 9: Fig. S9. 8p11.22 amplification is associated with PDAC metastasis, related to Fig. 5. A Western blot assays were conducted for detecting ADAM9 protein in PANC-1 cells after si-ADAM9-1 and si-ADMA9-2 knocked down. B The scatter plot depicting the correlation between ADAM9 expression and expression of ADAM9-interacting proteins (Spearman’s correlation). C The scatter plot depicting the correlation between the abundance of SRC and pathways (GSVA score). D The boxplots indicating the protein expression of ADAM9 (left) and CDCP1 (right) between tumor tissues and NATs in CPTAC cohort (Student’s t test). E Spearman-rank correlation of ADAM9 and CDCP1 protein expression (left). The scatter plot on the right depicting the correlation of CDCP1 and SRC protein expression in CPTAC cohort (Spearman’s correlation). F Spearman-rank correlation of the abundance of ADAM9 and epithelial cell migration (GSVA score) in CPTAC cohort (Spearman’s correlation). G Heatmap of the relative abundance of epithelial-cell-migration-related proteins that are significantly associated with ADAM9 expression in CPTAC cohort. H WGCNA of 93 PDAC samples depicting module eigengenes (MEs) highly correlated with primary tumor in the patients with five metastasis sites (upper heatmap of the left panel). Enrichment analysis for the different MEs is presented in the lower heatmap of the left panel (P value < 0.05). The heatmap (right) shows the differential proteins involved the overrepresented pathways of different modules corresponding to the five metastasis sites. I Protein-regulatory network showing proteins participated in metastasis-sites-associated pathways. J Boxplot of ADAM9 protein expression among five metastasis sites (Kruskal-Wallis test). **** p < 1.0E-4, *** p < 1.0E-3, ** p < 1.0E-2, * p < 0.05, ns > 0.05. [file 13045_2022_1384_MOESM9_ESM.pdf]

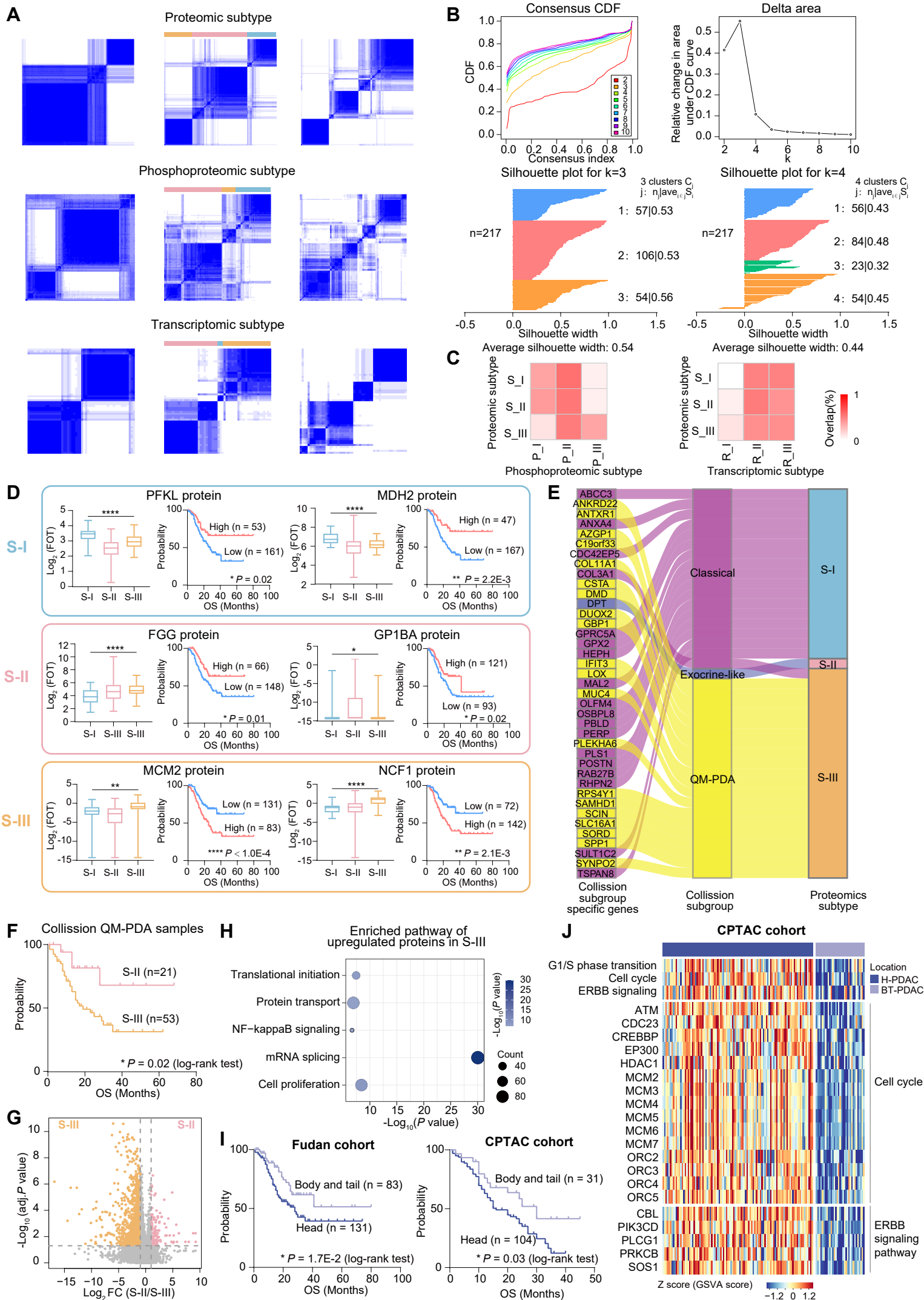

Supplement: Supplementary file 11 — Additional file 11: Fig. S11. Multi-omics subtypes of PDAC patients, related to Fig. 6. A Consensus matrices of identified clusters (k = 2 to 4) of transcriptomic, proteomic, and phosphoproteomic subtypes. B The consensus CDF and delta area (change in CDF area) plots, as well as the silhouette plots, are shown. C Heatmaps showing the comparison between transcriptomic and phosphoproteomic subtypes (columns) with proteomic subtypes (rows). Each row sums to one, with different entries showing the proportion of tumors allocated to proteomic subtypes. D The boxplots of overrepresented proteins (One-way ANOVA) and the association of the proteins with prognosis (log-rank test) among three proteomic subtypes. E The Sankey plot revealing the association between our proteomic subtypes and Collisson subgroups. F Kaplan-Meier plot comparing the survival outcomes between Collisson QM-PDA samples assigned into two proteomic clusters (S-II and S-III). G Differential expression protein between S-II and S-III included in Collisson QM-PDA samples. H Bubble diagram revealing the enriched pathway of upregulated proteins in S-III included in Collisson QM-PDA samples. I Kaplan-Meier curves for overall survival of patients with tumor located on head (H-PDAC) and with tumor located on body-tail (BT-PDAC) in Fudan cohort (left) and CPTAC cohort (right) (log-rank test). J The heatmap indicating the GSVA scores of the pathway at proteomic level (upper) and proteins enriched in cell cycle and ERBB signaling pathway (lower) between H-PDAC and BT-PDAC in CPTAC cohort. **** p < 1.0E-4, *** p < 1.0E-3, ** p < 1.0E-2, * p < 0.05, ns > 0.05. [file 13045_2022_1384_MOESM11_ESM.pdf]

**A**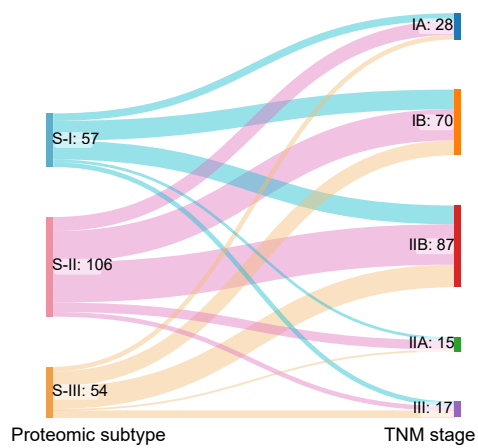**B**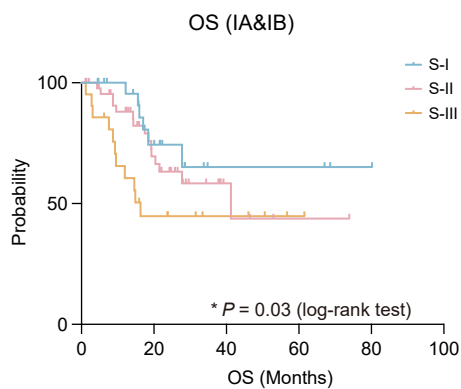**C**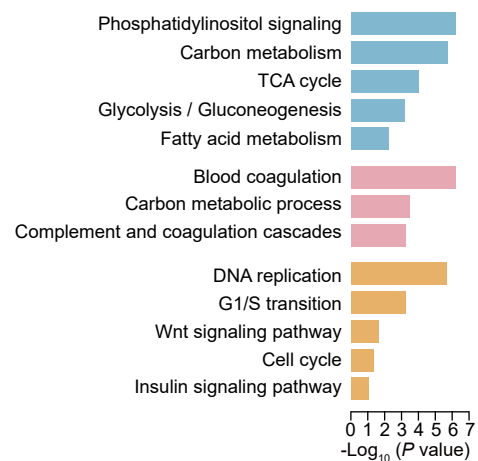**D**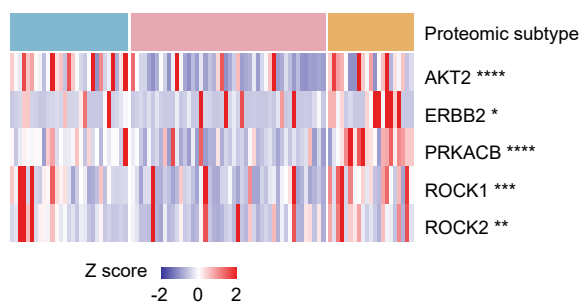

Supplement: Supplementary file 13 — Additional file 13: Fig. S13. Proteomic subtypes divided early-stage PDAC patients into subtypes with different prognosis, related to Fig. 6. A Summary of the relationship between proteomic subtypes and TNM stage subtypes. B Kaplan-Meier curves for overall survival of early-stage (IA and IB) PDAC patients based on 3 proteomic subtypes (log-rank test). C Bar plot indicating the enriched pathways in early-stage patients who were divided into three proteomic subtypes. D Heatmap of differentially expressed kinase signatures among the three proteomic subtypes in patients with early-stage PDAC. **** p < 1.0E-4, *** p < 1.0E-3, ** p < 1.0E-2, * p < 0.05, ns > 0.05. [file 13045_2022_1384_MOESM13_ESM.pdf]

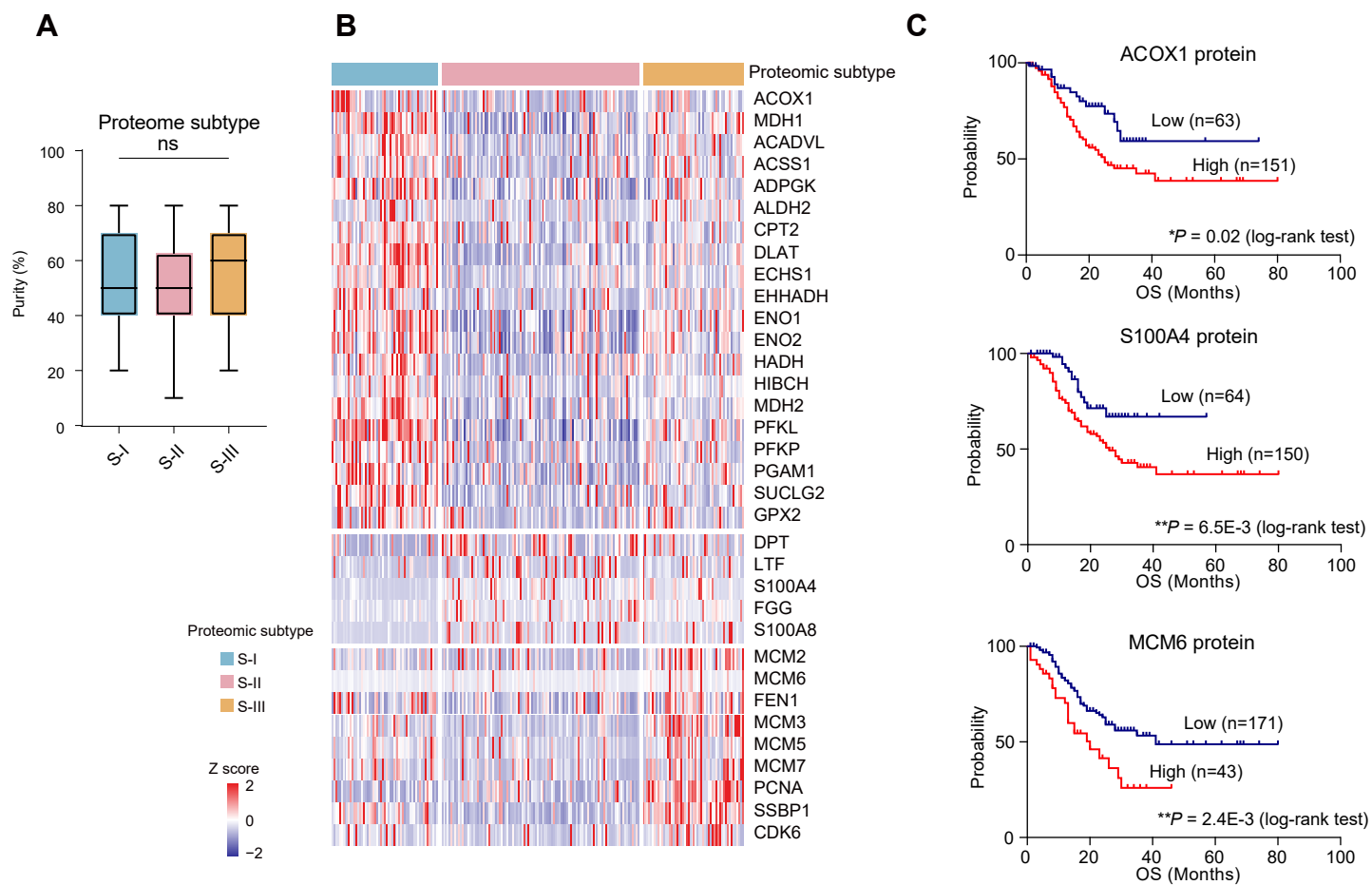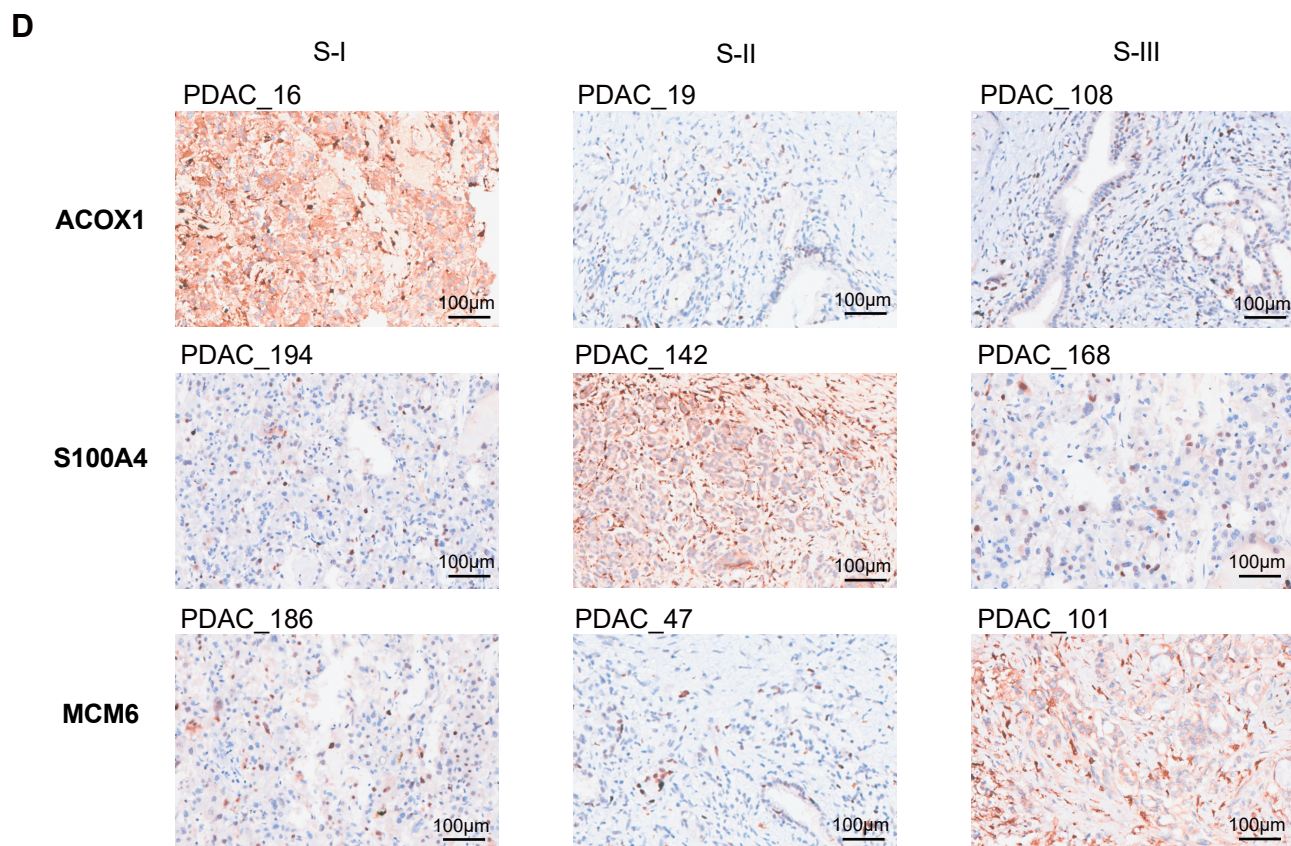

Supplement: Supplementary file 15 — Additional file 15: Fig. S15. Protein markers for three PDAC proteomic subtypes, related to Fig. 6. A The boxplot indicating the comparison of tumor purity across the proteomic subtypes. B Heatmap indicating the expression of protein biomarkers in the three proteomic subtypes. C Kaplan-Meier curves for overall survival based on proteomic abundance of ACOX1, S100A4 and MCM6 (log-rank test). D IHC profiling of proteomic subtype markers in PDAC. FFPE sections were stained for ACOX1, S100A4 and MCM6 protein markers in PDAC tumor tissues. The scale bar indicates 100 μm. **** p < 1.0E-4, *** p < 1.0E-3, ** p < 1.0E-2, * p < 0.05, ns > 0.05. [file 13045_2022_1384_MOESM15_ESM.pdf]

**A**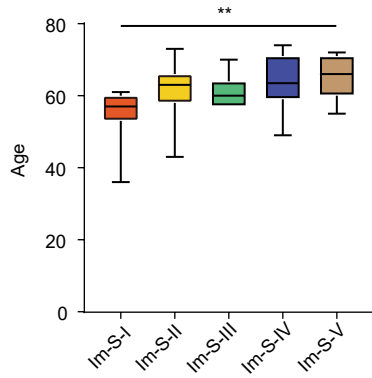**B**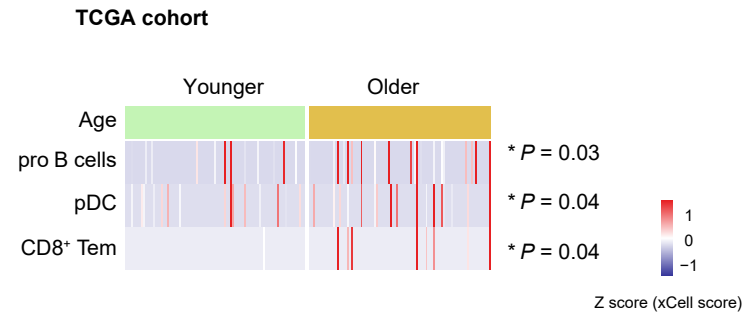**C**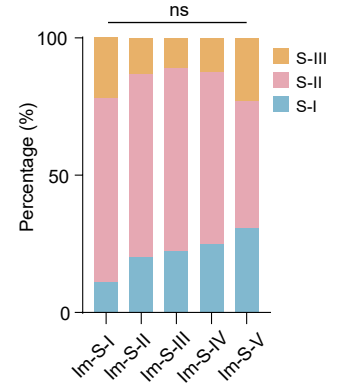**D**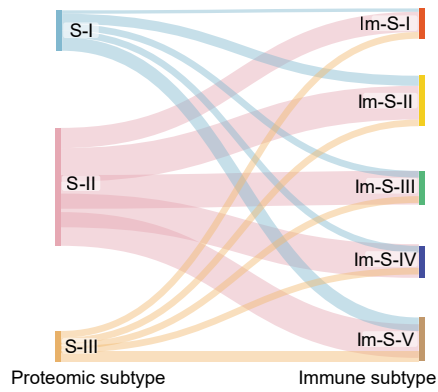**E**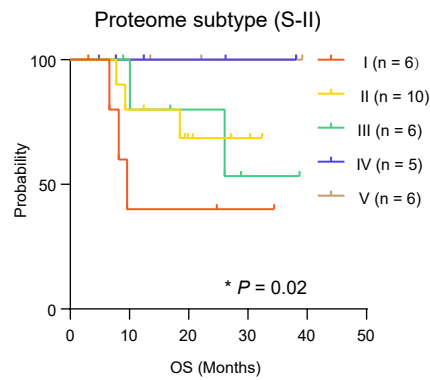**F**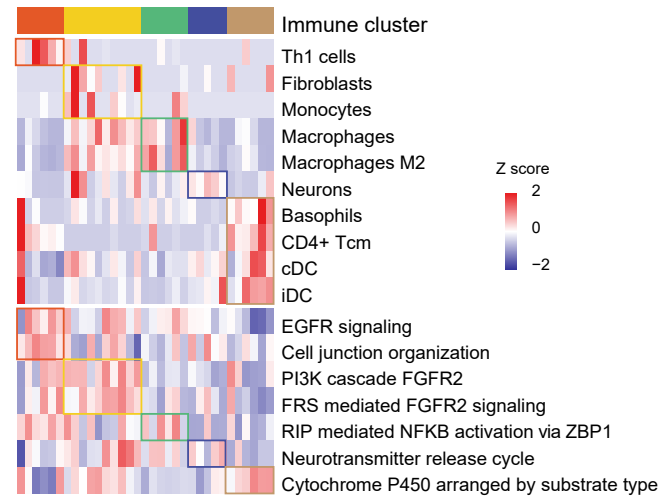**G**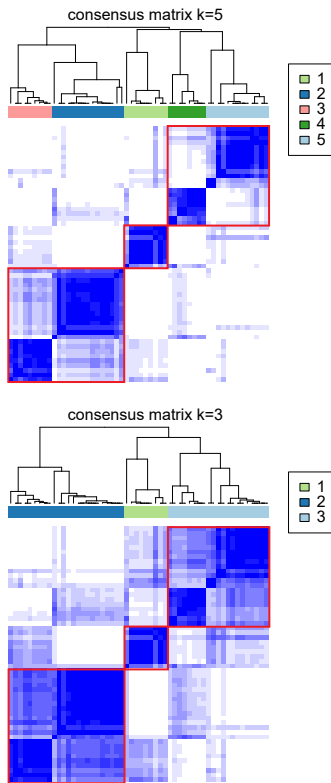**H**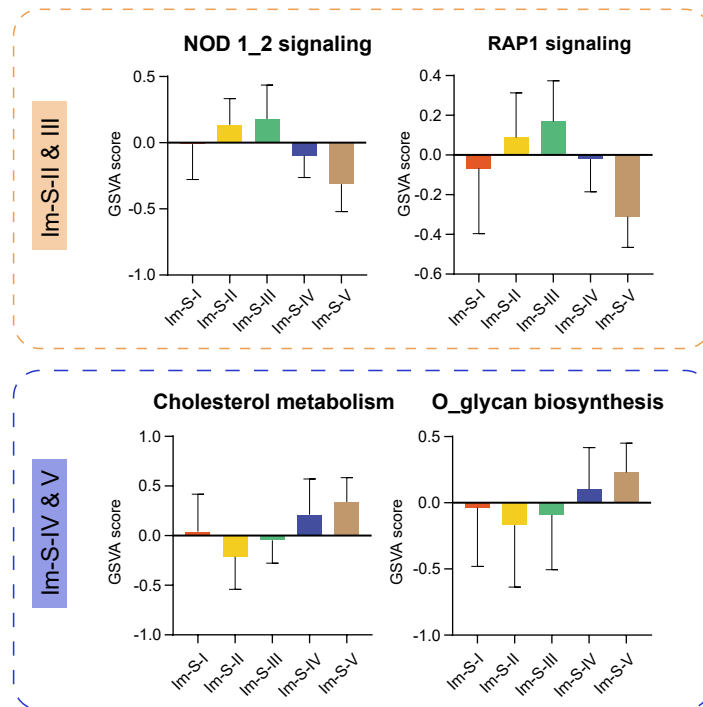

Supplement: Supplementary file 16 — Additional file 16: Fig. S16. Characterization of immune infiltration in PDAC, related to Fig. 7. A Boxplot showing the age among five immune clusters. B Heatmap of xCell score between samples in old/young group of TCGA cohort. C Distribution of the percentage of proteomics subtypes across different immune subtypes (Fisher’s exact test). D The Sankey plot revealing the association between proteomics subtypes and immune subtypes. E Kaplan-Meier curves for overall survival based on immune subtypes in S-II (log-rank test). F Heatmap illustrating cell type compositions and pathways across 5 immune clusters. G Consensus matrices of identified clusters (k = 3 and 5) of immune subtypes. H Bar plots of GSVA scores among 5 immune subtypes. **** p < 1.0E-4, *** p < 1.0E-3, ** p < 1.0E-2, * p < 0.05, ns > 0.05. [file 13045_2022_1384_MOESM16_ESM.pdf]

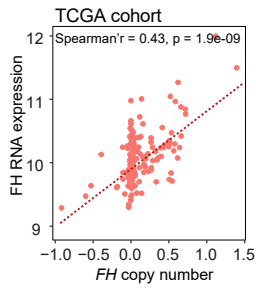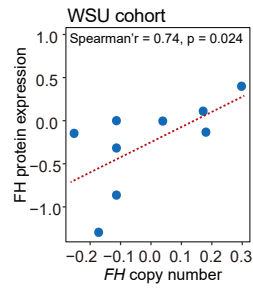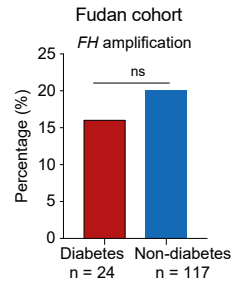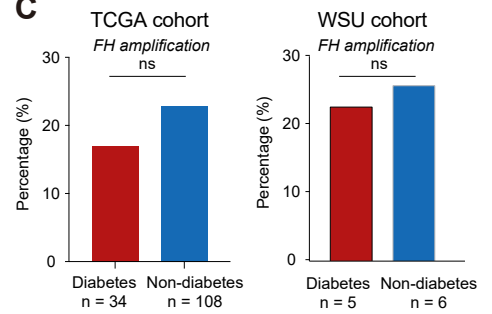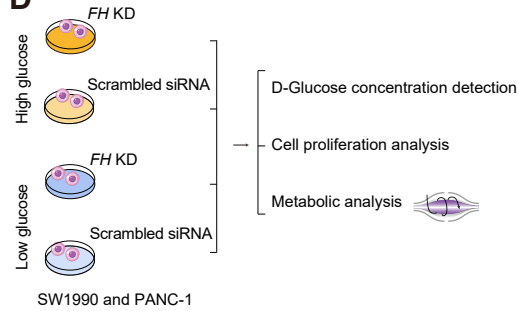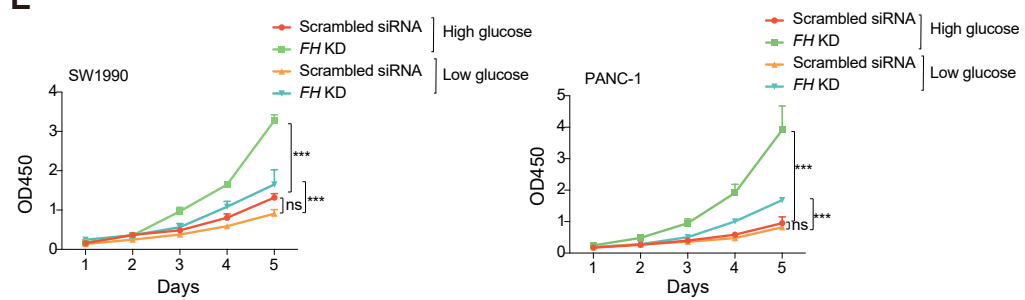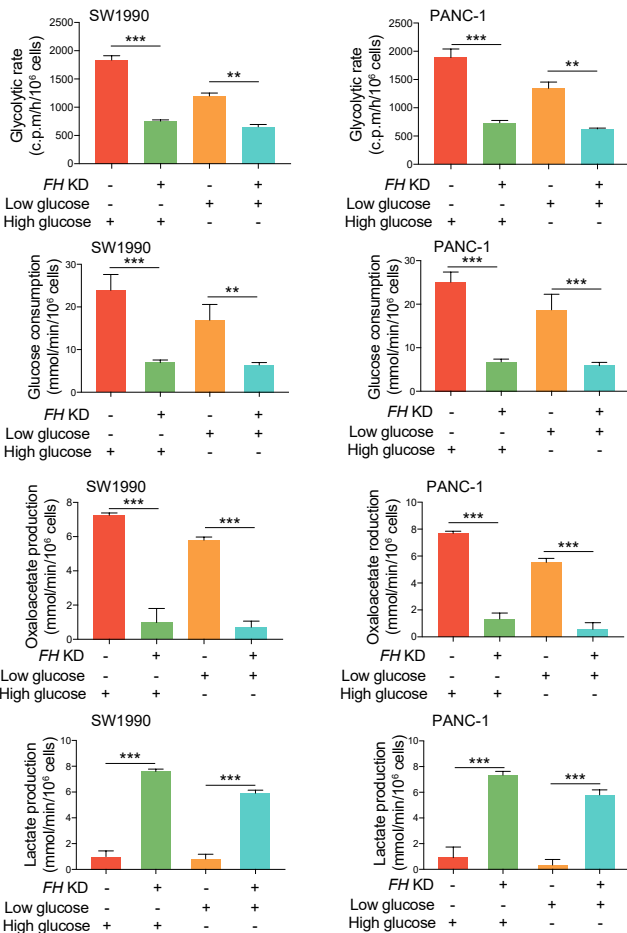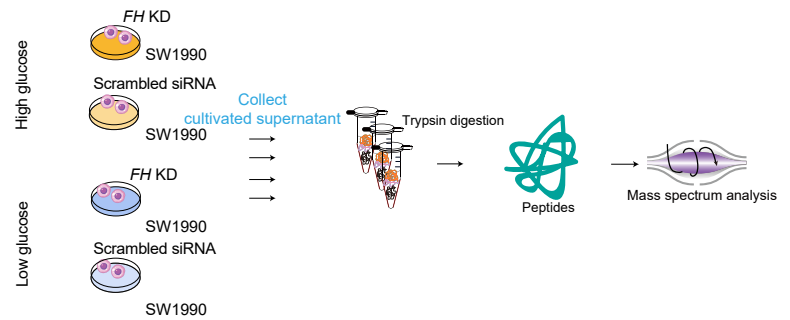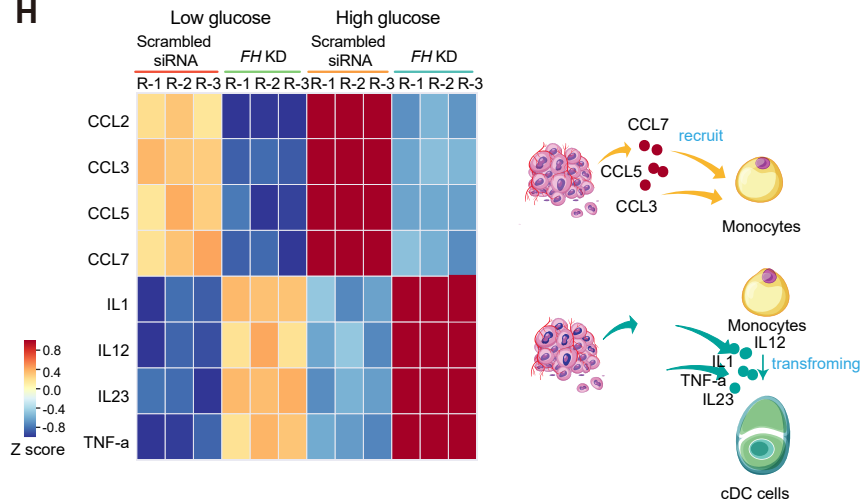

Supplement: Supplementary file 17 — Additional file 17: Fig. S17. The impact of FH amplification in PDAC, related to Fig. 7. A Scatter plot on the left presents the correlation between FH copy number and FH expression in TCGA cohort (left) or in WSU cohort (right). B, C The bar plot shows the distribution of patients with FH amplification between diabetes and non-diabetes in our cohort (B), in TCGA cohort (C, left) and in WSU cohort (C, right). D The schematic work flow of our validation experiments for the FH in prompting PDAC tumor cell proliferation through regulating glucose metabolism. E Proliferation of PDAC cells (SW1990: left; PANC1: right) associated with various treatments (n = 4 repeats per group). F The comparison of glycolytic rates, glucose consumption, oxaloacetate and lactate production associated with various treatments (n = 4 repeats per group). G The schematic work flow of our validation experiments for the impacts of FH on tumor immune microenvironments. H The heatmap showing the expression patterns of cytokines detected in supernatants of cell cultures across cells under different treatments (n = 3 repeats per group). The schematic diagrams on the right shows the impacts of cytokines such as IL12, TNF in transforming monocytes to cDCs (up); the impacts of cytokines including CCL7, CCL3 in recruiting monocytes (bottom). **** p < 1.0E-4, *** p < 1.0E-3, ** p < 1.0E-2, * p < 0.05, ns > 0.05. [file 13045_2022_1384_MOESM17_ESM.pdf]

**A**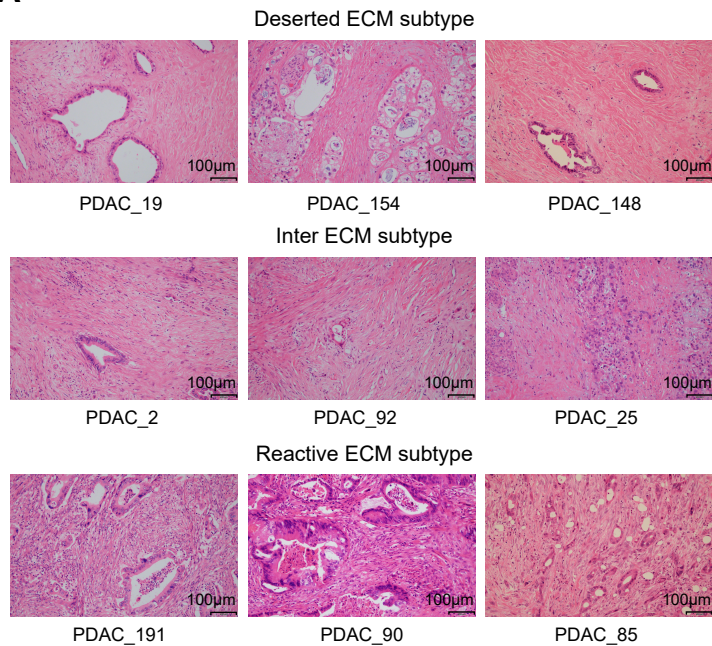**B**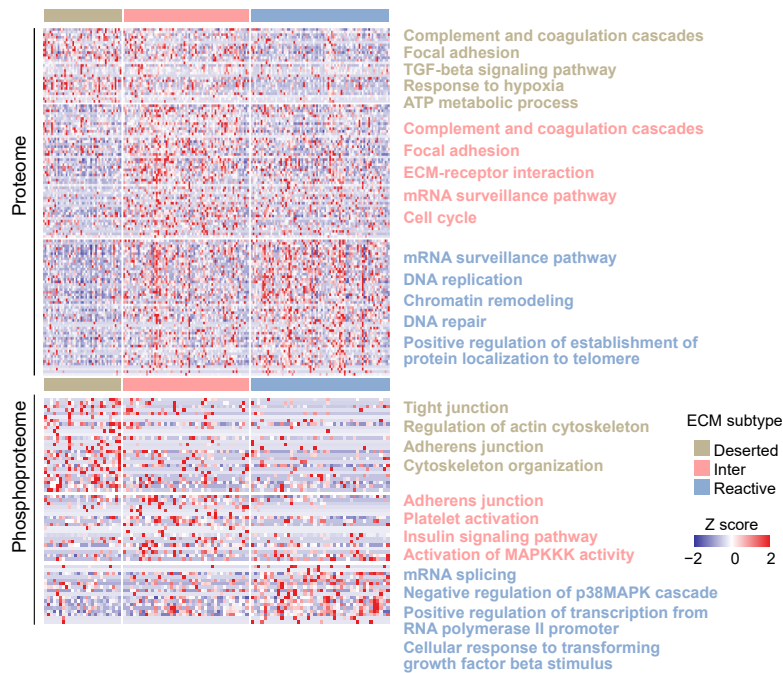**C**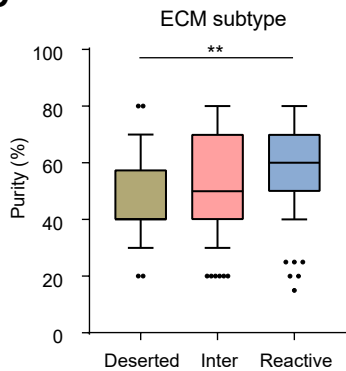**D**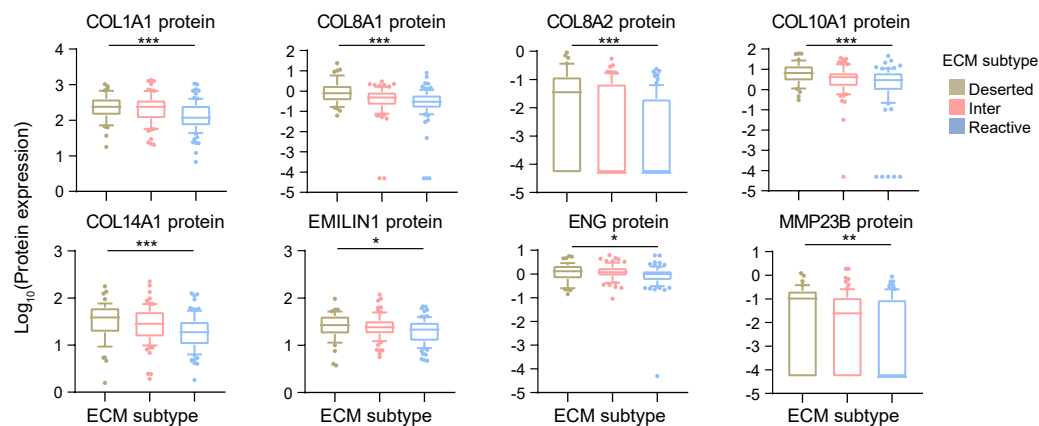**E**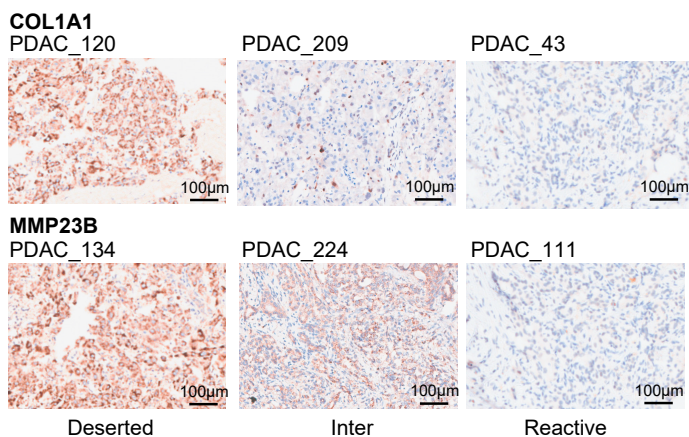**F**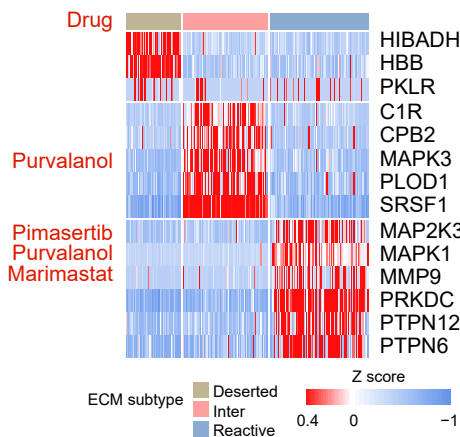**G**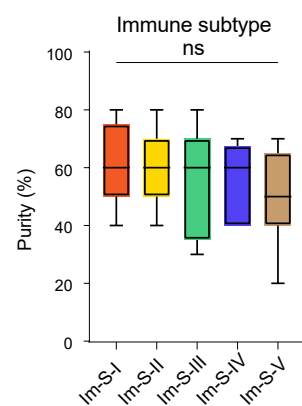**H**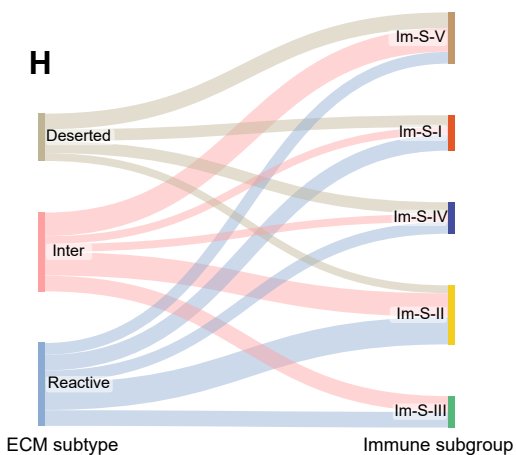**I**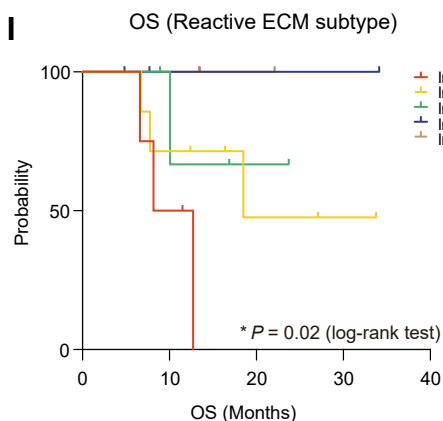**J**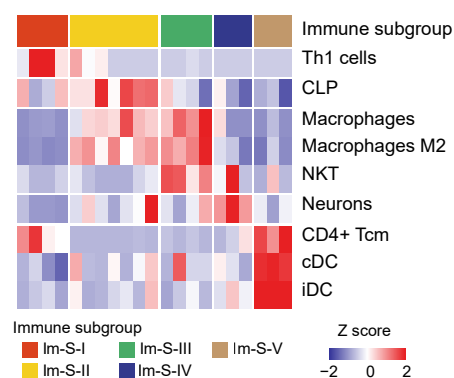

Supplement: Supplementary file 18 — Additional file 18: Fig. S18. Characterization of ECM subtypes in PDAC, related to Fig. 7. A H&E-stained slides of samples of deserted, inter, reactive ECM subtypes. B The expression heatmap describing differentially expressed proteins and phosphoproteins among three ECM subtypes. The enriched biological pathways were annotated on the right. C Boxplot illustrating the purity of sample among ECM subtypes. D Boxplots showing the expression of ECM markers among ECM subtypes. E IHC profiling of ECM markers in ECM subtypes. F Heatmap of drug targets of deserted, inter, reactive ECM subtypes. Drugs are labeled on the left. G Boxplot illustrating the purity of sample among 5 immune subtypes. H The Sankey plot revealing the association between ECM subtypes and our immune subtypes. I The association of five immune clusters with clinical outcomes in reactive ECM subtype samples. J Heatmap showing the xCell signatures among five immune clusters in reactive ECM subtype samples. **** p < 1.0E-4, *** p < 1.0E-3, ** p < 1.0E-2, * p < 0.05, ns > 0.05. [file 13045_2022_1384_MOESM18_ESM.pdf]

**A**

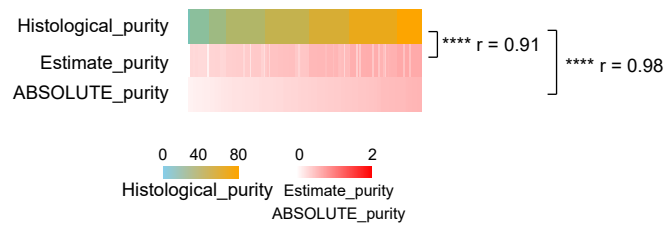

**B**

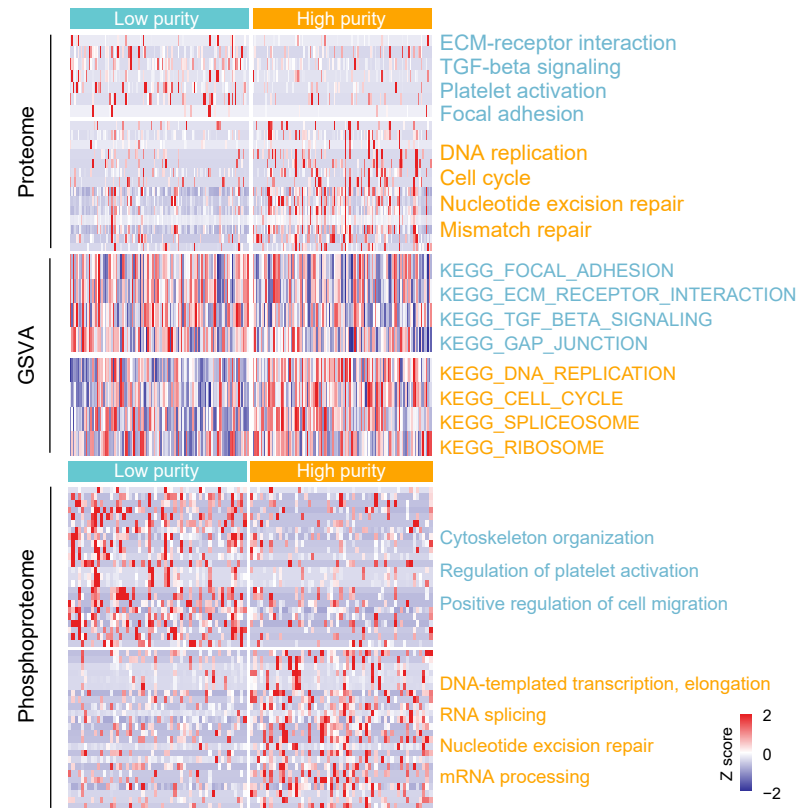

Supplement: Supplementary file 19 — Additional file 19: Fig. S19. Biological diversity between high and low purity samples. A The heatmap indicating the correlation between histological purity and purity assessed by ESTIMATE and ABSOLUTE package. B The expression heatmap describing differentially expressed proteins and phosphoproteins in high/low tumor purity. The enriched biological pathways are annotated on the right. [file 13045_2022_1384_MOESM19_ESM.pdf]

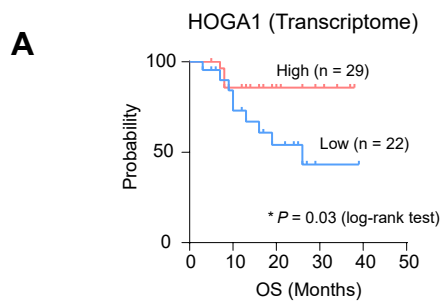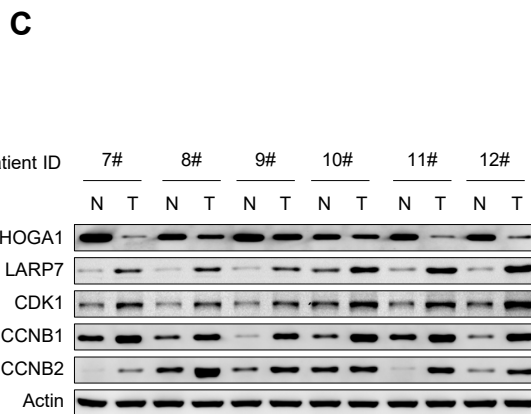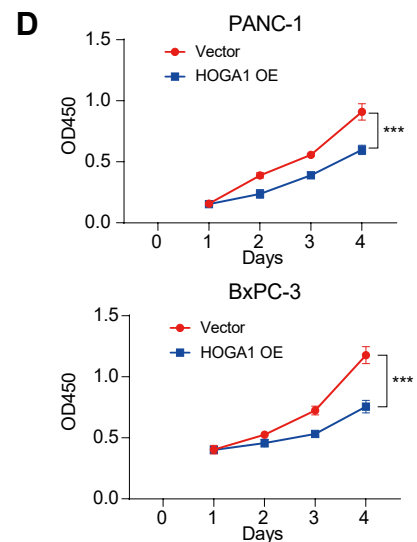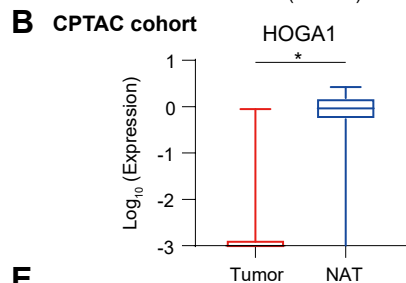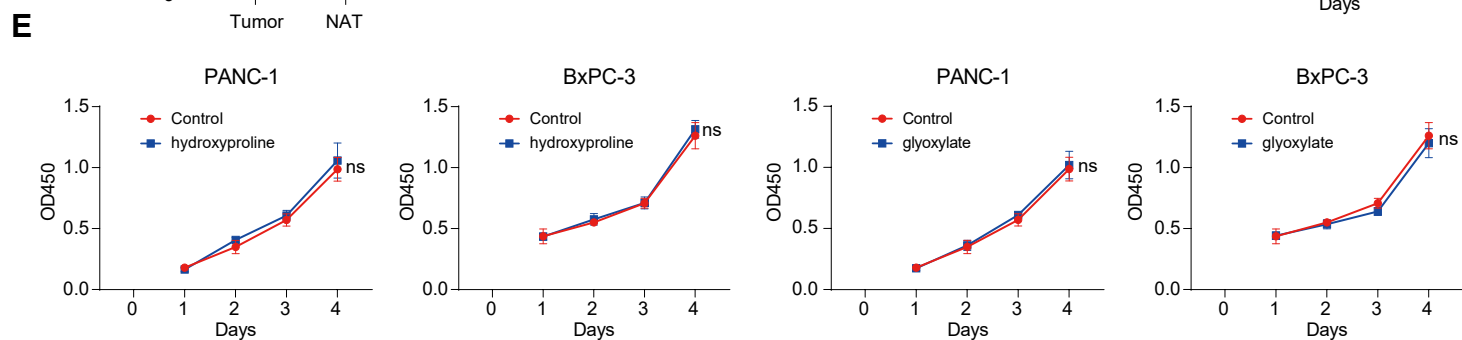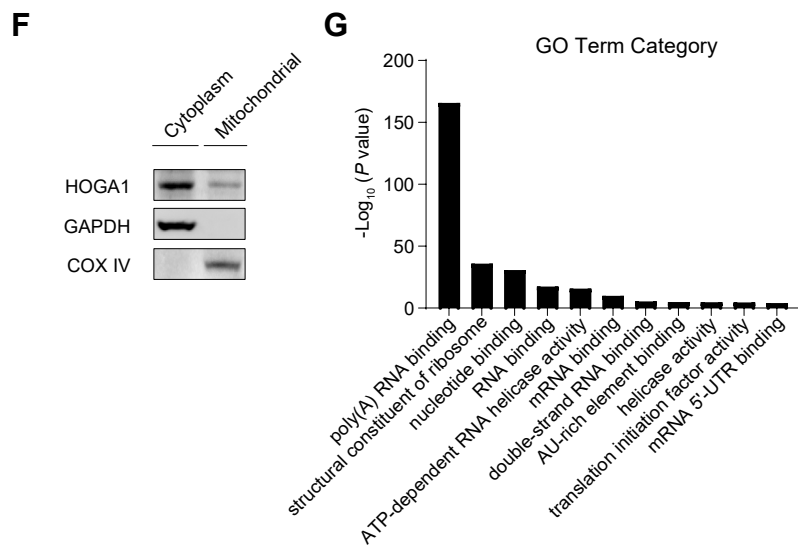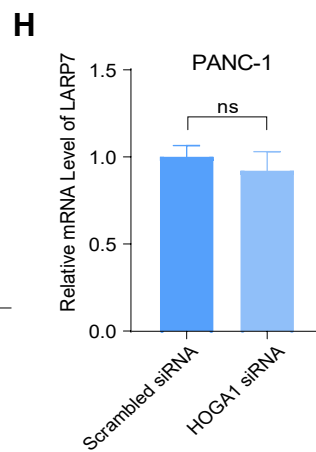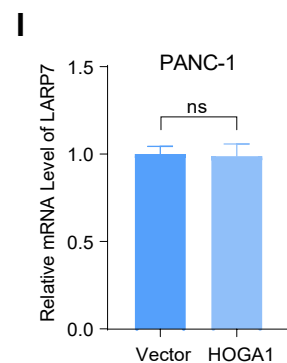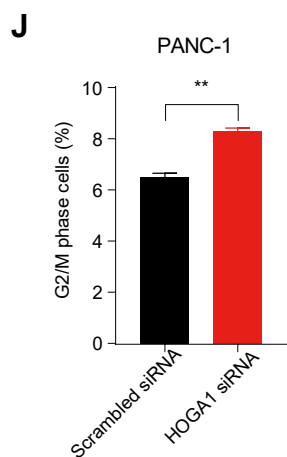

Supplement: Supplementary file 20 — Additional file 20: Fig. S20. HOGA1 regulated LARP7 expression, related to Fig. 8. A The association of the mRNA expression of HOGA1with prognosis (log-rank test). B Boxplot showing the differential expression of HOGA1 between tumors and NATs in CPTAC cohort (Student’s t test). C Expression of HOGA1, LARP7, CDK1, CCNB1, and CCNB2, in tumor tissues were detected by western blot analysis. N = non-tumor adjacent tissue, T = tumor tissue. D The impacts of HOGA1 overexpression on PANC-1 and BxPC-3 cells proliferation. E Proliferation of PANC-1 and BxPC-3 cells associated with various treatments (n = 4 repeats per group). F Cellular localization of HOGA1 in PANC-1 cells. G GO term category of HOGA1 interacting proteins. H–I LARP7 mRNA levels in PANC-1 cells with various treatments. J The percentage of G2/M phase cells in PANC-1 cells (n = 5 repeats per group). **** p < 1.0E-4, *** p < 1.0E-3, ** p < 1.0E-2, * p < 0.05, ns > 0.05. [file 13045_2022_1384_MOESM20_ESM.pdf]

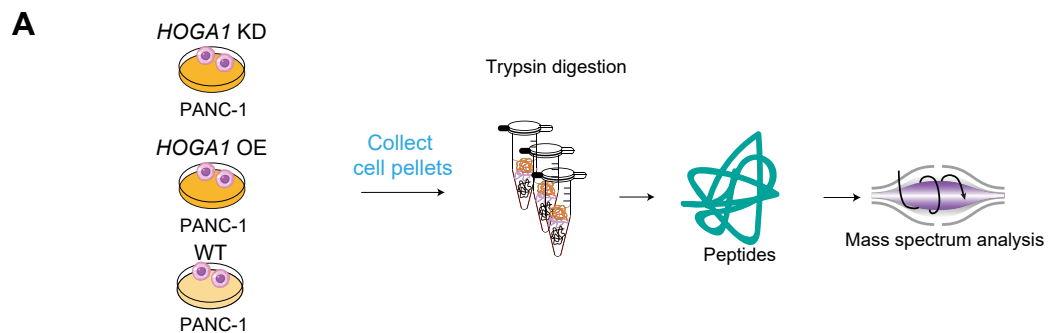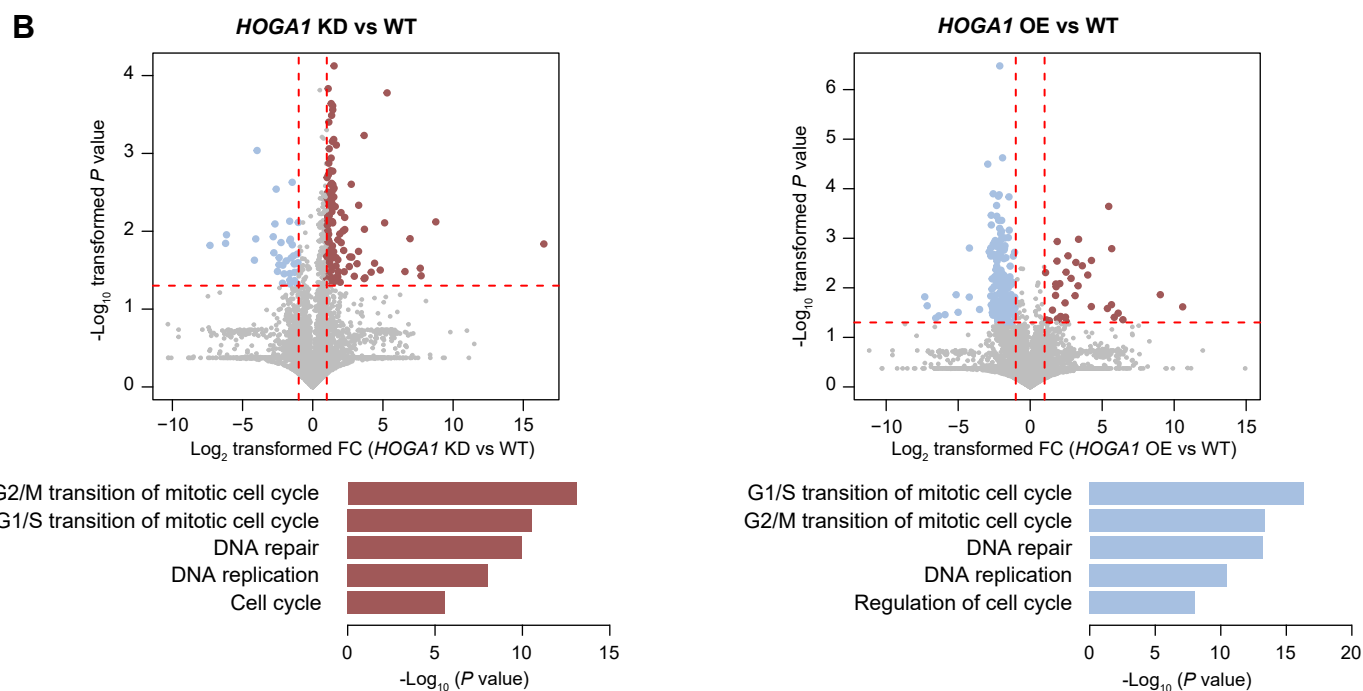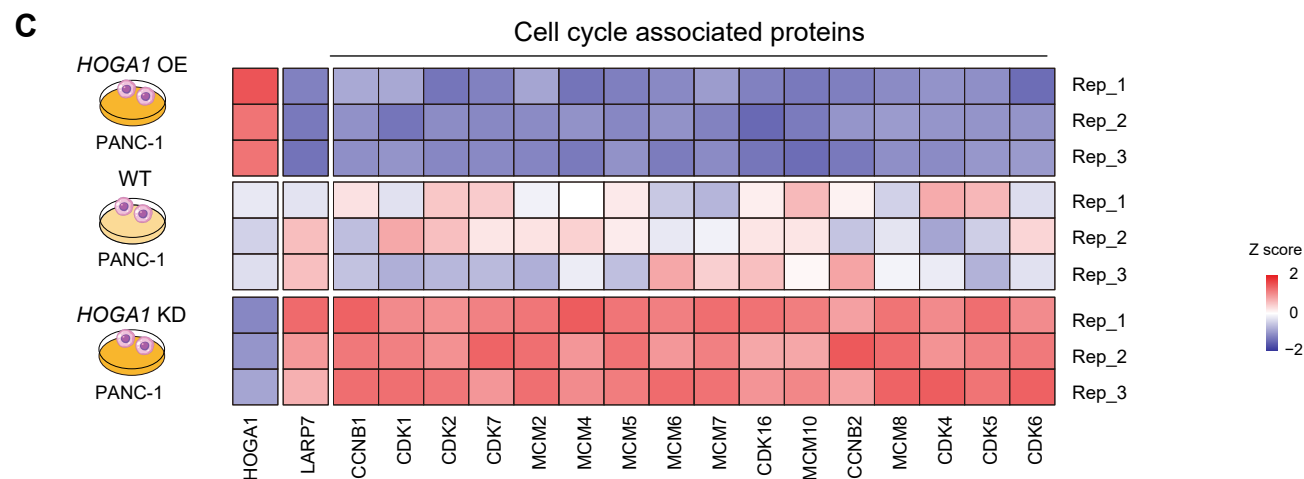

Supplement: Supplementary file 21 — Additional file 21: Fig. S21. Protein expression changes in PANC-1 cell lines after HOGA1 knocked down and over expressed, related to Fig. 8. A The schematic work flow of validation experiments for the protein expression changes after HOGA1 knocked down and over expressed. B The volcano plot showing the proteins that significantly altered between PANC-1 cells with HOGA1 KD (left)/HOGA1 OE (right) and WT. The GO processes enriched by proteins upregulated in HOGA1 KD cell lines and downregulated in HOGA1 OE cell lines compared with WT are noted on the bottom. C Expression of proteins involved in cell cycle in the HOGA1 OE group, HOGA1 KD group and WT group. (n = 3 repeats per group). [file 13045_2022_1384_MOESM21_ESM.pdf]
