# Supplementary material for: Proteogenomic insights into the biology and treatment of pancreatic ductal adenocarcinoma
Source: J Hematol Oncol. 2022 Nov 25;15:168. doi: 10.1186/s13045-022-01384-3 (PMC9701038; doi:10.1186/s13045-022-01384-3)

A

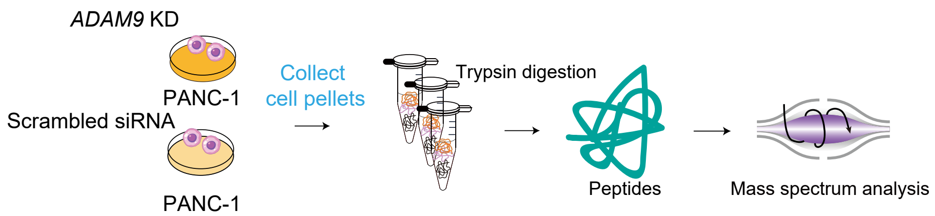

B

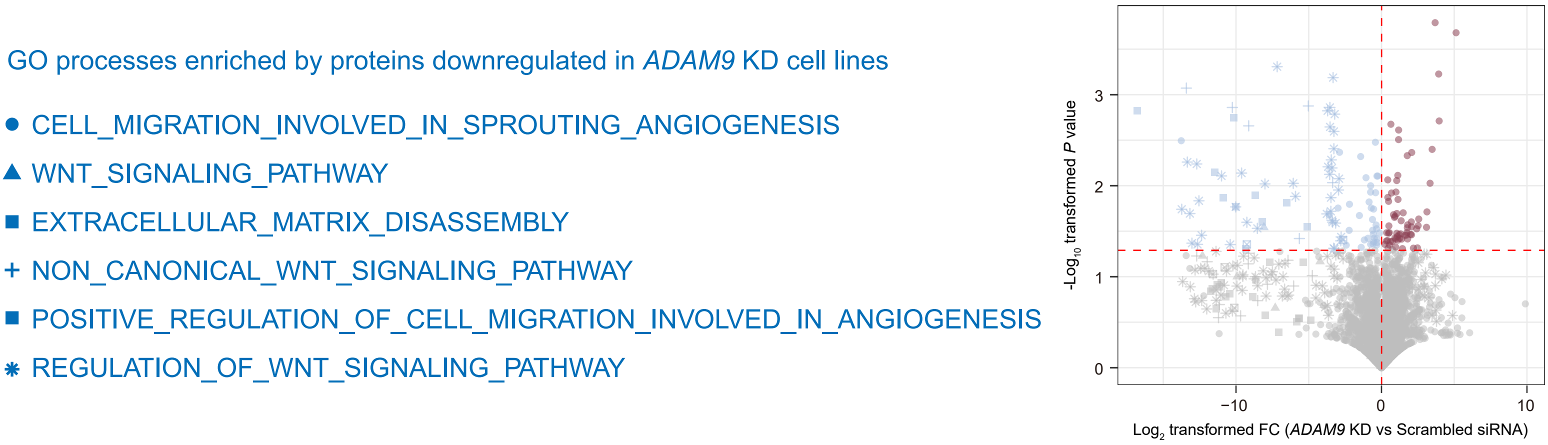

C

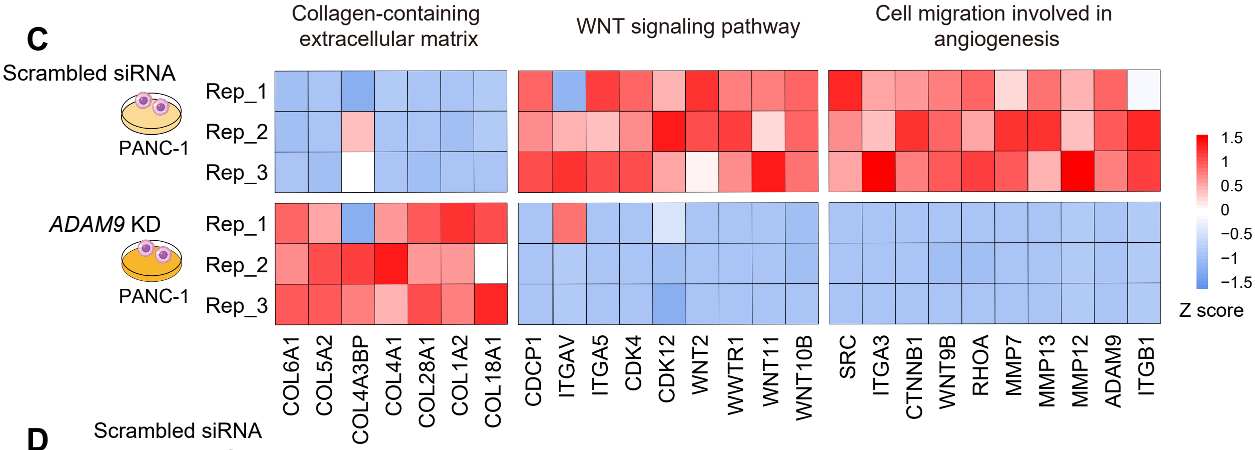

D

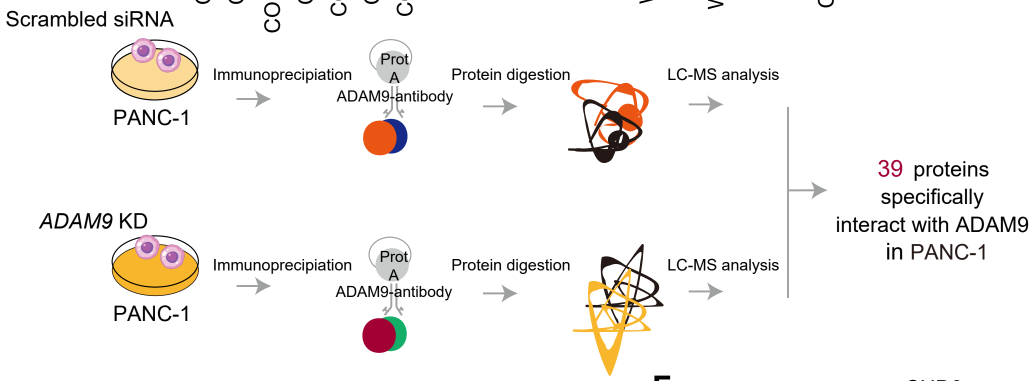

E

GO enriched by the proteins interact with ADAM9

Rank	Pathway	*P* value
1	Wnt signaling pathway	7.59E-07
2	TGF-beta signaling pathway	2.61E-07
3	VGFA-VEGFR signaling pathway	0.0000581
4	E-caherin signaling pathway	0.001122
5	Digradation of the ECM	0.001847

F

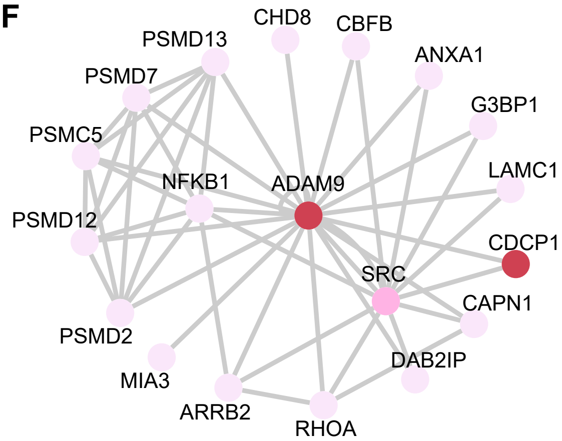

Supplement: Supplementary file 10 — Additional file 10: Fig. S10. Protein expression changes in PANC-1 cell lines after ADAM9 knocked down and the co-expressed proteins of ADAM9, related to Fig. 5. A The schematic work flow of validation experiments for the protein expression changes after ADAM9 knocked down. B The volcano plot showed the proteins that significantly altered between PANC-1 cells with ADAM9 KD and Scrambled siRNA. The GO processes enriched by proteins downregulated in ADAM9 KD cell lines are noted on the left. C Expression of proteins participated in collagen-containing extracellular matrix, WNT signaling pathway and cell migration involved in angiogenesis in the ADAM9 KD group and Scrambled siRNA group (n = 3 repeats per group). D The schematic work flow of co-immunoprecipitation assay of ADAM9 KD group and Scrambled siRNA group in PANC-1 cell lines. E Table showing GO processes enriched by the proteins interact with ADAM9. F Network of proteins interacting with ADAM9. [file 13045_2022_1384_MOESM10_ESM.pdf]
