# Supplementary material for: Proteogenomic insights into the biology and treatment of pancreatic ductal adenocarcinoma
Source: J Hematol Oncol. 2022 Nov 25;15:168. doi: 10.1186/s13045-022-01384-3 (PMC9701038; doi:10.1186/s13045-022-01384-3)
Supplement: Supplementary file 14 — Additional file 14: Fig. S14. Proteomic subtypes of CPTAC PDAC patients based on Fudan’s proteomic stratification, related to Fig. 6. A Consensus matrices of identified clusters (k = 2 to 5) of proteomic subtypes in CPTAC cohort based on Fudan’s proteomic stratification. B, C The consensus CDF (B) and delta area (change in CDF area) plots (C) are shown. D The heatmap depicting the relative abundance of signature proteins among three proteomic subtypes. E The association of three CPTAC proteomic subtypes based on our proteomic stratification with clinical outcomes. F Representative GO terms in CPTAC proteomic subtypes based on our proteomic stratification. G, H The boxplots indicating the protein expression of GRB7 (G), AKT2 and ERBB2 (H) among three proteomic subtypes in CPTAC cohort based on our proteomic stratification (Kruskal-Wallis test). I A volcano plot showing the prognosis associated characteristic proteins of three subgroups in CPTAC cohort based on our proteomic stratification. Red, proteins that negatively associated with prognosis in Fudan cohort; green, proteins that positively associated with prognosis in Fudan cohort. J The Sankey plot revealing the association between CPTAC subgroups and CPTAC subgroups based on our proteomic stratification. **** p < 1.0E-4, *** p < 1.0E-3, ** p < 1.0E-2, * p < 0.05, ns > 0.05. [file 13045_2022_1384_MOESM14_ESM.pdf]

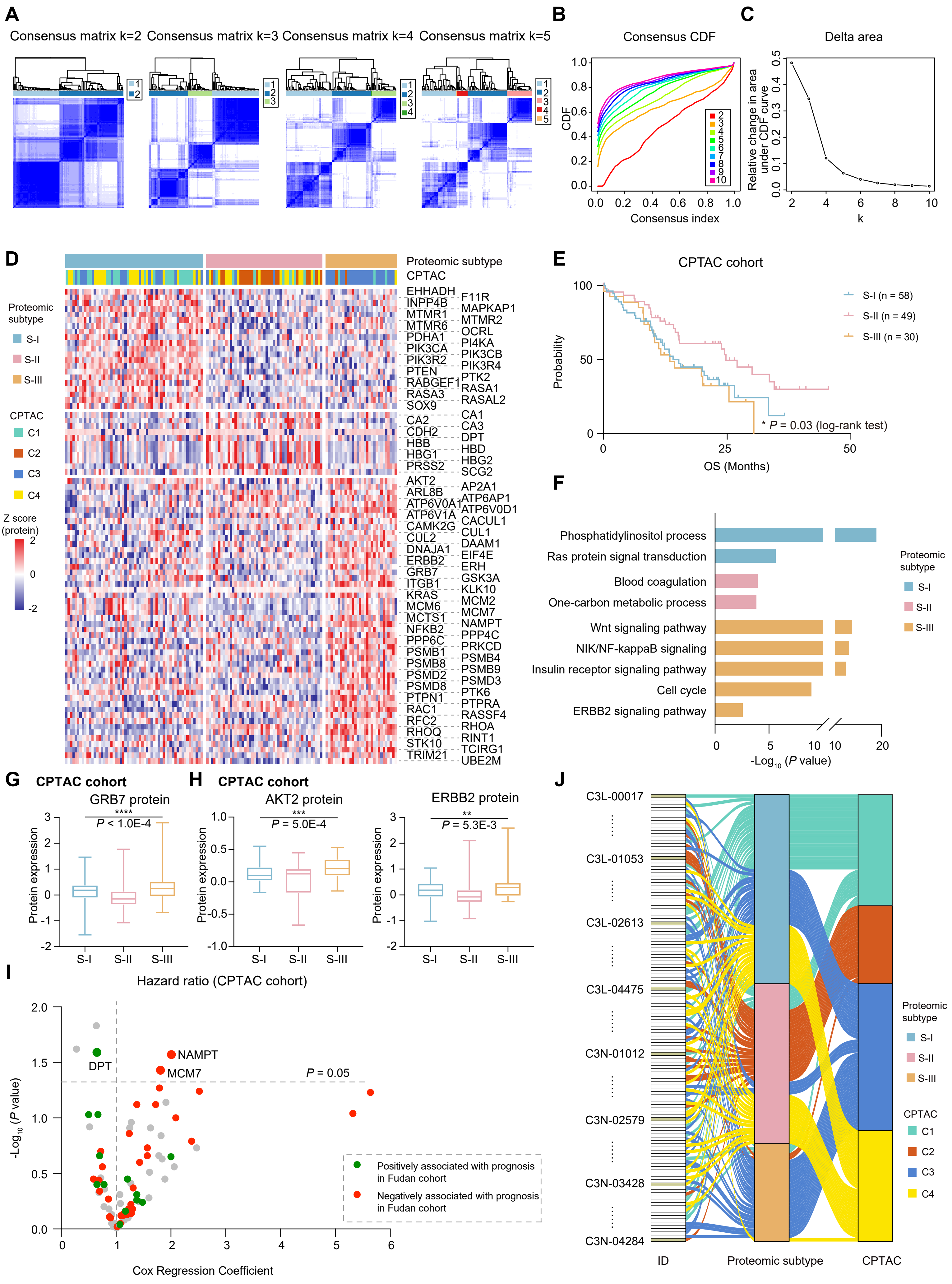
A
Consensus matrix k=2
Consensus matrix k=3
Consensus matrix k=4
Consensus matrix k=5
B
Consensus CDF
CDF
Consensus index
C
Delta area
Relative change in area under CDF curve
k
D
Proteomic subtype
CPTAC
S-I
S-II
S-III
C1
C2
C3
C4
Z score (protein)
EHHADH F11R INPP4B MAPKAP1 MTMR1 MTMR2 MTMR6 OCRL PDHA1 PI4KA PIK3CA PIK3CB PIK3R2 PIK3R4 PTEN PTK2 RABGEF1 RASA1 RASA3 RASAL2 SOX9
CA1 CA2 CA3 CDH2 DPT HBB HBD HBG1 HBG2 PRSS2 SCG2
AKT2 AP2A1 ARL8B ATP6AP1 ATP6V0A1 ATP6V0D1 ATP6V1A CACUL1 CAMK2G CUL1 CUL2 DAAM1 DNAJA1 EIF4E ERBB2 ERH GRB7 GSK3A ITGB1 KLK10 KRAS MCM2 MCM6 MCM7 MCTS1 NAMPT NFKB2 PPP4C PPP6C PRKCD PSMB1 PSMB4 PSMB8 PSMB9 PSMD2 PSMD3 PSMD8 PTK6 PTPN1 PTPRA RAC1 RASSF4 RFC2 RHOA RHOQ RINT1 STK10 TCIRG1 TRIM21 UBE2M
E
CPTAC cohort
Probability
S-I (n = 58)
S-II (n = 49)
S-III (n = 30)
* P = 0.03 (log-rank test)
OS (Months)
F
Phosphatidylinositol process
Ras protein signal transduction
Blood coagulation
One-carbon metabolic process
Wnt signaling pathway
NIK/NF-kappaB signaling
Insulin receptor signaling pathway
Cell cycle
ERBB2 signaling pathway
-Log10 (P value)
G
CPTAC cohort
GRB7 protein
****
P < 1.0E-4
Protein expression
H
CPTAC cohort
AKT2 protein
***
P = 5.0E-4
ERBB2 protein
**
P = 5.3E-3
I
Hazard ratio (CPTAC cohort)
-Log10 (P value)
NAMPT
DPT
MCM7
P = 0.05
Positively associated with prognosis in Fudan cohort
Negatively associated with prognosis in Fudan cohort
Cox Regression Coefficient
J
C3L-00017
C3L-01053
C3L-02613
C3L-04475
C3N-01012
C3N-02579
C3N-03428
C3N-04284
ID
Proteomic subtype
CPTAC
