# Supplementary material for: Proteogenomic insights into the biology and treatment of pancreatic ductal adenocarcinoma
Source: J Hematol Oncol. 2022 Nov 25;15:168. doi: 10.1186/s13045-022-01384-3 (PMC9701038; doi:10.1186/s13045-022-01384-3)
Supplement: Supplementary file 22 — Additional file 22: Fig. S22. Hematoxylin and eosin (H&E) staining on PDACs. The tumor cell purities of tumor tissues and the non-tumor cell purities of tumor-adjacent tissues. [file 13045_2022_1384_MOESM22_ESM.pdf]

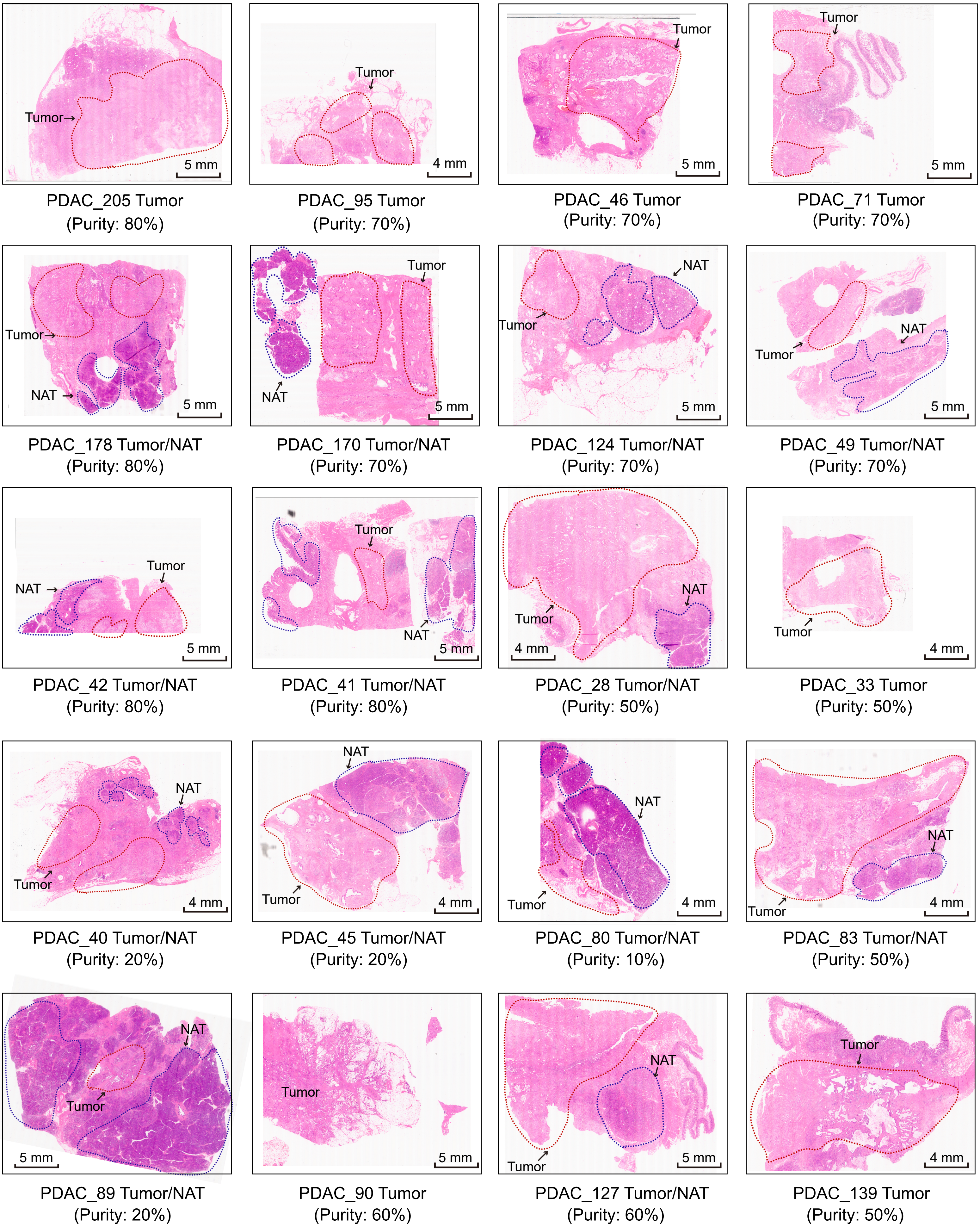
Tumor
5 mm
PDAC_205 Tumor
(Purity: 80%)
Tumor
4 mm
PDAC_95 Tumor
(Purity: 70%)
Tumor
5 mm
PDAC_46 Tumor
(Purity: 70%)
Tumor
5 mm
PDAC_71 Tumor
(Purity: 70%)
Tumor
NAT
5 mm
PDAC_178 Tumor/NAT
(Purity: 80%)
Tumor
NAT
5 mm
PDAC_170 Tumor/NAT
(Purity: 70%)
NAT
Tumor
5 mm
PDAC_124 Tumor/NAT
(Purity: 70%)
NAT
Tumor
5 mm
PDAC_49 Tumor/NAT
(Purity: 70%)
Tumor
NAT
5 mm
PDAC_42 Tumor/NAT
(Purity: 80%)
Tumor
NAT
5 mm
PDAC_41 Tumor/NAT
(Purity: 80%)
NAT
Tumor
4 mm
PDAC_28 Tumor/NAT
(Purity: 50%)
Tumor
4 mm
PDAC_33 Tumor
(Purity: 50%)
NAT
Tumor
4 mm
PDAC_40 Tumor/NAT
(Purity: 20%)
NAT
Tumor
4 mm
PDAC_45 Tumor/NAT
(Purity: 20%)
NAT
Tumor
4 mm
PDAC_80 Tumor/NAT
(Purity: 10%)
NAT
Tumor
4 mm
PDAC_83 Tumor/NAT
(Purity: 50%)
NAT
Tumor
5 mm
PDAC_89 Tumor/NAT
(Purity: 20%)
Tumor
5 mm
PDAC_90 Tumor
(Purity: 60%)
NAT
Tumor
5 mm
PDAC_127 Tumor/NAT
(Purity: 60%)
Tumor
4 mm
PDAC_139 Tumor
(Purity: 50%)
