# Supplementary material for: Proteogenomic insights into the biology and treatment of pancreatic ductal adenocarcinoma
Source: J Hematol Oncol. 2022 Nov 25;15:168. doi: 10.1186/s13045-022-01384-3 (PMC9701038; doi:10.1186/s13045-022-01384-3)
Supplement: Supplementary file 12 — Additional file 12: Fig. S12. Multi-omics characteristics of the three proteomic subtypes, related to Fig. 6. A Bar plot showing the number of overexpressed molecules in each proteomic subtypes at both proteome and transcriptome level. B Heatmap of the characteristic molecules of each proteomic subtypes at proteome (left) and transcriptome (right) level. Molecules previously reported to be associated with PDAC are marked in red. C Scatter plot displaying the significance of molecules that highest expressed in S-III at RNA level (y axis) and at protein level (x axis). Enriched biological pathways are showed on the right. D The systematic diagram summarizing significantly altered proteins that enriched in ErbB_EGFR signaling pathway. Color of each cell shows Z-scored average abundance of the protein among proteome subtypes. E The kinome tree of three proteomic subtypes. F Coxcomb diagrams showing the distribution of 10 kinase family in three proteomic subtypes. G The association of kinase-substrate pairs overrepresented in proteomic subtypes. **** p < 1.0E-4, *** p < 1.0E-3, ** p < 1.0E-2, * p < 0.05, ns > 0.05. [file 13045_2022_1384_MOESM12_ESM.pdf]

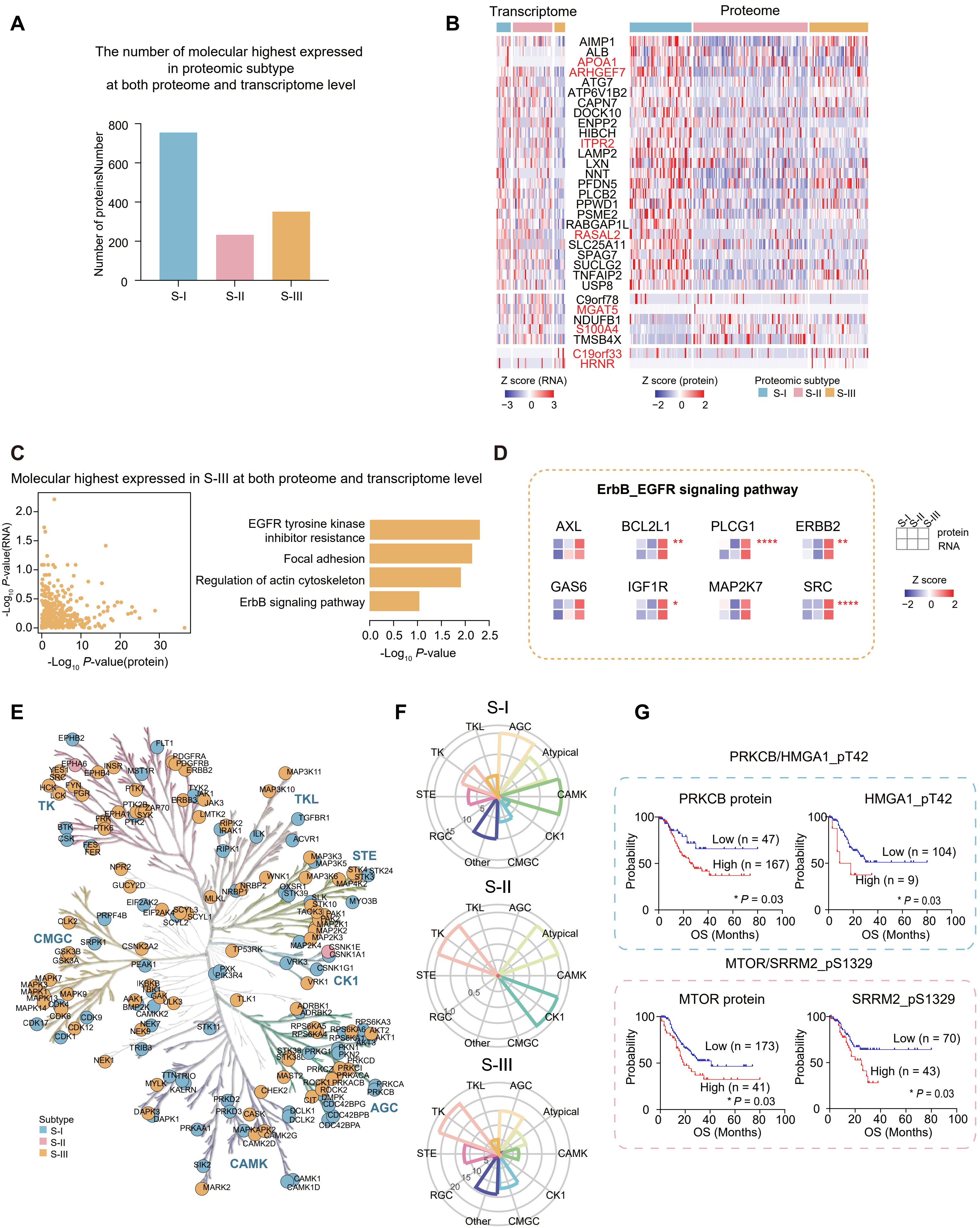

A
The number of molecular highest expressed in proteomic subtype at both proteome and transcriptome level
Number of proteinsNumber
0
200
400
600
800
S-I
S-II
S-III
B
Transcriptome
Proteome
AIMP1
ALB
APOA1
ARHGEF7
ATG7
ATP6V1B2
CAPN7
DOCK10
ENPP2
HIBCH
ITPR2
LAMP2
LXN
NNT
PFDN5
PLCB2
PPWD1
PSME2
RABGAP1L
RASAL2
SLC25A11
SPAG7
SUCLG2
TNFAIP2
USP8
C9orf78
MGAT5
NDUFB1
S100A4
TMSB4X
C19orf33
HRNR
Z score (RNA)
−3 0 3
Z score (protein)
−2 0 2
Proteomic subtype
S-I S-II S-III
C
Molecular highest expressed in S-III at both proteome and transcriptome level
-Log10 P-value(RNA)
-Log10 P-value(protein)
0.0
0.5
1.0
1.5
2.0
0
10
20
30
EGFR tyrosine kinase inhibitor resistance
Focal adhesion
Regulation of actin cytoskeleton
ErbB signaling pathway
0.0 0.5 1.0 1.5 2.0 2.5
-Log10 P-value
D
ErbB_EGFR signaling pathway
AXL
BCL2L1 **
PLCG1 ****
ERBB2 **
GAS6
IGF1R *
MAP2K7
SRC ****
S-I S-II S-III
protein
RNA
Z score
−2 0 2
E
TK
TKL
STE
CK1
CMGC
CAMK
AGC
Subtype
S-I
S-II
S-III
EPHB2
FLT1
PDGFRA
PDGFRB
ERBB2
EPHA6
INSR
MST1R
YES1
SRC
EPHB4
HCK
FYN
LCK
FGR
PTK7
TYK2
JAK1
ERBB3
JAK3
PTK2B
ZAP70
EPHA1
SYK
FRK
PTK6
PTK2
LMTK2
BTK
CSK
FES
FER
RIPK2
IRAK1
ILK
RIPK1
MAP3K11
MAP3K10
TGFBR1
ACVR1
MAP3K3
MAP3K5
STK4
STK24
STK3
MAP3K6
MAP4K2
WNK1
NPR2
GUCY2D
NRBP2
NRBP1
OXSR1
STK39
MLKL
EIF2AK2
EIF2AK4
SCYL3
SCYL1
SCYL2
SLK
STK10
MYO3B
TAOK3
PAK1
PAK2
MAP2K1
MAP2K2
MAP2K3
MAP2K4
CLK2
PRPF4B
SRPK1
CSNK2A2
TP53RK
CSNK1E
CSNK1A1
GSK3B
GSK3A
PEAK1
VRK3
CSNK1G1
PXK
PIK3R4
VRK1
MAPK7
MAPK3
MAPK1
MAPK13
MAPK9
MAPK14
CDK4
IKBKB
TBK1
GAK
AAK1
BMP2K
ULK3
TLK1
CDK6
CAMKK2
CDK9
CDK17
CDK12
CDK1
NEK7
NEK9
STK11
TRIB3
NEK1
ADRBK1
ADRBK2
RPS6KA5
RPS6KA4
RPS6KA6
RPS6KA3
RPS6KA1
AKT2
AKT3
AKT1
STK38
STK38L
PRKG1
PKN1
PKN2
PRKCD
MAST2
PRKCZ
PRKCI
PRKACA
PRKCA
PRKCB
TTN
TRIO
MYLK
KALRN
CHEK2
ROCK1
PRKACB
ROCK2
CIT
DMPK
CDC42BPG
CDC42BPB
CDC42BPA
PRKD2
PRKD3
CASK
DCLK1
DCLK2
DAPK3
DAPK1
PRKAA1
MAPKAPK2
CAMK2G
CAMK2D
SIK2
CAMK1
CAMK1D
MARK2
F
S-I
S-II
S-III
TKL
AGC
TK
Atypical
STE
CAMK
RGC
CK1
Other
CMGC
5
10
15
0.5
1.0
20
G
PRKCB/HMGA1_pT42
PRKCB protein
HMGA1_pT42
Low (n = 47)
High (n = 167)
* P = 0.03
Low (n = 104)
High (n = 9)
* P = 0.03
MTOR/SRRM2_pS1329
MTOR protein
SRRM2_pS1329
Low (n = 173)
High (n = 41)
* P = 0.03
Low (n = 70)
High (n = 43)
* P = 0.03
Probability
OS (Months)
0
50
100
0 20 40 60 80 100
